# A Novel Approach for Pollen Identification and Quantification Using Hybrid Capture‐Based DNA Metabarcoding

**DOI:** 10.1002/ece3.71311

**Published:** 2025-04-23

**Authors:** D. Kireta, K.‐J. van Dijk, S. Crotty, A. Malik, K. Bell, K. Hogendoorn, A. J. Lowe

**Affiliations:** ^1^ School of Biological Sciences The University of Adelaide Adelaide South Australia Australia; ^2^ Department of Life Sciences The University of Trieste Trieste Italy; ^3^ School of Computer and Mathematical Sciences The University of Adelaide Adelaide South Australia Australia; ^4^ New South Wales Department of Primary Industries Wagga Wagga New South Wales Australia; ^5^ School of Biological Sciences University of Western Australia Perth WA Australia; ^6^ School of Agriculture, Food and Wine The University of Adelaide Adelaide South Australia Australia

**Keywords:** hybrid capture, pollen identification, pollen metabarcoding, pollen quantification, target enrichment

## Abstract

Pollen identification (ID) and quantification is important in many fields, including pollination ecology and agricultural sciences, and efforts to explore optimal molecular methods for identifying low concentrations of DNA from plant mixtures are increasing, but quantifying mixture proportions remains challenging. Traditional pollen ID using microscopy is time‐consuming, requires expertise and has limited accuracy and throughput. Molecular barcoding approaches being explored offer improved accuracy and throughput. The common approach, amplicon sequencing, employs PCR amplification to isolate DNA barcodes, but introduces significant bias, impairing downstream quantification. We apply a novel molecular hybrid capture approach to artificial pollen mixtures to improve upon current taxon ID and quantification methods. The method randomly fragments DNA and uses RNA baits to capture DNA barcodes, which allows for PCR duplicate removal, reducing downstream quantification bias. Four reference databases were used to explore identification and quantification. A restricted *matK* database containing only mixture species yielded sequence proportions highly correlated with input pollen proportions, demonstrating the potential usefulness of hybrid capture for metabarcoding and quantifying pollen mixtures. Identification power was further tested using two reference libraries constructed from publicly available sequences: the *matK* plastid barcode and RefSeq complete chloroplast references. Single barcode‐based taxon ID did not consistently resolve to species or genus level. The RefSeq chloroplast database performed better qualitatively but had limited taxon coverage (relative to species used here) and introduced ID issues. At the family level, both databases yielded comparable qualitative results, but the RefSeq database performed better quantitatively. Whilst the method developed here has tremendous potential, the choice and expansion of reference databases remains one of the most important factors allowing qualitative and quantitative accuracy using the full set of genomic regions screened by this hybrid capture method.

## Graphical abstract

This study explored hybrid capture as an alternative approach for pollen identification and quantification, aiming to overcome biases introduced by PCR‐based metabarcoding. Identification was tested on artificial pollen mixtures using two reference databases, and we find potential for pollen identification and improved quantification.

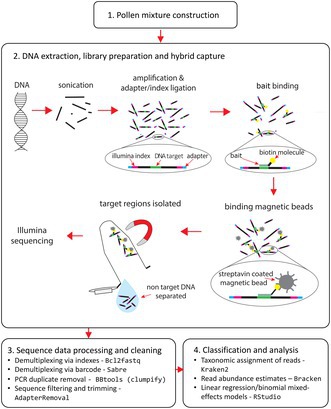

## 1 Introduction

Pollen identification (ID) is important for many scientific fields. Traditional methods of pollen ID rely on microscopy to observe diagnostic characters on the pollen exine. This method is time‐consuming and requires considerable expertise, limits accuracy and throughput, and potentially constrains projects. The limitations of microscopy‐based pollen ID are well established (e.g., Bell et al. 2016; Kraaijeveld et al. 2015; Richardson, Lin, Sponsler et al. 2015; Smart et al. 2017), and thus, alternative methods for pollen ID have been sought.

DNA barcoding, or metabarcoding (mixed samples) has advanced taxon ID in many research fields, has been explored extensively for pollen ID, and has been shown to provide accurate identifications at high taxonomic resolution and with high sample throughput (Bell et al. 2019; Bell, Turo et al. 2022; Bell, Fowler et al. 2017; de Vere et al. 2017; Keller et al. 2015; Kraaijeveld et al. 2015; Richardson, Lin, Quijia et al. 2015; Richardson, Lin, Sponsler et al. 2015; Suchan et al. 2019; Wilson et al. 2010). Many more taxa can be IDed to at least genus level compared with microscopy‐based methods (Keller et al. 2015; Milla et al. 2021; Richardson, Lin, Sponsler et al. 2015).

The accuracy of metabarcoding is limited, however, by the choice of barcode and comprehensiveness of reference databases, since only taxa with reference sequences can be detected and identified. The Consortium for the Barcode of Life (CBOL) Plant Working Group recommends the chloroplast genome encoded *matK* (maturase K) and *rbcL* (ribulose 1,5‐biphosphate carboxylase) as standard barcodes which can ID approximately 70% of all plant taxa to species, given available references (CBOL Plant Working Group 2009). While standard barcodes, *matK* and *rbcL*, are rarely used in metabarcoding due to their long amplicon size, poor primer universality and the low abundance of plastid DNA in pollen (Bell, Loeffler et al. 2017; W. J. Kress and Erickson 2007). Alternative markers such as ITS2 (internal transcribed spacer 2) have further been proposed as universal plant barcodes due to their higher variability and utility in resolving taxonomic relationships at finer scales (Yao et al. 2010). While others, such as the *psbA‐trnH* spacer, have been recommended for specific groups of plants, or as supplementary barcodes (W. J. e. Kress and Erickson 2012). Standard barcodes require both sequence variability for taxonomic resolution and conserved primer binding sites for broad taxonomic coverage. The common barcoding approach uses PCR to amplify the barcode, followed by sequencing and comparison to a reference database. When references for target species are absent, similarity to the closest sequences in the database can generate a genus or family ID (Liu et al. 2019).

Despite the strengths of metabarcoding, its ability to provide quantitative insights into sample composition remains problematic. The relative proportions of taxa in pollen samples offer valuable insights into pollinator preferences and ecosystem dynamics (Dormontt et al. 2018). While several studies have reported correlations between sequencing read proportions and input pollen proportions for barcodes such as *trnL* (transfer RNA leucine), ITS1 (internal transcribed spacer 1) (Pornon et al. 2016), ITS2 (Keller et al. 2015), *rbcL* and *matK* (Richardson, Lin, Quijia et al. 2015), these correlations are often weak or inconsistent—particularly for ITS2 (Bell et al. 2019; Lamb et al. 2019; Smart et al. 2017). This is problematic given that ITS2 generally provides higher taxonomic resolution than other standard barcodes, which tend to perform even less well in quantification. As a result, amplicon‐based methods currently offer limited capacity to accurately quantify pollen mixture compositions.

Several sources of bias contribute to this challenge, affecting both the accuracy of taxonomic identification and the reliability of quantification. These include limited barcode resolution and database quality (Richardson et al. 2017); variation in differences in DNA isolation methods (Pornon et al. 2016); differential amplification due to primer affinity (Dowle et al. 2016; Krehenwinkel et al. 2017), false negatives due to poor amplification (Pawluczyk et al. 2015; Zinger et al. 2019), variations in barcode copy number (Krehenwinkel et al. 2017), DNA degradation bias (Krehenwinkel et al. 2018); and sequencing bias (Pawluczyk et al. 2015). The most substantial issues likely arise from unequal PCR amplification—especially among closely related taxa—and variation in chloroplast genome copy number (Golczyk et al. 2014), both of which can result in up to 2000‐fold differences in DNA abundance post‐PCR (Pawluczyk et al. 2015). These factors undermine the use of amplicon metabarcoding for reliable quantification.

PCR‐free methods are being explored as a means to overcome these quantitative challenges, and they show improvement in quantification over PCR‐based metabarcoding, for example, genome skimming and chloroplast assembly (Lang et al. 2019), Whole Genome Shotgun sequencing (Bell et al. 2021), and MinION Reverse Metagenomics (Peel et al. 2019). However, these methods have other drawbacks. Genome skimming and Whole Genome Sequencing (WGS) for example, require a larger amount of DNA, which can be difficult to obtain from small solitary pollinators (Bell et al. 2021; Lang et al. 2019), and MinION Reverse Metagenomics requires the user to curate their own reference databases (Peel et al. 2019).

An alternative method that could overcome these shortcomings and improve accuracy and quantification is hybrid capture. Hybrid capture is a target enrichment technique that has been applied to environmental and ecological studies, including biomonitoring, where it has been shown to improve taxon detection and mitigate biases associated with PCR‐based methods (Dowle et al. 2016). The approach has also been successfully applied to metabarcoding of mixed environmental or bulk samples to improve species detection, and has been proposed as a promising alternative to PCR‐based methods in studies where improved recovery of taxa is critical (Foster et al. 2021; Li et al. 2023; Schulte et al. 2021; Wilcox et al. 2018). It is effective on degraded DNA and has been used to create a reference database from herbarium specimens (Dormontt et al. 2018), explore historic ecological communities through sediment cores (Foster et al. 2021; Schulte et al. 2021), and infer phylogenetic relationships for example, (Nge et al. 2021). In amplicon metabarcoding, exact copies of barcodes are created that cannot easily be distinguished from PCR duplicates, whereas hybrid capture approaches avoid this issue by circumventing the need for PCR amplification (Dowle et al. 2016).

Our approach used sonication to randomly fragment DNA after extraction, creating a pool of DNA fragments of variable lengths. A genomic library was generated and chloroplast loci were enriched by using RNA baits complementary to the sites of interest (Waycott et al. 2021). Given that each DNA fragment is theoretically unique, PCR duplicates can be easily eliminated bioinformatically, retaining only one copy of every sequenced read or read pair. This allows downstream quantification of relative taxon abundances based on the number of reads mapping to references.

This study aimed to demonstrate the effectiveness of hybrid capture DNA metabarcoding for identifying taxa in a pollen mixture. It also aimed to assess the accuracy of relative taxonomic abundance estimations to potentially apply this in broader pollination research applications. We used two reference databases: a *matK* database commonly used in amplicon metabarcoding (and a locus targeted by this bait set) and a RefSeq whole chloroplast database. We expect that the RefSeq database will produce more accurate qualitative and quantitative results since more informative gene regions can be recovered.

## 2 Materials and Methods

### 2.1 Sample Collection

A comprehensive experimental setup was made using pollen from three species from different families. The pollen from these taxa was visually distinct for easy morphological identification by non‐experts (Figure 2). This ensured that the taxa comprising each pollen pellet could be verified through morphology. Pollen was obtained from honey bee hives fitted with pollen traps. Honey bees forage on one species per foraging trip, so pollen pellets are usually comprised of a single species (Grüter and Ratnieks 2011; Synge 1947; Visscher and Seeley 1982). The hives had been placed in almond orchards ( *Prunus dulcis* ), brown stringybark plantations ( *Eucalyptus baxteri* ), and in a field with flowering capeweed ( *Arctotheca calendula* ). *Arctotheca calendula* pollen is a distinctive orange color and is easily separated from pollen pellets of other species that were present at the time of collection.

### 2.2 Pollen Mixtures

We constructed 14 different pollen mixtures, with three replicates of each mixture. We used four negative controls (blanks), one for each extraction batch, totaling 48 samples/libraries.

The pollen mixture proportions were weight based. Each taxon varied in proportion from high to low abundance (Table 1, Figure 3). The mixtures were suspended in ethanol and divided into three replicates for DNA extraction. Ethanol was used for suspension because it evaporates without leaving any residues that may affect subsequent DNA extractions and library preparations. Care was taken to strongly agitate the mixture before aliquoting.

**TABLE 1:** Mixed model with binomial distribution to determine if starting pollen proportion affected the success or failure of taxonomic identification to three taxonomic levels in pollen mixtures.

Barcode db	Taxonomic level	Mix taxa	Est.	S.E.	*Z*	*p*
*matK* restricted	Species	*E. baxteri*	14.42	19.72	0.73	0.46
		*A. calendula*	−7.20	6.81	−1.06	0.29
		*P. dulcis*	Response is constant			
*matK* wide	Species	*E. baxteri*	14.43	19.72	0.73	0.46
		*A. calendula*	−11.20	35.72	−0.31	0.75
		*P. dulcis*	−7.13	6.83	−1.04	0.30
	Genus	*E. baxteri*	14.30	19.91	0.79	0.47
		*A. calendula*	−11.20	35.72	−0.31	0.75
		*P. dulcis*	−7.13	6.83	−1.04	0.30
	Family	*E. baxteri*	14.43	19.72	0.73	0.46
		*A. calendula*	−7.20	6.82	−1.06	0.29
		*P. dulcis*	−7.13	6.83	−1.04	0.30
RefSeq restricted	Species	*E. baxteri*	Response is constant			
		*A. calendula*				
		*P. dulcis*				
RefSeq wide	Species	*E. baxteri*	9.03	6.85	1.32	0.19
		*A. calendula*	−1.98	2.51	−0.79	0.43
	Genus	*P. dulcis*	Response is constant			
		*E. baxteri*	14.43	19.72	0.73	0.46
		*A. calendula*	−2.03	2.58	−0.78	0.43
		*P. dulcis*	Response is constant			
	Family	*E. baxteri*	Response is constant			
		*A. calendula*				
		*P. dulcis*				

### 2.3 DNA Extraction and Library Preparation

**FIGURE 1:**
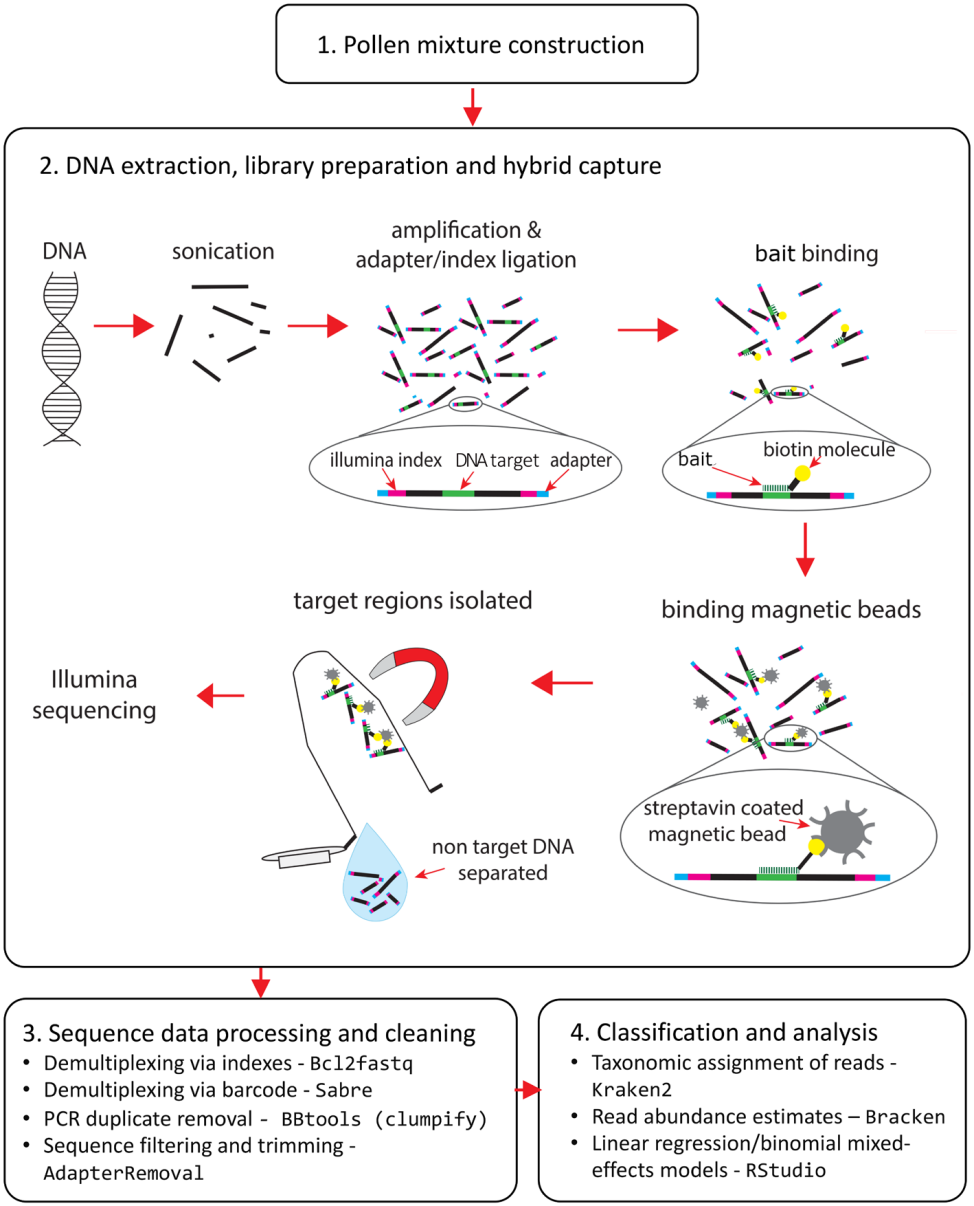
Laboratory and analysis workflow.

The laboratory and analysis steps described below are represented in Figure 1. DNA was extracted from the pollen mixtures (9 mg) using the NucleoSpin Food kit (Macherey‐Nagel, Düren, Germany), with the ‘isolation of genomic DNA from honey or pollen’ supplementary protocol with some modifications. The dry pollen mix aliquots were homogenised using ceramic beads using 2 mL screw cap tubes on a Bead Ruptor 24 (OMNI International Inc.) at 6 ms for 20 s cycles (3–4 min total) until a powder was formed. Sample tubes were submerged in liquid nitrogen before and between mill cycles to prevent DNA degradation during beating, and to aid homogenisation by increasing pollen brittleness. The final elution step was done by passing the 60 μL of elution buffer through the spin column membrane twice instead of once, followed by a centrifuge step to maximise DNA yield. Following extraction, DNA was quantified on a Quantus Fluorometer and QuantiFluor dsDNA System (Promega, Madison, WI, USA), normalised to 2 ng/μL (samples with concentration lower than 2 ng/μL were used neat), and sonicated using a Bioruptor Pico (Diagenode, USA) to create random length fragments (eight cycles of 15 s on, 90 s off).

**FIGURE 2:**
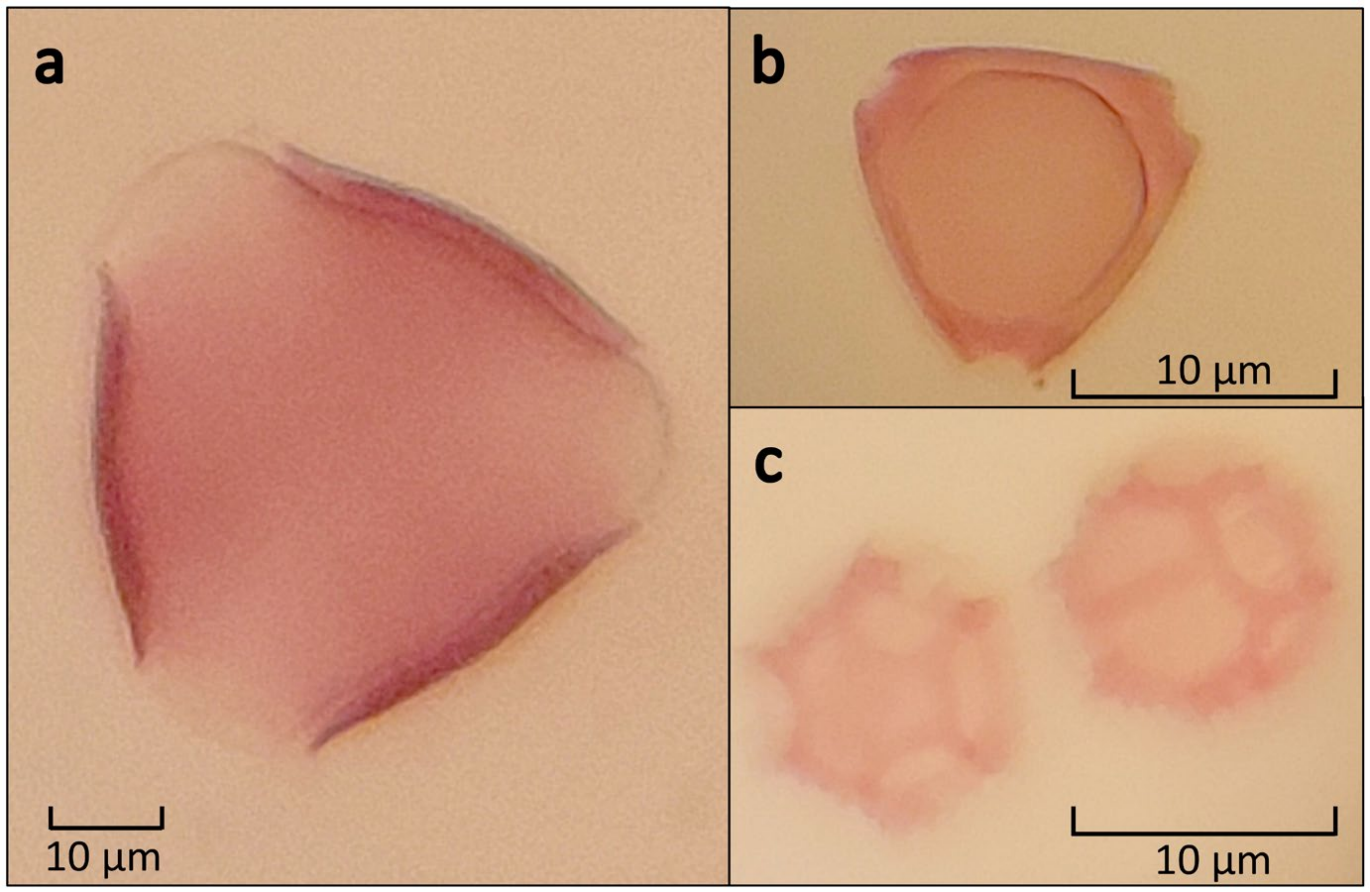
Images of the taxa of pollen used in artificial mixtures. (a) *Prunus dulcis* , (b) *Eucalyptus baxteri* , (c) *Arctotheca calendula* . Photographs were taken from slides under a compound microscope by Leif Currie.

**FIGURE 3:**
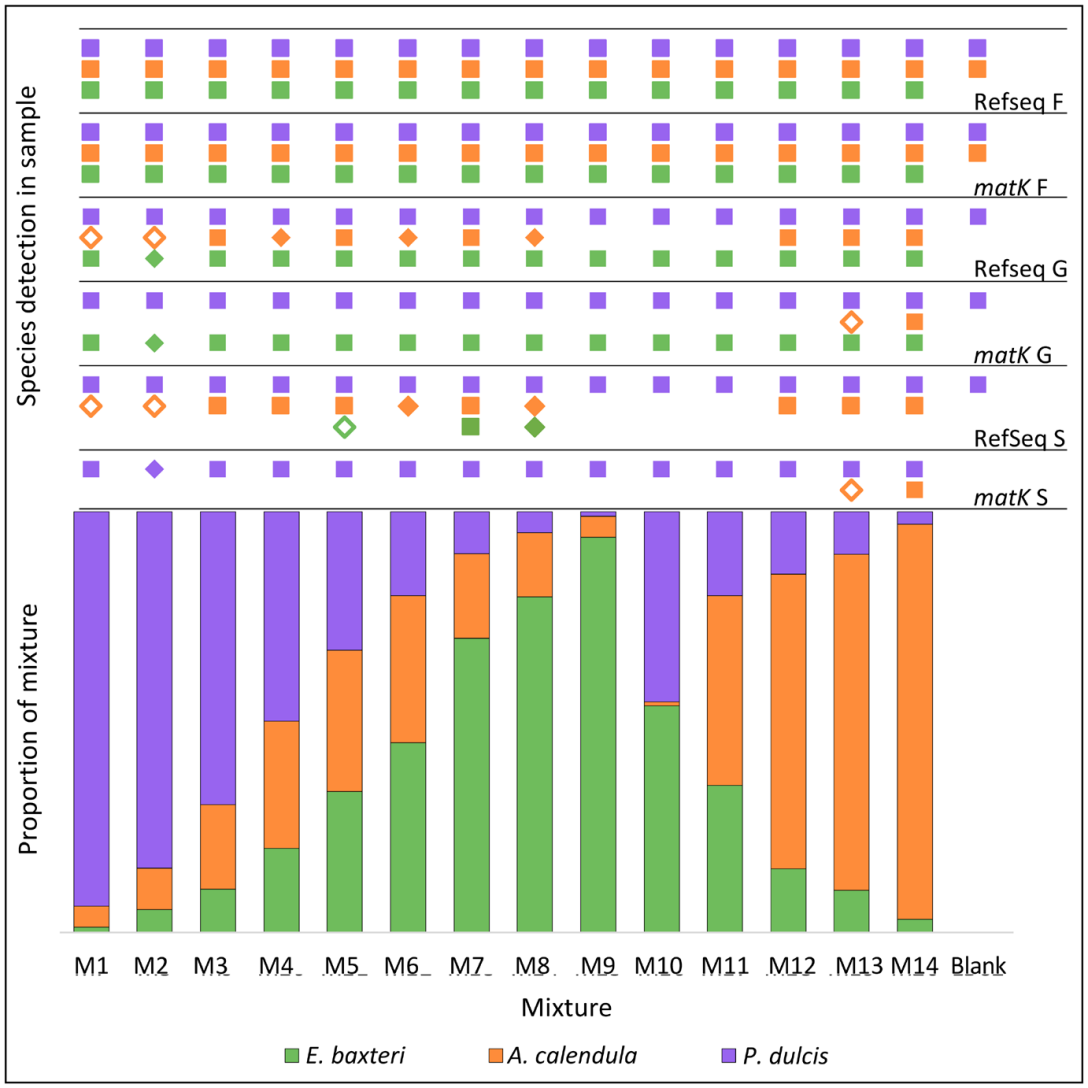
Stacked bar plot of the relative input proportions by weight of three pollen taxa ( *Prunus dulcis* , *Arctotheca calendula* and *Eucalyptus baxteri* ) in artificially constructed mixtures (M1—M14), and negative control (Blank). Symbols above each bar indicate whether each taxon was detected in the mixture using metabarcoding with either *matK* or RefSeq databases, identified to family (F), genus (G) and species (S) levels. Solid squares indicate the taxon was detected in three mixture replicates, solid diamonds indicate detection in two of the three replicates, and hollow diamonds indicate detection in only one replicate.

**FIGURE 4:**
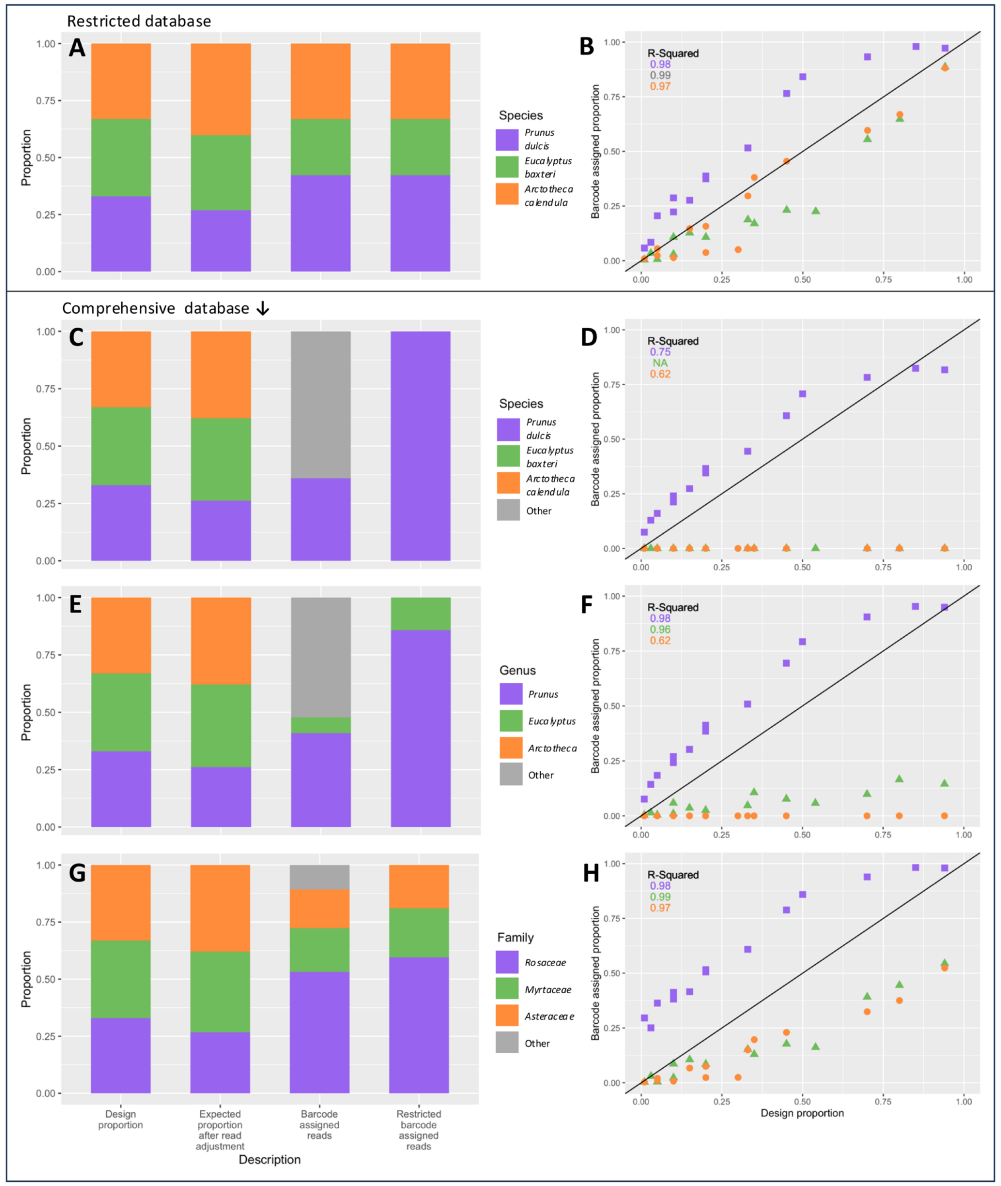
Plots depicting taxonomic assignment in pollen mixtures using a *matK* reference database. (A, B) Taxonomic assignment to species level made using a restricted *matK* database (only containing three taxa used in mixtures); (C, D) Assignment to species level using a comprehensive *matK* database; (E, F) Assignment to genus level using a comprehensive *matK* database; (G, H) Assignment to family level using a comprehensive *matK* database. *Left side*: Summary of taxon proportions averaged across samples. Columns from left to right are: (1) original design proportion according to pollen weight, (2) expected proportion after read correction (given the 14 mixtures had different numbers of reads per taxon), (3) total barcode assigned reads, (4) barcode assigned reads with ‘other’ (non‐target) taxa excluded. Right side: Sequence proportions versus input (design) proportions.

**FIGURE 5:**
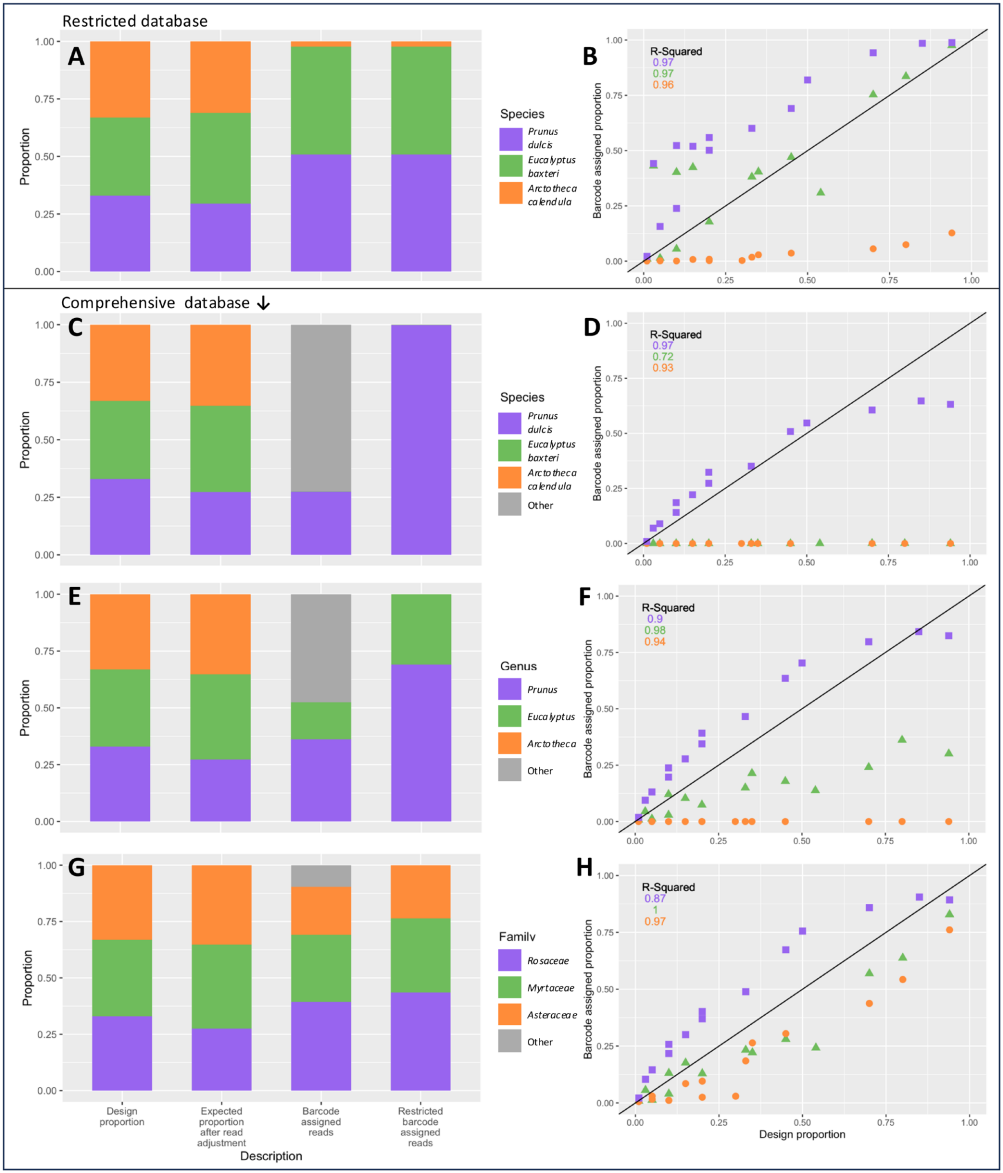
Plots depicting taxonomic assignment in pollen mixtures using a RefSeq reference database. (A, B) Assignment to species level made using a restricted database (only containing three taxa used in mixtures); (C, D) Assignment to species level using a comprehensive database; (E, F) Assignment to genus level using a comprehensive RefSeq database; (G, H) Assignment to family level using a comprehensive database. *Left side*: Summary of taxon proportions averaged across samples. Columns from left to right are: (1) original design proportion according to pollen weight, (2) expected proportion after read correction (given the 14 mixtures had different numbers of reads per taxon), (3) total barcode assigned reads, (4) barcode assigned reads with ‘other’ (non‐target) taxa excluded. *Right side*: Sequence proportions versus input (design) proportions of pollen.

Library preparation was done using an Eppendorf epMotion 5075 t Liquid Handling Workstation. The DNA libraries were prepared using the NEBNext Ultra II DNA Library Prep kit as described in the protocol by Waycott et al. (2021). In brief, custom‐made stubby Y‐adaptors were ligated to the DNA fragments. Each adaptor contained one of 48 unique 8‐nucleotide in‐line barcodes allowing downstream sample pooling. The libraries were amplified by PCR (30 s at 94°C, followed by 17 cycles of 98°C for 10 s, 65°C for 30 s and 72°C for 30 s, a final extension at 72°C for 2 min and held at 4°C). To reduce costs associated with reagents and sequencing, libraries were pooled into groups of 16 samples based on estimated library concentrations, maximising target enrichment efficiency and minimising the number of reactions needed. Pools were purified using a 1:1 volume concentration of MagNA Beads (Rohland and Reich 2012).

The number of PCR cycles prior to hybrid capture was determined during pilot testing and adjusted to account for the loss of non‐target DNA following enrichment, which results in non‐measurable DNA concentrations. We therefore increased the cycle number to ensure enough material for hybridisation and subsequent quantification and sequencing. While this increases PCR duplicates, we mitigated this bioinformatically.

### 2.4 Hybrid Capture

This study used the OZBaits_CP V1.0 universal plastid bait set for hybrid capture developed for targeted capture of angiosperm sequences (Waycott et al. 2021), following the myBaits Targeted NGS Manual Version 4.01 hybridization protocol. The baits were designed to target 19 chloroplast genes, including MatK, applicable to all angiosperm lineages (details available in Supplementary Table S2, Waycott et al. 2021). To ensure suitability for a broad range of taxa, only 80%–90% similarity is needed to retrieve the target, and specificity can be controlled by altering the hybridization temperature (Waycott et al. 2021).

Hybrid capture was done at 65°C for 48 h. To reduce evaporation, chill‐out red liquid wax was added (Bio‐Rad Laboratories Inc.). Enriched libraries were then amplified (2 min at 98°C, followed by 20 cycles of 98°C for 20 s, 60°C for 30 s and 72°C for 45 s, a final extension at 72°C for 5 min and held at 8°C) with custom indexed P7 and P5 Illumina adaptors. The resulting libraries were visualised and quantified using high‐sensitivity DNA ScreenTapes on a 2100 Bioanalyzer (Agilent) and pooled in equimolar concentrations. The final library was purified using 1:1 MagNA, and a final size selection to 350–600 bp was done on a PippinPrep using a 2% agarose gel cassette with Marker L (Sage Science).

The combination of in‐line molecular identifiers (adapter barcodes) and dual indexes was used only once for any library preparation in our lab to enable contamination tracing. The final library was sequenced at the Garvan Institute of Medical Research (Sydney, Australia) on one lane of an Illumina HiSeq X Ten with 2 × 150 cycle chemistry.

### 2.5 Bioinformatics Pipeline: Sequence Data Processing and Cleaning

Analyses were done using the Phoenix high‐performance computing cluster at the University of Adelaide, Australia. Samples were first demultiplexed via the indexes using Bcl2fastq, then demultiplexed into their sample‐specific reads with the internal barcodes using Sabre (Sabre‐barcode‐demultiplexing, n.d.). The barcodes were designed with at least 2° of separation, so one base pair mismatch was allowed.

We explored several analysis methods, including the pipelines developed by Sickel et al. (2015) and Bell et al. (2021) which were developed for metabarcoding and WGS, respectively. However, we were unsuccessful in implementing methods using qiime2, which appeared incompatible with our non‐amplicon data (we also attempted to use the q2‐shotgun and q2‐metaphlan2 plugins for shotgun data, but were unable to overcome the issues encountered). We ultimately used customised steps, similar to those of Bell et al. (2021), but used modified pre‐processing steps, and additionally used Bracken (Lu et al. 2017) (see below) for improved quantification. We removed PCR duplicates using Clumpify from BBtools (Bushnell 2021). Clumpify operates by grouping reads that have identical sequences and mapping coordinates and does not require assembly. Removing PCR duplicates also made subsequent analyses faster and less memory intensive, since the dataset had been reduced by more than half. Sequence filtering and trimming were done using AdapterRemoval (Schubert et al. 2016). The 9th base following the 8 nt barcode (residual A‐tail of library prep), reads shorter than 30 nt, reads with a phred quality score < 20, and N tails were removed. This was followed by Kraken2 to assign taxonomy to reads using a k‐mer based method, which involves breaking sequences into k‐mers and matching these against a database, applying the Lowest Common Ancestor (LCA) algorithm to determine the best taxonomic classification, without needing pre‐assembly of the sequences (Wood et al. 2019). Kraken2 classified the reads at species, genus and family levels. Bracken was then used to estimate read abundance using the Kraken2 classifications (Lu et al. 2017). The default parameters were used for both steps, with the exception of adjusting the minimum hit group threshold to 5 in Kraken2 (which is beneficial when using custom databases) and a threshold of 5 set in Bracken. The Bracken output was analysed using R (RStudio Team 2020).

We explored different reference database approaches for taxonomic identification, the first using a *matK* single barcode database, and the second using a complete chloroplast RefSeq database. The databases were downloaded (January 2022) and built using Kraken2 and Bracken. A list of all angiosperm species recorded in South Australia, including introduced species, was obtained from the Atlas of Living Australia (https://www.ala.org.au/). The publicly available reference sequences for *matK* were downloaded for this list. The RefSeq database consisted of all angiosperm chloroplast records available from the NCBI RefSeq database. *Arctotheca calendula* did not have a RefSeq chloroplast reference, so the chloroplast sequences available on NCBI were manually added to the database to ensure all taxa used in the mixtures were represented. At the time, 15 chloroplast sequences from 8 gene regions were available (Table S1), and of the 8 regions, 6 matched barcodes targeted by the chloroplast bait set (Waycott et al. 2021). For both databases, a reduced version was created which contained only the three taxa present in the pollen mixtures, to test quantification independently of taxonomic ID. Then, to simulate a scenario where pollen identity is unknown, we ran the analysis with the comprehensive database. The databases are referred to as wide (all available taxa) and restricted (mixture taxa only). The taxonomic identification using Kraken and Bracken was repeated 12 times for each of the databases (*matK* restricted and wide, RefSeq restricted and wide) at species, genus and family levels.

## 3 Analysis

Linear regression was used to assess the correlation between the proportions of input pollen weight and resulting sequences. To determine if taxon rarity in the sample affected taxon detection, we used binomial mixed effect models at each taxonomic level, with starting pollen weight proportion as the predictor variable, and a binomial response for detection success or failure. Mix ID was set as a random fixed effect. All modelling was done in RStudio (RStudio Team 2020) using the lme4 package (Bates et al. 2015).

## 4 Results

After sequencing, we retrieved a total of 38,165,440 raw sequencing reads, with an average of 397,557 reads per sample. After filtering, 11,155,855 sequences were retained, an average of 116,207 reads per sample, and 27,009,585 reads were discarded, of which an average of 234,035 sequences per sample were PCR duplicates.

Sample M2a had fewer than 600 sequencing reads remaining after filtering, and was excluded from interpretation as it was likely affected by a technical error. Of the four blanks, only one retained any reads after the quality filtering steps were carried out.

### 4.1 MatK Database

Some challenges were encountered using the *matK* database, particularly the taxonomic ID at the species level, and notable quantification differences compared to the RefSeq database results (below).

#### 4.1.1 Taxonomic Identification

At the species level and using the wide *matK* database for taxonomic assignment, *E. baxteri* was not detected in any of the 42 samples from the 14 mixtures. *Eucalyptus* was detected in all samples at the genus level, apart from the blank. *Arctotheca calendula* was detected in five samples at the species and genus levels. These five samples, plus a sixth with failed detection, were from mixes M13 and M14, which had starting proportions of pollen greater than 0.799. No samples had positive detection of *A. calendula* in mixes with lower starting proportions. *Prunus dulcis* had the highest detection success, being found in all samples except the blank. *Prunus* was detected in every sample at the genus level, including the blank. At the family level, all three taxa (*Myrtaceae*, *Asteraceae* and *Rosaceae*) were detected in every sample except the blank, where no *Myrtaceae* was detected (Figure 3; Table S2).

#### 4.1.2 False Positives

False positives occurred when taxa that were not present in the sample were detected (Table S3). The percentage of false positive sequencing reads was 64.1% using the wide *matK* database at the species level (Figure 4C), 52.3% at the genus level (Figure 4E), and at the family level there was a 10.7% false positive rate (Figure 4G).

#### 4.1.3 Quantification

The relationship between input pollen proportion and proportion of reads was generally highly correlated (*R* ^2^ = 0.62–0.99). *Eucalyptus baxteri* was undetected at the species level, so a correlation could not be calculated. At the genus level, *R* ^2^ = 0.96, but the proportion of reads fell far below the desired 1:1 input to output ratio. At the family level, *R* ^2^ = 0.99, and the proportion of reads detected trended closer to the 1:1 ratio, although they remained below the desired level (Figure 4H). *Arctotheca calendula* had the same relationship between input pollen and output reads at the species and genus levels, which was below the plot threshold, and had the lowest *R* ^2^ value (0.62) for both taxonomic levels. At the family level, *A. calendula* was similarly correlated as *E. baxteri*, with *R* ^2^ = 0.97, and a trend along but consistently below the 1:1 ratio of input pollen to output sequences (Figure 4H). *Prunus dulcis* had a very similar relationship between input pollen and output sequence at each taxonomic level (Figure 4D,F,H), with high *R* ^2^ values (species *R* ^2^ = 0.75, genus and family *R* ^2^ = 0.98). However, the ratio of sequences to starting pollen proportions was positively biassed in comparison to the desired 1:1 ratio in each scenario, and the deviation increased with decreasing taxonomic resolution (Figure 4D,F,H).

#### 4.1.4 Quantification—Restricted Database

The restricted *matK* database (containing only the three taxa used to make mixtures) did not result in any false positives (Figure 4A). The proportion of sequences versus input pollen was linear and highly correlated for all taxa (*R* ^2^ = 0.97–0.99; Figure 4B). The same higher than expected proportion of sequences for *P. dulcis* was seen, but *E. baxteri* and particularly *A. calendula* sequence proportions were much closer to the expected 1:1 ratio (Figure 4B).

### 4.2 RefSeq Database

Using the comprehensive RefSeq database, relatively good results were achieved for taxonomic identification across multiple taxonomic levels, but the quantification results were mixed, showing varying degrees of correlation and accuracy.

#### 4.2.1 Taxonomic Identification

Using the comprehensive RefSeq database, *A. calendula* was detected in the same 31 samples from 10 mixtures at species and genus levels, as it was with the *matK* database. *Eucalyptus baxteri* was found in 6 samples from 3 mixtures at the species level, and at the genus level, it was detected in every sample. At the family level, all three taxa were detected in every sample. *Prunus dulcis* was detected in the blank at all three taxonomic levels, *A. calendula* was detected only at the family level, and *E. baxteri* was not detected at all. This was the same for *matK* except for *P. dulcis* detection at the species level.

#### 4.2.2 False Positives

The percentage of false positive sequencing reads was 72.5% using the wide RefSeq database at the species level (Figure 5C), 47.4% at the genus level (Figure 5E), and a 9.6% false positive ID rate at the family level (Figure 5G).

#### 4.2.3 Quantification

*Eucalyptus baxteri, A. calendula* and *Arctotheca* (at genus level) were detected, but at levels too low to be plotted (Figure 5C,D; Table S3). *Prunus dulcis* sequence proportions were strongly correlated with input pollen proportions (*R* ^2^ = 0.97) and closely tracked the 1:1 ratio until the input pollen proportions reached 0.5, beyond which sequences occurred below the expected level (Figure 5D). *Eucalyptus* ( *E. baxteri* ) was found at approximately half the expected proportion (Figure 5E) but was strongly correlated with input pollen proportion (*R* ^2^ = 0.98). *Prunus* had slightly higher sequence proportions than expected (Figure 5F) and was less linear (*R* ^2^ = 0.9) with a similar flattening of the curve above 0.5 pollen proportion, similar to *P. dulcis* at species level. At family level, all three taxa showed strong correlations between input pollen and sequence proportions (*R* ^2^ = 0.81–1) and plotted along the 1:1 ratio, although *Rosaceae* ( *P. dulcis* ) had the least linearity, as for genus and species (*R* ^2^ = 0.81; Figure 5H). *Myrtaceae* ( *E. baxteri* ) sequence proportions were at expected levels overall, and *Asteraceae* ( *A. calendula* ) and *Rosaceae* were below and above expected levels respectively (Figure 5G).

#### 4.2.4 Quantification—Restricted Database

The restricted RefSeq database (containing only the three taxa used in the mixtures) also did not result in any false positives. The proportion of output sequences versus input pollen was strongly linear for all taxa (*R* ^2^ = 0.96 and 0.97). *Eucalyptus baxteri* and *P. dulcis* points showed more scatter on the plot than for *matK* for samples with less than 0.25 starting pollen proportion. *Arctotheca calendula* had close to zero output sequence reads, and the other two taxa had higher than expected proportions (Figure 5B). Overall, *E. baxteri* had approximately expected read quantities, but *A. calendula* had much lower, and *P. dulcis* much higher than expected read proportions (Figure 5A).

### 4.3 Sample Rarity

The detection of taxa was successful regardless of the starting proportion of pollen in the mix. Starting pollen proportions did not have a significant effect on the detection, using either barcode database for assignment, at any taxonomic level (species, genus or family). Taxon detection versus input pollen proportion was tested in 24 combinations using the four reference databases. In nine cases, the taxon was detected at every pollen input level (every sample), so it was not possible to model (Table 1).

## 5 Discussion

We used hybrid capture to metabarcode artificial pollen mixtures and evaluated the efficacy of taxon ID and quantification of sequence proportions relative to the pollen mixture proportions. We constructed reference databases using Kraken2 and publicly available references from NCBI. We found that the ID of taxa within the pollen mixture provided by a single barcode did not always have resolution to species or genus level. The RefSeq chloroplast database yielded better qualitative results at these taxonomic levels, but its taxon coverage was limited (relative to the species used here). At family level, both databases yielded equally good qualitative results, but the RefSeq database performed better quantitatively. This pattern was not mirrored when using restricted databases containing only the mixture species, probably because *A. calendula* did not have a RefSeq chloroplast genome, and hence it performed better in the wide database containing other *Asteraceae* at Family level. Nevertheless, this hybrid capture method and bioinformatic pipeline performed well in identifying taxa at higher taxonomic levels and found close to a 1:1 ratio of input pollen to output sequences depending on the database used.

Importantly, low species‐level detection and high false positive rates are common challenges across plant DNA metabarcoding studies, regardless of the molecular method or bioinformatics pipeline used (Bell et al. 2019; Lang et al. 2019; Tommasi et al. 2021). These issues arise from limitations in reference databases, low barcode resolution and the inherent difficulty of distinguishing closely related plant taxa using short DNA fragments. Our results are consistent with these broader trends and underscore the persistent challenges in achieving species‐level accuracy in plant metabarcoding. We discuss the implications and current limitations to the method in more detail below.

### 5.1 Taxon Identification

#### 5.1.1 MatK Database

At species level the *matK* database resulted in high levels of false negatives. This was unsurprising as the two standard plant barcodes recommended by CBOL for plant ID can discriminate only approximately 70% of plant species, plus there could have been additional reductions in the resolution since this figure relates to longer barcode sequences, rather than the short fragments generated here. Additionally, species within the *Myrtaceae* (Rivera‐Jiménez et al. 2017) and *Asteraceae* (Arstingstall et al. 2021; Gao et al. 2010) families, (two of the three taxa used here) can be difficult to ID, due to high genetic similarity and shared genetic sequences that complicate differentiation at the species level. One of the reasons could be high chloroplast similarity in not so closely related *Eucalyptus* species (Bayly et al. 2013), which can make barcoding difficult. In this study, *Eucalyptus* may have been difficult to identify at the species level because it had the most related taxa present in the database.

*Prunus dulcis* was readily identified at every taxonomic level, while *Eucalyptus baxteri* was more readily detected at genus level *(Eucalyptus)*, and *Arctotheca calendula* was only readily detected at family level *(Asteraceae)*. In the last case, however, there were no other species of *Arctotheca* in the database (there are only 4–5 accepted species in total), which meant that when the reads did not match the *matK* barcode, the closest matches were more distantly related species, contributing to the high false positive rate at genus level. Since there were many other *Prunus* and *Eucalyptus* species present in the database, *P. dulcis* and *E. baxteri* reads had many more closely related options to match to if the sequence did not match correctly, resulting in more accurate genus level IDs. In early analysis exploration with a database containing only one species per genus, the results yielded were poorer, with more false negatives at genus and family levels. This could occur because the hybrid capture method does not extract the entire barcode, so potentially important parts are missing, and the read matches a different reference. This indicates that it could be important to have closely related species and some ‘redundancy’ in databases to achieve more accurate genus (if not species) level ID.

#### 5.1.2 Refseq Database

Except for *P. dulcis* , which was identified in every sample using the RefSeq database, we had less difficulty identifying the other taxa in the samples compared with the *matK* results. Unlike with *matK, E. baxteri* was identified in some samples at the species level, and *Eucalyptus* was readily identified at the genus level. At the species level, the RefSeq database resulted in more false positives than the *matK* database results, but there were fewer false negatives as well. For results from both databases, the high false positive rate could be attributed to the Illumina sequencing, which is very sensitive and can easily pick up contamination. However, most are likely explained by the misidentification of sequences that came from the true positive species since the false positive rate drops off at the higher taxonomic levels (although still not zero at the family level).

*Arctotheca calendula* had a poorer representation in the RefSeq database. It did not have a publicly available chloroplast reference at the time of database curation, and the database also did not contain other *Arctotheca* species. Instead, the 15 chloroplast sequences available at the time of this study were added to the database (see methods). This most likely led to the much lower‐than‐expected abundance of *A. calendula* using the restricted database. With only the 15 gene regions *A. calendula* reads could possibly hit, versus the entire chloroplast genome for the other two taxa, many of the *A. calendula* sequences which did not match the 15 reference regions well could have matched to regions of the complete chloroplast references for the other taxa and increased the quantity of reads to those. However, at the family level, and with the wide RefSeq database, the proportion of *A. calendula* was closer to expected levels, since with other *Asteraceae* in the database there was more redundancy, and *A. calendula* could match to other more closely related taxa. Again, this suggests that in cases where databases are missing necessary taxa, it is useful to have references of closely related taxa that can provide genus level IDs.

### 5.2 Sample Rarity

There was no relationship between pollen input proportion and detection rate. This result was also found by Bell et al. (2019), who additionally tested the influence of other taxa on identification. In both this study and ours, there appears to be a greater influence of taxon identity than rarity on detection.

### 5.3 Comparison of Single Barcode vs. Whole Chloroplast Database

The design of the hybrid capture baits made the RefSeq database more suitable for qualitative assessment for two main reasons. The first is that more sequences/reads were utilised (*matK* is only one of 19 loci targeted by baits). The *matK* database assigned approximately 1.5% to 3% of reads per sample to a reference, which was unsurprising given the other loci sequenced, but between 85% and 96% of reads assigned to the RefSeq database, resulting in more data being utilised. The second benefit is the potential utilisation of any overhang (resulting from the random fragmentation). This is in contrast to a single barcode database such as the *matK* database used here, where overhangs may prevent sequences from being assigned if the number of nucleotide mismatches exceeds the set threshold.

### 5.4 Quantification

A restricted database only containing the mixture taxa led to linear and highly correlated quantifications of taxon proportions for the *matK* database results, although there appeared to be taxon‐specific biases (these were present in all instances for both databases used). The RefSeq results, which closely followed the expected 1:1 ratio at the family level, were less accurate using the restricted database. This was because the same factors that influenced qualitative success also impacted the quantification of relative taxon proportions. The greatest deviation from the expected ratio was *A. calendula* using the RefSeq database, likely because a whole chloroplast reference was not available for *A. calendula* , thus the sequences were less readily identified and were underestimated. It is evident from this that it is important wherever possible to have equivalent reference sequences for quantitative accuracy, even though the taxon was identified in many of the samples. The most readily identified species ( *P. dulcis* ) had overabundant sequence reads. We expected that there would be a systematic bias arising from the different sizes of the pollen taxa. *Prunus dulcis* was at least twice as large as the other two species, leading to the assumption that fewer pollen grains would be present in a sample of the same weight. Since angiosperm pollen grains have the same number of cells, if each taxon also had the same number of plastids per cell, then we would have expected it to have a lower proportion of sequences than the other two taxa. However, this assumption was not met, and *P. dulcis* was overabundant in all samples, rather than the reverse. This most likely occurred due to two reasons: the assumption about relatively equal numbers of plastids was not true, or the readiness of identification led it to be overestimated. The number of plastids and genome copy number of chloroplasts can vary greatly, from few to hundreds, between different species and tissue types, and tissue age (Morley and Nielsen 2016). While the tissue types were the same in this study, it is likely the species had different numbers of chloroplasts and chloroplast genome copy numbers accounting for some quantitative biases. There may also have been biases stemming from laboratory processes where DNA extraction or sequencing steps might have favoured one taxon over the others.

PCR duplicates were high in this study, accounting for approximately 2/3 of the total reads, but they were readily identified and removed bioinformatically. This level of duplication was expected in our workflow, where the removal of most non‐target DNA would result in very low post‐enrichment DNA quantities. To ensure sufficient material for sequencing, we used an increased number of PCR cycles during library preparation. We recommend future studies aim for 2–5 million reads per sample, or 500,000 to 1 million reads for chloroplast‐targeted data, to retain sufficient unique reads following duplicate removal.

### 5.5 Comparison to Other Studies

Compared to other studies, the hybrid capture method of our study provides weaker qualitative results, whereas our quantitative results are equal or better. All studies considered had highly accurate qualitative results, although the reference databases used and their breadth varied.

Our study had accurate identifications at the family level, but at the species level we only identified all species correctly in some samples using the RefSeq database. We had high levels of false positives for all species, which is a common issue encountered by many biodiversity studies using high‐throughput sequencing for taxon ID (Garrido‐Sanz et al. 2022; Tommasi et al. 2021). Tommasi et al. (2021) also highlighted that different filtering strategies, such as varying cut‐off thresholds, can impact species detection and introduce biases, emphasizing the importance of careful threshold selection. Our results are similar to those of the study by Bell et al. (2021), who used a whole nuclear genome RefSeq database containing publicly available angiosperm species, and found their WGS method to be almost 100% accurate in identifying the species within their pollen mixtures, albeit also with high levels of false positives. High levels of false positives could also be attributed to using a confidence score of zero, which is the default in Kraken2.

In contrast to Bell et al. (2021), we had more highly correlated DNA sequencing and pollen input proportions (*R* ^2^ = 0.72–1 for all taxa at all taxonomic levels), while they found an increasing correlation of *R* ^2^ = 0.60 and *R* ^2^ = 0.62 for species and genus levels. The amplicon metabarcoding used by Bell et al. (2019) found largely accurate taxonomic identifications, but only weakly correlated read proportions with *rbcL* and ITS2 barcodes. The study also found that some taxa were more readily detected, as we found with *P. dulcis* . Similar to our comparison between a *matK* and RefSeq database, Bell et al. (2021) found more taxa could be accurately identified at both species and genus levels using a RefSeq database compared to *rbcL* and ITS2 amplicon sequencing (from Bell et al. (2019)).

The study using RevMet by Peel et al. (2019) reliably identified plants in mixed‐species samples using their custom database containing 54 species at proportions of ≥ 1%, with ‘few’ false positives and negatives. However, the method was only able to quantify high and low abundance levels of taxa. Lang et al. (2019) also found accurate qualitative results, with a 100% accurate identification rate in all samples, at levels as low as 0.2% of the total mixture, using a shotgun sequencing approach. However, their database contained only the species used in their mixtures. Comparatively, our study (although using fewer species) also had a 100% accurate identification rate of taxa in the samples using the database only containing those samples. The study found significant and high correlated proportions of sequencing reads with pollen count proportions (*R* ^2^ = 86.7%), on par with our quantitative results.

### 5.6 Database Selection and Limitations

A comprehensive discussion detailing the current limitations of database availability exists in Bell et al. (2021) under the section ‘4.3 Present feasibility of WGS and future research direction’. The main points are that the availability of whole genome or plastid references required for the WGS method used in their paper (and for the RefSeq database used here) are far below that of the number of ITS2 and *rbcL* sequences available. Further, without many upgrades to currently available sequences, this method will remain limited, and researchers may be forced to create their own references which is time‐consuming and costly. Although this problem is lessening as more reference data continues to be generated. A workaround may be a bioinformatical method for combining data from multiple barcodes into a single analysis, which could utilise the vast quantity of single barcode references already available.

## 6 Applications and Conclusion

Our results demonstrated that a hybrid capture approach with high throughput sequencing is a promising and effective approach for identifying pollen mixes. The strength of using hybrid capture lies in the ability to target multiple genomic regions, potentially utilising more informative loci without prior knowledge about the target taxa. Yet, it remains that there is still no applicable method to combine multiple barcodes in a single analysis, so using a RefSeq chloroplast library generated better results than a single *matK* barcode library. However, there are far fewer plastid sequences available compared with barcode sequences, and missing taxa in the database leads to issues with downstream quantification. Conversely, when the taxa present were known and the database restricted to just those present, the *matK* barcode library resulted in relatively accurate and highly correlated sequence proportions compared with input pollen proportions. This method could be applied to pollinator‐collected pollen samples, but care should be taken with reference choice and database curation, particularly when extracting quantitative information.

## Author Contributions

**D. Kireta:** conceptualization (lead), data curation (lead), formal analysis (lead), investigation (lead), methodology (equal), project administration (equal), visualization (lead), writing – original draft (lead), writing – review and editing (lead). conceptualization (supporting), methodology (equal), project administration (equal), supervision (equal), writing – review and editing (supporting). **K.‐J. van Dijk:** conceptualization (supporting), methodology (equal), project administration (equal), supervision (equal), writing – review and editing (supporting). **S. Crotty:** formal analysis (supporting), investigation (supporting), visualization (equal). **A. Malik:** data curation (equal), investigation (supporting), methodology (supporting), writing – review and editing (supporting). **K. Bell:** investigation (supporting), methodology (supporting), writing – review and editing (supporting). **K. Hogendoorn:** conceptualization (equal), funding acquisition (equal), investigation (supporting), methodology (supporting), project administration (equal), resources (supporting), supervision (supporting), writing – original draft (supporting), writing – review and editing (supporting). **A. J. Lowe:** conceptualization (supporting), funding acquisition (lead), methodology (supporting), project administration (equal), resources (lead), supervision (equal), writing – original draft (supporting), writing – review and editing (supporting).

## Disclosure

Benefit‐sharing: Benefits from this research accrue from the sharing of our data and results on public databases as described above.

## Conflicts of Interest

The authors declare no conflicts of interest.

## Supporting information


Tables S1–S3.

## Data Availability

The data underlying this study have been deposited in Dryad and are available at the following URL: https://doi.org/10.5061/dryad.73n5tb37z.
