# Supplementary material for: A Novel Approach for Pollen Identification and Quantification Using Hybrid Capture‐Based DNA Metabarcoding
Source: Ecol Evol. 2025 Apr 23;15(4):e71311. doi: 10.1002/ece3.71311 (PMC12017898; doi:10.1002/ece3.71311)
Supplement: Supplementary file 1 — Tables S1–S3. [file ECE3-15-e71311-s001.docx]

# Supplementary information:

**A novel approach for pollen identification and quantification using hybrid capture-based DNA metabarcoding**

**Supplementary Table 1:** Initial proportions of three species of pollen in artificial mixes with three replicate samples (Samp.) per mix (M). Species used were *Eucalyptus baxteri* (E, green), *Arctotheca calendula* (A, orange), and *Prunus dulcis* (P, purple).

|  | Proportion based on weight (mg) | | | MatK wide Sp | | | MatK wide Gen | | | MatK wide Fam | | | RefSeq wide Sp | | | RefSeq wide Gen | | | RefSeq wide Fam | | |
| --- | --- | --- | --- | --- | --- | --- | --- | --- | --- | --- | --- | --- | --- | --- | --- | --- | --- | --- | --- | --- | --- |
| **Samp.** | **E prop** | **A prop** | **P prop** | **E** | **A** | **P** | **E** | **A** | **P** | **E** | **A** | **P** | **E** | **A** | **P** | **E** | **A** | **P** | **E** | **A** | **P** |
| **M1a** | 0.012 | 0.050 | 0.937 |  |  |  |  |  |  |  |  |  |  |  |  |  |  |  |  |  |  |
| **M1b** | 0.012 | 0.050 | 0.937 |  |  |  |  |  |  |  |  |  |  |  |  |  |  |  |  |  |  |
| **M1c** | 0.012 | 0.050 | 0.937 |  |  |  |  |  |  |  |  |  |  |  |  |  |  |  |  |  |  |
| **M2a** | 0.054 | 0.099 | 0.847 |  |  |  |  |  |  |  |  |  |  |  |  |  |  |  |  |  |  |
| **M2b** | 0.054 | 0.099 | 0.847 |  |  |  |  |  |  |  |  |  |  |  |  |  |  |  |  |  |  |
| **M2c** | 0.054 | 0.099 | 0.847 |  |  |  |  |  |  |  |  |  |  |  |  |  |  |  |  |  |  |
| **M3a** | 0.103 | 0.201 | 0.696 |  |  |  |  |  |  |  |  |  |  |  |  |  |  |  |  |  |  |
| **M3b** | 0.103 | 0.201 | 0.696 |  |  |  |  |  |  |  |  |  |  |  |  |  |  |  |  |  |  |
| **M3c** | 0.103 | 0.201 | 0.696 |  |  |  |  |  |  |  |  |  |  |  |  |  |  |  |  |  |  |
| **M4a** | 0.200 | 0.302 | 0.497 |  |  |  |  |  |  |  |  |  |  |  |  |  |  |  |  |  |  |
| **M4b** | 0.200 | 0.302 | 0.497 |  |  |  |  |  |  |  |  |  |  |  |  |  |  |  |  |  |  |
| **M4c** | 0.200 | 0.302 | 0.497 |  |  |  |  |  |  |  |  |  |  |  |  |  |  |  |  |  |  |
| **M5a** | 0.335 | 0.336 | 0.329 |  |  |  |  |  |  |  |  |  |  |  |  |  |  |  |  |  |  |
| **M5b** | 0.335 | 0.336 | 0.329 |  |  |  |  |  |  |  |  |  |  |  |  |  |  |  |  |  |  |
| **M5c** | 0.335 | 0.336 | 0.329 |  |  |  |  |  |  |  |  |  |  |  |  |  |  |  |  |  |  |
| **M6a** | 0.451 | 0.349 | 0.200 |  |  |  |  |  |  |  |  |  |  |  |  |  |  |  |  |  |  |
| **M6b** | 0.451 | 0.349 | 0.200 |  |  |  |  |  |  |  |  |  |  |  |  |  |  |  |  |  |  |
| **M6c** | 0.451 | 0.349 | 0.200 |  |  |  |  |  |  |  |  |  |  |  |  |  |  |  |  |  |  |
| **M7a** | 0.699 | 0.200 | 0.101 |  |  |  |  |  |  |  |  |  |  |  |  |  |  |  |  |  |  |
| **M7b** | 0.699 | 0.200 | 0.101 |  |  |  |  |  |  |  |  |  |  |  |  |  |  |  |  |  |  |
| **M7c** | 0.699 | 0.200 | 0.101 |  |  |  |  |  |  |  |  |  |  |  |  |  |  |  |  |  |  |
| **M8a** | 0.798 | 0.152 | 0.050 |  |  |  |  |  |  |  |  |  |  |  |  |  |  |  |  |  |  |
| **M8b** | 0.798 | 0.152 | 0.050 |  |  |  |  |  |  |  |  |  |  |  |  |  |  |  |  |  |  |
| **M8c** | 0.798 | 0.152 | 0.050 |  |  |  |  |  |  |  |  |  |  |  |  |  |  |  |  |  |  |
| **M9a** | 0.939 | 0.050 | 0.011 |  |  |  |  |  |  |  |  |  |  |  |  |  |  |  |  |  |  |
| **M9b** | 0.939 | 0.050 | 0.011 |  |  |  |  |  |  |  |  |  |  |  |  |  |  |  |  |  |  |
| **M9c** | 0.939 | 0.050 | 0.011 |  |  |  |  |  |  |  |  |  |  |  |  |  |  |  |  |  |  |
| **M10a** | 0.539 | 0.009 | 0.452 |  |  |  |  |  |  |  |  |  |  |  |  |  |  |  |  |  |  |
| **M10b** | 0.539 | 0.009 | 0.452 |  |  |  |  |  |  |  |  |  |  |  |  |  |  |  |  |  |  |
| **M10c** | 0.539 | 0.009 | 0.452 |  |  |  |  |  |  |  |  |  |  |  |  |  |  |  |  |  |  |
| **M11a** | 0.350 | 0.450 | 0.200 |  |  |  |  |  |  |  |  |  |  |  |  |  |  |  |  |  |  |
| **M11b** | 0.350 | 0.450 | 0.200 |  |  |  |  |  |  |  |  |  |  |  |  |  |  |  |  |  |  |
| **M11c** | 0.350 | 0.450 | 0.200 |  |  |  |  |  |  |  |  |  |  |  |  |  |  |  |  |  |  |
| **M12a** | 0.151 | 0.700 | 0.149 |  |  |  |  |  |  |  |  |  |  |  |  |  |  |  |  |  |  |
| **M12b** | 0.151 | 0.700 | 0.149 |  |  |  |  |  |  |  |  |  |  |  |  |  |  |  |  |  |  |
| **M12c** | 0.151 | 0.700 | 0.149 |  |  |  |  |  |  |  |  |  |  |  |  |  |  |  |  |  |  |
| **M13a** | 0.100 | 0.799 | 0.101 |  |  |  |  |  |  |  |  |  |  |  |  |  |  |  |  |  |  |
| **M13b** | 0.100 | 0.799 | 0.101 |  |  |  |  |  |  |  |  |  |  |  |  |  |  |  |  |  |  |
| **M13c** | 0.100 | 0.799 | 0.101 |  |  |  |  |  |  |  |  |  |  |  |  |  |  |  |  |  |  |
| **M14a** | 0.032 | 0.939 | 0.030 |  |  |  |  |  |  |  |  |  |  |  |  |  |  |  |  |  |  |
| **M14b** | 0.032 | 0.939 | 0.030 |  |  |  |  |  |  |  |  |  |  |  |  |  |  |  |  |  |  |
| **M14c** | 0.032 | 0.939 | 0.030 |  |  |  |  |  |  |  |  |  |  |  |  |  |  |  |  |  |  |
| **Blank** |  |  |  |  |  |  |  |  |  |  |  |  |  |  |  |  |  |  |  |  |  |

**Supplementary Table 2:** Chloroplast barcodes and sequence details for references for *Arctotheca calendula* downloaded from NCBI in 2022

| Barcode | | Details |
| --- | --- | --- |
| psbZ | | trnS-psbZ intergenic spacer, partial sequence; PsbZ (psbZ) gene, complete cds; psbZ-trnG intergenic spacer and tRNA-Gly (trnG) gene, complete sequence; and trnG-trnfM intergenic spacer, partial sequence |
| psbA | | PsbA (psbA) gene, partial cds; psbA-trnH intergenic spacer, complete sequence; and tRNA-His (trnH) gene, partial sequence |
| ndhF | | NADH dehydrogenase (ndhF) gene, partial cds |
| ndhF | | voucher Trinder-Smith 143 (US) NADH dehydrogenase subunit F (ndhF) gene, partial cds |
| matK | | maturase K (matK) gene, partial cds |
| rbcL | | ribulose-1,5-bisphosphate carboxylase/oxygenase large subunit (rbcL) gene, partial cds |
| matK | | voucher BS0137 maturase K (matK) gene, partial cds |
| rbcL | | voucher BS0137 ribulose-1,5-bisphosphate carboxylase/oxygenase large subunit (rbcL) gene, partial cds |
| trnK | | voucher Trinder-Smith 143 (US) tRNA-Lys (trnK) gene, partial sequence; and maturase K (matK) gene, complete cds |
| ndhF | | NADH dehydrogenase subunit F (ndhF) gene, partial cds; and ndhF-rpl32 intergenic spacer, partial sequence |
| rps16 | | ribosomal protein S16 (rps16) gene, partial sequence |
| trnL | | trnT-trnL intergenic spacer, partial sequence; tRNA-Leu (trnL) gene, complete sequence; and trnL-trnF intergenic spacer, partial sequence |
| trnL | | tRNA-Leu (trnL) gene, partial sequence; trnL-trnF intergenic spacer, complete sequence; and tRNA-Phe (trnF) gene, partial sequence |
| trnL | | voucher Trinder-Smith 143 (US) tRNA-Leu (trnL) gene, partial sequence; trnL-trnF intergenic spacer, complete sequence; and tRNA-Phe (trnF) gene, partial sequence |
| rbcL | chloroplast partial rbcL gene for ribulose bisphosphate carboxylase large subunit, specimen voucher Savolainen V. & Powell M.P. 1801C (NBG) | |

**Supplementary Table 3:** All taxa identified within artificial pollen mixes identified using Kraken, and reads estimated using Bracken. Databases used were MatK and RefSeq, and taxa were identified to Family, Genus or Species level. Results of an assignment using a restricted database only containing the pollen mixture species precedes results of each whole database assignment. Taxa used in the mixture are displayed in bold.

| **MatK restricted db** |  |
| --- | --- |
| Taxon | Mean est. num. reads |
| ***Prunus dulcis*** | **3502.5** |
| ***Eucalyptus baxteri*** | **1923.5** |
| ***Arctotheca calendula*** | **2621.5** |
|  |  |
| **MatK db Family level** |  |
| Taxon | Mean est. num. reads |
| ***Rosaceae*** | **9691.9** |
| *Fabaceae* | 4.4 |
| ***Myrtaceae*** | **3322.9** |
| ***Asteraceae*** | **2978.9** |
| *Goodeniaceae* | 40.1 |
| *Solanaceae* | 1614.5 |
| *Amaranthaceae* | 1.3 |
| *Chenopodiaceae* | 14.0 |
| *Cymodoceaceae* | 1.5 |
| *Linaceae* | 152.3 |
| *Malvaceae* | 36.8 |
| *Euphorbiaceae* | 0.1 |
| *Poaceae* | 3.8 |
| *Asparagaceae* | 1.3 |
| *Acanthaceae* | 0.1 |
| *Hypericaceae* | 0.6 |
| *Fagaceae* | 0.2 |
| *Convolvulaceae* | 1.1 |
| *Caryophyllaceae* | 0.3 |
| *Brassicaceae* | 0.0 |
| *Lythraceae* | 0.6 |
| *Plantaginaceae* | 0.7 |
| *Salicaceae* | 0.0 |
| *Sapindaceae* | 0.1 |
| *Elaeagnaceae* | 0.1 |
| *Casuarinaceae* | 0.1 |
| *Lamiaceae* | 0.0 |
| *Frankeniaceae* | 0.1 |
| *Scrophulariaceae* | 1.1 |
| *Lauraceae* | 0.1 |
| *Pittosporaceae* | 0.1 |
| *Campanulaceae* | 0.4 |
| *Menyanthaceae* | 0.3 |
| *Apiaceae* | 0.1 |
| *Caprifoliaceae* | 0.2 |
| *Garryaceae* | 1.1 |
| *Ericaceae* | 0.2 |
| *Primulaceae* | 0.8 |
| *Rhamnaceae* | 0.1 |
| *Cactaceae* | 0.1 |
| *Crassulaceae* | 0.0 |
| *Amaryllidaceae* | 0.2 |
| *Asphodelaceae* | 0.1 |
|  |  |
| **MatK db Genus level** |  |
| Taxon | Mean est. num. reads |
| ***Prunus*** | **7714.3** |
| *Malus* | 0.6 |
| *Rhaphiolepis* | 3.5 |
| *Spiraea* | 1.6 |
| *Fragaria* | 1947.8 |
| ***Eucalyptus*** | **1178.5** |
| *Hypochaeris* | 910.1 |
| *Picris* | 171.1 |
| *Helminthotheca* | 509.0 |
| *Sonchus* | 397.1 |
| *Reichardia* | 197.0 |
| *Lactuca* | 176.9 |
| *Galinsoga* | 3.5 |
| *Goodenia* | 32.3 |
| *Nicotiana* | 1614.0 |
| *Amphibolis* | 1.4 |
| *Linum* | 152.3 |
| *Crepis* | 28.9 |
| *Taraxacum* | 19.0 |
| *Senecio* | 34.0 |
| *Pallenis* | 4.9 |
| *Pyrus* | 3.4 |
| *Ugni* | 54.4 |
| *Calotis* | 8.1 |
| *Atriplex* | 1.0 |
| *Chenopodium* | 6.2 |
| *Corymbia* | 370.0 |
| *Angophora* | 1572.1 |
| *Beaufortia* | 72.6 |
| *Euryomyrtus* | 32.2 |
| *Sparrmannia* | 36.3 |
| *Pseudognaphalium* | 0.3 |
| *Brachyscome* | 14.6 |
| *Cota* | 5.0 |
| *Cotula* | 2.2 |
| *Flaveria* | 0.4 |
| *Eustrephus* | 0.9 |
| *Leontodon* | 220.2 |
| *Podolepis* | 119.1 |
| *Chrysocephalum* | 17.9 |
| *Avicennia* | 0.1 |
| *Syzygium* | 2.4 |
| *Hypericum* | 0.6 |
| *Acacia* | 1.9 |
| *Melaleuca* | 5.5 |
| *Hysterobaeckea* | 2.7 |
| *Hypocalymma* | 0.9 |
| *Rinzia* | 0.8 |
| *Leptospermum* | 1.7 |
| *Agonis* | 6.7 |
| *Kunzea* | 2.0 |
| *Metrosideros* | 0.7 |
| *Millotia* | 26.3 |
| *Craspedia* | 3.7 |
| *Ozothamnus* | 3.6 |
| *Chondropyxis* | 0.8 |
| *Aster* | 0.9 |
| *Chrysanthemoides* | 3.0 |
| *Helianthus* | 3.3 |
| *Pterocaulon* | 2.0 |
| *Echinops* | 1.2 |
| *Dampiera* | 1.5 |
| *Convolvulus* | 1.1 |
| *Oxybasis* | 0.7 |
| *Rubus* | 0.7 |
| *Urospermum* | 1.3 |
| *Ambrosia* | 1.6 |
| *Verbesina* | 0.9 |
| *Bidens* | 0.7 |
| *Adenostemma* | 1.3 |
| *Gaillardia* | 0.6 |
| *Cynara* | 0.7 |
| *Blitum* | 0.2 |
| *Dysphania* | 0.5 |
| *Pilosella* | 1.0 |
| *Chthonocephalus* | 0.6 |
| *Helichrysum* | 0.7 |
| *Delairea* | 5.1 |
| *Cosmos* | 1.1 |
| *Bellis* | 0.4 |
| *Triodia* | 0.7 |
| *Eleusine* | 0.1 |
| *Leiocarpa* | 1.1 |
| *Medicago* | 0.3 |
| *Callistemon* | 0.3 |
| *Calothamnus* | 0.5 |
| *Myrtus* | 0.5 |
| *Calytrix* | 1.4 |
| *Thryptomene* | 1.9 |
| *Chamelaucium* | 0.6 |
| *Astartea* | 0.7 |
| *Verticordia* | 0.4 |
| *Micromyrtus* | 5.4 |
| *Pericalymma* | 1.5 |
| *Plantago* | 0.6 |
| *Tolpis* | 1.8 |
| *Gnephosis* | 1.8 |
| *Anthemis* | 0.3 |
| *Dimorphotheca* | 2.1 |
| *Punica* | 0.3 |
| *Leptorhynchos* | 1.5 |
| *Actinobole* | 1.0 |
| *Scaevola* | 5.6 |
| *Schoenia* | 0.6 |
| *Centipeda* | 2.7 |
| *Elaeagnus* | 0.1 |
| *Asteridea* | 1.1 |
| *Frankenia* | 0.1 |
| *Erigeron* | 0.5 |
| *Taxandria* | 0.1 |
| *Euphorbia* | 0.0 |
| *Allocasuarina* | 0.1 |
| *Stuartina* | 1.8 |
| *Liatris* | 0.4 |
| *Salicornia* | 1.9 |
| *Myoporum* | 0.5 |
| *Cassytha* | 0.1 |
| *Trifolium* | 0.2 |
| *Chorizema* | 0.1 |
| *Aerva* | 0.3 |
| *Chondrilla* | 0.5 |
| *Tragopogon* | 0.4 |
| *Leucochrysum* | 0.9 |
| *Gratwickia* | 0.2 |
| *Blennospora* | 0.3 |
| *Gamochaeta* | 0.7 |
| *Waitzia* | 1.8 |
| *Angianthus* | 0.9 |
| *Acanthocladium* | 0.5 |
| *Pycnosorus* | 0.3 |
| *Quinetia* | 0.1 |
| *Myriocephalus* | 0.1 |
| *Euryops* | 0.4 |
| *Roldana* | 1.6 |
| *Iva* | 0.3 |
| *Xanthium* | 0.1 |
| *Glossocardia* | 0.2 |
| *Sigesbeckia* | 0.4 |
| *Tagetes* | 0.4 |
| *Elachanthus* | 0.3 |
| *Minuria* | 0.3 |
| *Calendula* | 0.9 |
| *Osteospermum* | 0.3 |
| *Chamaemelum* | 0.3 |
| *Rutidosis* | 0.4 |
| *Centaurea* | 0.7 |
| *Gazania* | 0.3 |
| *Arctotheca* | 0.7 |
| *Trachelium* | 0.2 |
| *Garrya* | 1.4 |
| *Samolus* | 0.1 |
| *Rosa* | 0.1 |
| *Ruppia* | 0.1 |
| *Ceanothus* | 0.1 |
| *Ammobium* | 1.2 |
| *Lemooria* | 0.2 |
| *Zinnia* | 0.2 |
| *Sporobolus* | 2.1 |
| *Vellereophyton* | 0.3 |
| *Lawrencella* | 0.1 |
| *Ageratina* | 0.2 |
| *Conyza* | 1.1 |
| *Cirsium* | 0.1 |
| *Villarsia* | 0.1 |
| *Anagallis* | 0.6 |
| *Lysimachia* | 0.1 |
| *Platylobium* | 0.1 |
| *Tecticornia* | 1.2 |
| *Kali* | 0.3 |
| *Suaeda* | 0.8 |
| *Gomphrena* | 0.4 |
| *Sagina* | 0.1 |
| *Spergularia* | 0.1 |
| *Crassula* | 0.0 |
| *Tulbaghia* | 0.2 |
| *Lythrum* | 0.1 |
| *Beta* | 0.2 |
| *Stypandra* | 0.1 |
|  |  |
| **MatK db Species level** | |
| Taxon | Mean est. num. reads |
| ***Prunus dulcis*** | **6832.2** |
| *Prunus spinosa* | 710.8 |
| *Prunus lusitanica* | 105.4 |
| *Prunus domestica* | 140.0 |
| *Prunus avium* | 39.4 |
| *Prunus cerasus* | 11.0 |
| *Prunus laurocerasus* | 3.7 |
| *Prunus mahaleb* | 5.6 |
| *Prunus persica* | 17.3 |
| *Fragaria x ananassa* | 1996.2 |
| *Eucalyptus haemastoma* | 968.5 |
| *Hypochaeris radicata* | 935.1 |
| *Helminthotheca echioides* | 529.9 |
| *Sonchus arvensis* | 407.6 |
| *Reichardia tingitana* | 204.7 |
| *Galinsoga parviflora* | 3.8 |
| *Goodenia cycloptera* | 32.4 |
| *Nicotiana tabacum* | 1648.4 |
| *Linum strictum* | 155.5 |
| *Lactuca serriola* | 178.9 |
| *Eucalyptus globulus* | 119.4 |
| *Crepis capillaris* | 24.4 |
| *Taraxacum officinale* | 19.1 |
| *Pallenis spinosa* | 5.1 |
| *Spiraea prunifolia* | 0.3 |
| *Ugni molinae* | 55.7 |
| *Calotis scabiosifolia* | 5.7 |
| *Atriplex nummularia* | 0.8 |
| *Angophora floribunda* | 1587.3 |
| *Beaufortia sparsa* | 74.3 |
| *Euryomyrtus ramosissima* | 33.0 |
| *Sparrmannia africana* | 37.1 |
| *Pseudognaphalium luteoalbum* | 0.4 |
| *Cota tinctoria* | 5.3 |
| *Flaveria trinervia* | 0.4 |
| *Prunus armeniaca* | 3.5 |
| *Amphibolis griffithii* | 1.1 |
| *Eustrephus latifolius* | 0.9 |
| *Angophora leiocarpa* | 16.1 |
| *Leontodon saxatilis* | 224.1 |
| *Podolepis canescens* | 126.2 |
| *Avicennia marina* | 0.1 |
| *Hypericum calycinum* | 0.2 |
| *Hypericum perforatum* | 0.1 |
| *Eucalyptus gillenii* | 4.1 |
| *Eucalyptus phenax* | 3.4 |
| *Eucalyptus radiata* | 5.6 |
| *Eucalyptus erythrocorys* | 1.7 |
| *Eucalyptus kingsmillii* | 2.6 |
| *Eucalyptus sturgissiana* | 1.4 |
| *Corymbia intermedia* | 27.5 |
| *Hysterobaeckea behrii* | 2.5 |
| *Agonis flexuosa* | 6.9 |
| *Metrosideros excelsa* | 0.7 |
| *Millotia myosotidifolia* | 28.3 |
| *Chondropyxis halophila* | 0.8 |
| *Aster amellus* | 1.0 |
| *Chrysanthemoides monilifera* | 3.1 |
| *Helianthus tuberosus* | 3.6 |
| *Pterocaulon sphacelatum* | 2.1 |
| *Echinops ritro* | 1.4 |
| *Convolvulus arvensis* | 1.1 |
| *Oxybasis glauca* | 0.7 |
| *Eucalyptus campaspe* | 3.9 |
| *Verbesina encelioides* | 1.0 |
| *Bidens pilosa* | 0.6 |
| *Adenostemma lavenia* | 1.3 |
| *Gaillardia aristata* | 0.6 |
| *Cotula australis* | 1.7 |
| *Cynara cardunculus* | 0.9 |
| *Blitum virgatum* | 0.2 |
| *Corymbia calophylla* | 317.4 |
| *Corymbia trachyphloia* | 0.9 |
| *Leontodon taraxacoides* | 2.5 |
| *Crepis vesicaria* | 2.1 |
| *Pilosella officinarum* | 1.0 |
| *Chthonocephalus pseudevax* | 0.6 |
| *Helichrysum leucopsideum* | 0.8 |
| *Senecio vulgaris* | 16.9 |
| *Delairea odorata* | 5.2 |
| *Bellis perennis* | 0.4 |
| *Triodia basedowii* | 0.4 |
| *Triodia scariosa* | 0.3 |
| *Leiocarpa tomentosa* | 1.2 |
| *Dysphania ambrosioides* | 0.3 |
| *Eucalyptus buprestium* | 1.8 |
| *Eucalyptus annulata* | 2.3 |
| *Eucalyptus mannensis* | 2.6 |
| *Eucalyptus glomerosa* | 4.6 |
| *Eucalyptus fraxinoides* | 3.3 |
| *Eucalyptus platydisca* | 0.7 |
| *Eucalyptus paliformis* | 3.1 |
| *Eucalyptus calcicola* | 0.2 |
| *Eucalyptus botryoides* | 2.1 |
| *Eucalyptus gracilis* | 0.8 |
| *Eucalyptus behriana* | 2.8 |
| *Eucalyptus orbifolia* | 1.4 |
| *Eucalyptus quadrans* | 3.5 |
| *Eucalyptus carnei* | 0.9 |
| *Eucalyptus oleosa* | 1.8 |
| *Eucalyptus flocktoniae* | 1.0 |
| *Eucalyptus brevistylis* | 1.0 |
| *Eucalyptus nitida* | 8.4 |
| *Eucalyptus eremophila* | 0.4 |
| *Eucalyptus gillii* | 1.2 |
| *Eucalyptus coolabah* | 0.4 |
| *Corymbia henryi* | 1.8 |
| *Corymbia leptoloma* | 0.8 |
| *Corymbia aparrerinja* | 2.3 |
| *Angophora costata* | 2.3 |
| *Melaleuca linariifolia* | 0.4 |
| *Melaleuca nesophila* | 2.6 |
| *Calothamnus quadrifidus* | 0.5 |
| *Myrtus communis* | 0.5 |
| *Calytrix tetragona* | 1.4 |
| *Thryptomene saxicola* | 1.3 |
| *Hypocalymma strictum* | 0.6 |
| *Chamelaucium uncinatum* | 0.5 |
| *Astartea fascicularis* | 0.7 |
| *Rinzia ericaea* | 0.1 |
| *Rinzia orientalis* | 0.5 |
| *Verticordia picta* | 0.2 |
| *Micromyrtus ciliata* | 5.6 |
| *Leptospermum epacridoideum* | 1.1 |
| *Leptospermum laevigatum* | 0.4 |
| *Kunzea ericoides* | 1.0 |
| *Kunzea baxteri* | 0.7 |
| *Pericalymma ellipticum* | 1.7 |
| *Syzygium smithii* | 1.1 |
| *Plantago debilis* | 0.5 |
| *Tolpis barbata* | 1.9 |
| *Gnephosis arachnoidea* | 2.0 |
| *Anthemis cotula* | 0.3 |
| *Eucalyptus youngiana* | 0.5 |
| *Eucalyptus gamophylla* | 0.2 |
| *Eucalyptus percostata* | 6.2 |
| *Eucalyptus rugosa* | 0.4 |
| *Eucalyptus laevopinea* | 1.7 |
| *Eucalyptus microcarpa* | 1.0 |
| *Eucalyptus caesia* | 0.1 |
| *Eucalyptus microcorys* | 0.3 |
| *Eucalyptus sparsa* | 0.6 |
| *Eucalyptus steedmanii* | 3.4 |
| *Eucalyptus cneorifolia* | 0.2 |
| *Eucalyptus stricklandii* | 0.6 |
| *Angophora subvelutina* | 0.4 |
| *Melaleuca diosmifolia* | 0.3 |
| *Callistemon citrinus* | 0.2 |
| *Punica granatum* | 0.3 |
| *Leptorhynchos scaber* | 1.4 |
| *Actinobole uliginosum* | 1.1 |
| *Scaevola depauperata* | 4.1 |
| *Eucalyptus crucis* | 1.1 |
| *Eucalyptus macrorhyncha* | 1.2 |
| *Eucalyptus fasciculosa* | 0.4 |
| *Eucalyptus grossa* | 1.1 |
| *Eucalyptus angustissima* | 0.1 |
| *Melaleuca bracteata* | 0.1 |
| *Thryptomene calycina* | 0.4 |
| *Syzygium paniculatum* | 0.7 |
| *Hypochaeris glabra* | 1.1 |
| *Schoenia cassiniana* | 0.7 |
| *Eucalyptus gongylocarpa* | 1.0 |
| *Eucalyptus sargentii* | 0.6 |
| *Eucalyptus guilfoylei* | 8.4 |
| *Eucalyptus pimpiniana* | 0.5 |
| *Eucalyptus calycogona* | 0.4 |
| *Hypocalymma angustifolium* | 0.1 |
| *Elaeagnus pungens* | 0.1 |
| *Asteridea athrixioides* | 1.3 |
| *Frankenia pulverulenta* | 0.1 |
| *Erigeron karvinskianus* | 0.7 |
| *Eucalyptus acies* | 0.6 |
| *Eucalyptus umbra* | 0.3 |
| *Eucalyptus flindersii* | 0.3 |
| *Hysterobaeckea tuberculata* | 0.1 |
| *Chamelaucium drummondii* | 0.1 |
| *Verticordia plumosa* | 0.1 |
| *Taxandria parviceps* | 0.1 |
| *Acacia nematophylla* | 0.2 |
| *Stuartina muelleri* | 2.0 |
| *Liatris spicata* | 0.5 |
| *Brachyscome paludicola* | 0.3 |
| *Brachyscome multifida* | 0.1 |
| *Salicornia pachystachya* | 1.9 |
| *Myoporum montanum* | 0.5 |
| *Trifolium repens* | 0.2 |
| *Chorizema cordatum* | 0.1 |
| *Scaevola aemula* | 1.1 |
| *Chenopodium baccatum* | 2.6 |
| *Chenopodium desertorum* | 1.1 |
| *Aerva javanica* | 0.3 |
| *Picris hieracioides* | 2.1 |
| *Picris angustifolia* | 97.6 |
| *Urospermum dalechampii* | 0.5 |
| *Taraxacum erythrospermum* | 0.1 |
| *Crepis foetida* | 0.4 |
| *Chondrilla juncea* | 0.5 |
| *Tragopogon porrifolius* | 0.4 |
| *Craspedia pleiocephala* | 0.7 |
| *Ozothamnus diosmifolius* | 0.6 |
| *Ozothamnus secundiflorus* | 0.2 |
| *Ozothamnus pholidotus* | 0.1 |
| *Gratwickia monochaeta* | 0.2 |
| *Blennospora drummondii* | 0.3 |
| *Gamochaeta americana* | 0.2 |
| *Waitzia acuminata* | 2.0 |
| *Angianthus preissianus* | 1.1 |
| *Acanthocladium dockeri* | 0.5 |
| *Pycnosorus globosus* | 0.4 |
| *Quinetia urvillei* | 0.1 |
| *Myriocephalus occidentalis* | 0.1 |
| *Euryops virgineus* | 0.4 |
| *Roldana petasitis* | 1.6 |
| *Ambrosia tenuifolia* | 0.8 |
| *Iva axillaris* | 0.3 |
| *Xanthium spinosum* | 0.1 |
| *Cosmos bipinnatus* | 0.8 |
| *Glossocardia bidens* | 0.2 |
| *Sigesbeckia orientalis* | 0.4 |
| *Tagetes erecta* | 0.5 |
| *Brachyscome xanthocarpa* | 0.4 |
| *Brachyscome perpusilla* | 0.1 |
| *Brachyscome melanocarpa* | 9.6 |
| *Calotis lappulacea* | 0.4 |
| *Calotis plumulifera* | 0.5 |
| *Elachanthus pusillus* | 0.3 |
| *Dimorphotheca sinuata* | 1.1 |
| *Calendula officinalis* | 0.8 |
| *Osteospermum clandestinum* | 0.3 |
| *Cotula coronopifolia* | 0.1 |
| *Chamaemelum nobile* | 0.3 |
| *Centipeda elatinoides* | 2.4 |
| *Rutidosis helichrysoides* | 0.4 |
| *Gazania rigens* | 0.3 |
| ***Arctotheca calendula*** | **0.7** |
| *Trachelium caeruleum* | 0.2 |
| *Garrya elliptica* | 1.4 |
| *Samolus valerandi* | 0.1 |
| *Rubus parvifolius* | 0.3 |
| *Acacia elata* | 0.2 |
| *Eucalyptus megacarpa* | 3.8 |
| *Ceanothus thyrsiflorus* | 0.1 |
| *Ammobium alatum* | 1.3 |
| *Lemooria burkittii* | 0.2 |
| *Zinnia elegans* | 0.2 |
| *Sporobolus virginicus* | 2.2 |
| *Lactuca saligna* | 1.7 |
| *Craspedia glauca* | 0.7 |
| *Vellereophyton dealbatum* | 0.3 |
| *Lawrencella davenportii* | 0.1 |
| *Ageratina adenophora* | 0.2 |
| *Brachyscome readeri* | 0.2 |
| *Conyza bonariensis* | 1.2 |
| *Goodenia quasilibera* | 0.1 |
| *Anagallis arvensis* | 0.4 |
| *Anagallis minima* | 0.2 |
| *Lysimachia linum-stellatum* | 0.1 |
| *Platylobium obtusangulum* | 0.1 |
| *Tecticornia indica* | 1.2 |
| *Kali turgidum* | 0.4 |
| *Suaeda aegyptiaca* | 0.6 |
| *Gomphrena celosioides* | 0.4 |
| *Sagina procumbens* | 0.1 |
| *Spergularia rubra* | 0.1 |
| *Tulbaghia violacea* | 0.2 |
| *Lythrum hyssopifolia* | 0.1 |
| *Lythrum junceum* | 0.1 |
| *Suaeda maritima* | 0.2 |
| *Beta vulgaris* | 0.2 |
| *Stypandra glauca* | 0.1 |
|  |  |
| **RefSeq restricted db** |  |
| Taxon | Mean est. num. reads |
| ***Prunus dulcis*** | 202793.9922 |
| ***Eucalyptus baxteri*** | 174666.0365 |
| ***Arctotheca calendula*** | 8659.877582 |
|  |  |
| **RefSeq db Family level** | |
| Taxon | Mean est. num. reads |
| ***Rosaceae*** | **185266.5** |
| *Urticaceae* | 1084.7 |
| *Cannabaceae* | 356.7 |
| *Moraceae* | 355.9 |
| *Rhamnaceae* | 251.1 |
| *Ulmaceae* | 351.9 |
| *Elaeagnaceae* | 87.1 |
| *Barbeyaceae* | 55.0 |
| *Fabaceae* | 4116.2 |
| *Polygalaceae* | 122.5 |
| *Passifloraceae* | 485.4 |
| *Euphorbiaceae* | 385.3 |
| *Salicaceae* | 601.7 |
| *Chrysobalanaceae* | 224.3 |
| *Phyllanthaceae* | 156.2 |
| *Linaceae* | 88.8 |
| *Rhizophoraceae* | 116.3 |
| *Violaceae* | 68.4 |
| *Clusiaceae* | 69.9 |
| *Erythroxylaceae* | 38.2 |
| *Achariaceae* | 49.3 |
| *Malpighiaceae* | 55.8 |
| *Ctenolophonaceae* | 43.8 |
| *Fagaceae* | 713.3 |
| *Betulaceae* | 238.1 |
| *Juglandaceae* | 617.9 |
| *Casuarinaceae* | 37.7 |
| *Myricaceae* | 28.5 |
| *Cucurbitaceae* | 767.9 |
| *Begoniaceae* | 51.2 |
| *Corynocarpaceae* | 38.4 |
| *Zygophyllaceae* | 180.4 |
| *Krameriaceae* | 35.9 |
| *Oxalidaceae* | 145.6 |
| *Elaeocarpaceae* | 43.4 |
| *Celastraceae* | 104.5 |
| ***Myrtaceae*** | **131849.0** |
| *Lythraceae* | 3401.0 |
| *Melastomataceae* | 1397.4 |
| *Onagraceae* | 583.4 |
| *Vochysiaceae* | 894.8 |
| *Combretaceae* | 368.7 |
| *Penaeaceae* | 214.8 |
| *Brassicaceae* | 1453.8 |
| *Capparaceae* | 87.3 |
| *Salvadoraceae* | 41.8 |
| *Resedaceae* | 43.0 |
| *Cleomaceae* | 45.5 |
| *Caricaceae* | 38.2 |
| *Tropaeolaceae* | 30.3 |
| *Pentadiplandraceae* | 16.9 |
| *Akaniaceae* | 18.6 |
| *Moringaceae* | 47.2 |
| *Sapindaceae* | 569.3 |
| *Rutaceae* | 652.8 |
| *Meliaceae* | 144.0 |
| *Anacardiaceae* | 250.0 |
| *Burseraceae* | 137.4 |
| *Nitrariaceae* | 27.0 |
| *Simaroubaceae* | 35.0 |
| *Malvaceae* | 1673.7 |
| *Thymelaeaceae* | 374.4 |
| *Dipterocarpaceae* | 128.6 |
| *Muntingiaceae* | 38.4 |
| *Bixaceae* | 32.0 |
| *Geraniaceae* | 431.5 |
| *Francoaceae* | 42.7 |
| *Staphyleaceae* | 34.3 |
| *Stachyuraceae* | 29.3 |
| *Dipentodontaceae* | 26.5 |
| *Tapisciaceae* | 24.0 |
| *Vitaceae* | 415.8 |
| ***Asteraceae*** | **96270.2** |
| *Menyanthaceae* | 220.6 |
| *Campanulaceae* | 280.4 |
| *Adoxaceae* | 671.0 |
| *Caprifoliaceae* | 342.2 |
| *Apiaceae* | 452.3 |
| *Araliaceae* | 176.2 |
| *Pittosporaceae* | 29.0 |
| *Pennantiaceae* | 9.2 |
| *Aquifoliaceae* | 155.6 |
| *Helwingiaceae* | 22.0 |
| *Cardiopteridaceae* | 92.5 |
| *Orobanchaceae* | 566.9 |
| *Lamiaceae* | 2721.1 |
| *Bignoniaceae* | 296.6 |
| *Oleaceae* | 447.2 |
| *Acanthaceae* | 277.8 |
| *Lentibulariaceae* | 272.7 |
| *Gesneriaceae* | 364.1 |
| *Plantaginaceae* | 167.5 |
| *Verbenaceae* | 32.9 |
| *Scrophulariaceae* | 107.7 |
| *Mazaceae* | 19.2 |
| *Linderniaceae* | 9.3 |
| *Paulowniaceae* | 4.2 |
| *Phrymaceae* | 21.6 |
| *Pedaliaceae* | 1.4 |
| *Gentianaceae* | 233.9 |
| *Rubiaceae* | 355.0 |
| *Apocynaceae* | 252.3 |
| *Loganiaceae* | 50.5 |
| *Convolvulaceae* | 237.2 |
| *Solanaceae* | 235.7 |
| *Eucommiaceae* | 43.8 |
| *Garryaceae* | 27.0 |
| *Boraginaceae* | 51.4 |
| *Lennoaceae* | 17.4 |
| *Icacinaceae* | 106.9 |
| *Primulaceae* | 711.9 |
| *Balsaminaceae* | 123.6 |
| *Ericaceae* | 87.9 |
| *Theaceae* | 473.2 |
| *Styracaceae* | 132.2 |
| *Actinidiaceae* | 66.6 |
| *Sapotaceae* | 85.2 |
| *Ebenaceae* | 102.7 |
| *Lecythidaceae* | 48.9 |
| *Polemoniaceae* | 20.4 |
| *Symplocaceae* | 16.5 |
| *Clethraceae* | 14.2 |
| *Pentaphylacaceae* | 9.7 |
| *Cornaceae* | 164.7 |
| *Hydrangeaceae* | 172.9 |
| *Loasaceae* | 129.1 |
| *Hydrostachyaceae* | 46.0 |
| *Nyssaceae* | 65.2 |
| *Grubbiaceae* | 20.2 |
| *Curtisiaceae* | 6.9 |
| *Chenopodiaceae* | 663.0 |
| *Caryophyllaceae* | 172.3 |
| *Polygonaceae* | 352.7 |
| *Droseraceae* | 81.4 |
| *Cactaceae* | 74.5 |
| *Portulacaceae* | 11.4 |
| *Montiaceae* | 8.8 |
| *Amaranthaceae* | 69.9 |
| *Plumbaginaceae* | 56.8 |
| *Nepenthaceae* | 50.5 |
| *Nyctaginaceae* | 117.6 |
| *Tamaricaceae* | 41.2 |
| *Phytolaccaceae* | 58.4 |
| *Petiveriaceae* | 16.8 |
| *Aizoaceae* | 17.3 |
| *Stegnospermataceae* | 15.3 |
| *Hamamelidaceae* | 455.6 |
| *Saxifragaceae* | 246.8 |
| *Crassulaceae* | 234.1 |
| *Paeoniaceae* | 75.8 |
| *Haloragaceae* | 33.7 |
| *Iteaceae* | 28.4 |
| *Penthoraceae* | 120.6 |
| *Daphniphyllaceae* | 21.4 |
| *Cercidiphyllaceae* | 10.1 |
| *Altingiaceae* | 32.1 |
| *Loranthaceae* | 228.5 |
| *Viscaceae* | 130.8 |
| *Santalaceae* | 55.9 |
| *Schoepfiaceae* | 48.2 |
| *Ximeniaceae* | 46.8 |
| *Erythropalaceae* | 43.1 |
| *Amphorogynaceae* | 30.4 |
| *Cervantesiaceae* | 27.0 |
| *Dilleniaceae* | 61.2 |
| *Orchidaceae* | 432.1 |
| *Asparagaceae* | 91.1 |
| *Amaryllidaceae* | 72.8 |
| *Iridaceae* | 39.5 |
| *Asphodelaceae* | 10.4 |
| *Hyacinthaceae* | 9.6 |
| *Hypoxidaceae* | 3.2 |
| *Poaceae* | 486.0 |
| *Cyperaceae* | 63.3 |
| *Eriocaulaceae* | 11.7 |
| *Typhaceae* | 23.8 |
| *Bromeliaceae* | 4.8 |
| *Flagellariaceae* | 3.8 |
| *Zingiberaceae* | 19.1 |
| *Musaceae* | 13.7 |
| *Commelinaceae* | 7.7 |
| *Haemodoraceae* | 8.2 |
| *Pontederiaceae* | 4.4 |
| *Arecaceae* | 22.2 |
| *Burmanniaceae* | 56.1 |
| *Dioscoreaceae* | 34.8 |
| *Nartheciaceae* | 8.1 |
| *Taccaceae* | 6.8 |
| *Liliaceae* | 55.2 |
| *Melanthiaceae* | 41.3 |
| *Colchicaceae* | 13.0 |
| *Alstroemeriaceae* | 7.4 |
| *Campynemataceae* | 6.3 |
| *Stemonaceae* | 15.4 |
| *Velloziaceae* | 11.2 |
| *Pandanaceae* | 0.7 |
| *Cyclanthaceae* | 2.0 |
| *Hydrocharitaceae* | 186.0 |
| *Cymodoceaceae* | 51.0 |
| *Araceae* | 125.7 |
| *Zosteraceae* | 28.5 |
| *Potamogetonaceae* | 19.5 |
| *Alismataceae* | 16.0 |
| *Aponogetonaceae* | 10.4 |
| *Butomaceae* | 8.1 |
| *Acoraceae* | 6.7 |
| *Ranunculaceae* | 174.1 |
| *Papaveraceae* | 80.3 |
| *Berberidaceae* | 71.5 |
| *Menispermaceae* | 43.7 |
| *Lardizabalaceae* | 18.1 |
| *Circaeasteraceae* | 24.8 |
| *Eupteleaceae* | 5.0 |
| *Lauraceae* | 141.4 |
| *Calycanthaceae* | 7.9 |
| *Aristolochiaceae* | 23.9 |
| *Saururaceae* | 10.7 |
| *Piperaceae* | 7.5 |
| *Annonaceae* | 23.8 |
| *Magnoliaceae* | 22.0 |
| *Winteraceae* | 7.8 |
| *Sabiaceae* | 15.7 |
| *Nelumbonaceae* | 6.2 |
| *Proteaceae* | 15.1 |
| *Platanaceae* | 4.0 |
| *Buxaceae* | 35.9 |
| *Ceratophyllaceae* | 27.1 |
| *Trochodendraceae* | 7.4 |
| *Chloranthaceae* | 8.9 |
| *Schisandraceae* | 1.8 |
| *Nymphaeaceae* | 2.4 |
| *Cabombaceae* | 0.4 |
| *Amborellaceae* | 1.4 |
| *Talinaceae* | 5.0 |
| *Cistaceae* | 3.5 |
| *Joinvilleaceae* | 0.6 |
| *Triuridaceae* | 0.3 |
| *Hydatellaceae* | 0.5 |
| *Carlemanniaceae* | 2.4 |
| *Smilacaceae* | 4.2 |
| *Asteliaceae* | 1.1 |
| *Cytinaceae* | 0.5 |
|  |  |
| **RefSeq db Genus level** | |
| Taxon | Mean est. num. reads |
| ***Prunus*** | **170818.6** |
| *Crataegus* | 5955.6 |
| *Chaenomeles* | 434.6 |
| *Vauquelinia* | 244.8 |
| *Malus* | 1151.7 |
| *Rhaphiolepis* | 360.6 |
| *Kageneckia* | 79.7 |
| *Cotoneaster* | 387.3 |
| *Photinia* | 440.2 |
| *Sorbus* | 116.3 |
| *Amelanchier* | 517.6 |
| *Pyracantha* | 47.5 |
| *Pyrus* | 98.9 |
| *Dichotomanthes* | 37.4 |
| *Pourthiaea* | 55.5 |
| *Osteomeles* | 14.9 |
| *Aronia* | 38.6 |
| *Phippsiomeles* | 21.8 |
| *Cydonia* | 29.9 |
| *Hesperomeles* | 56.8 |
| *Docynia* | 366.2 |
| *Malacomeles* | 24.6 |
| *Spiraea* | 437.3 |
| *Sibiraea* | 143.5 |
| *Pentactina* | 70.4 |
| *Sorbaria* | 570.7 |
| *Neillia* | 310.4 |
| *Gillenia* | 106.0 |
| *Rubus* | 683.5 |
| *Rosa* | 820.5 |
| *Potentilla* | 229.0 |
| *Sibbaldia* | 38.9 |
| *Alchemilla* | 33.2 |
| *Sibbaldianthe* | 33.6 |
| *Fragaria* | 212.5 |
| *Drymocallis* | 9.1 |
| *Comarum* | 10.7 |
| *Potaninia* | 6.7 |
| *Sanguisorba* | 101.3 |
| *Bencomia* | 32.8 |
| *Agrimonia* | 64.6 |
| *Geum* | 160.9 |
| *Pilea* | 632.2 |
| *Elatostema* | 116.9 |
| *Boehmeria* | 156.6 |
| *Urtica* | 44.9 |
| *Poikilospermum* | 41.5 |
| *Pouzolzia* | 38.6 |
| *Procris* | 34.4 |
| *Cecropia* | 13.3 |
| *Debregeasia* | 3.4 |
| *Pteroceltis* | 117.9 |
| *Celtis* | 46.5 |
| *Aphananthe* | 28.4 |
| *Gironniera* | 41.4 |
| *Lozanella* | 32.9 |
| *Cannabis* | 22.2 |
| *Humulus* | 26.2 |
| *Parasponia* | 21.6 |
| *Trema* | 16.3 |
| *Broussonetia* | 56.8 |
| *Ficus* | 92.5 |
| *Artocarpus* | 57.1 |
| *Trophis* | 28.1 |
| *Maclura* | 31.4 |
| *Streblus* | 47.5 |
| *Morus* | 19.8 |
| *Antiaris* | 19.7 |
| *Rhamnus* | 47.9 |
| *Berchemia* | 23.6 |
| *Ventilago* | 47.3 |
| *Ziziphus* | 112.3 |
| *Hovenia* | 17.7 |
| *Ulmus* | 305.6 |
| *Chaetachme* | 16.9 |
| *Zelkova* | 26.9 |
| *Hippophae* | 56.0 |
| *Elaeagnus* | 30.6 |
| *Barbeya* | 55.1 |
| *Lathyrus* | 114.7 |
| *Vicia* | 64.6 |
| *Pisum* | 30.5 |
| *Lens* | 11.0 |
| *Vavilovia* | 9.2 |
| *Trifolium* | 59.3 |
| *Medicago* | 60.0 |
| *Parochetus* | 12.3 |
| *Melilotus* | 7.4 |
| *Galega* | 12.8 |
| *Sphaerophysa* | 59.4 |
| *Oxytropis* | 14.2 |
| *Glycyrrhiza* | 12.9 |
| *Astragalus* | 27.6 |
| *Lessertia* | 5.4 |
| *Tibetia* | 10.9 |
| *Caragana* | 33.2 |
| *Hedysarum* | 19.8 |
| *Onobrychis* | 15.9 |
| *Alhagi* | 12.9 |
| *Cicer* | 11.8 |
| *Lotus* | 19.4 |
| *Securigera* | 30.8 |
| *Sesbania* | 22.9 |
| *Robinia* | 19.5 |
| *Centrosema* | 18.6 |
| *Spatholobus* | 19.3 |
| *Erythrina* | 22.0 |
| *Vigna* | 21.1 |
| *Glycine* | 29.7 |
| *Canavalia* | 30.6 |
| *Mucuna* | 15.9 |
| *Phaseolus* | 24.5 |
| *Hardenbergia* | 10.8 |
| *Pachyrhizus* | 15.7 |
| *Apios* | 11.9 |
| *Lablab* | 6.9 |
| *Haymondia* | 9.2 |
| *Cajanus* | 11.5 |
| *Psophocarpus* | 12.3 |
| *Amphicarpaea* | 12.0 |
| *Kennedia* | 6.4 |
| *Austrosteenisia* | 14.4 |
| *Philenoptera* | 13.2 |
| *Dahlstedtia* | 18.0 |
| *Pongamia* | 13.9 |
| *Aganope* | 19.4 |
| *Millettia* | 11.6 |
| *Derris* | 12.9 |
| *Alysicarpus* | 12.0 |
| *Tadehagi* | 6.3 |
| *Grona* | 10.3 |
| *Lespedeza* | 2.3 |
| *Ohwia* | 4.2 |
| *Phyllodium* | 3.8 |
| *Hanslia* | 2.9 |
| *Campylotropis* | 2.3 |
| *Wisteria* | 5.1 |
| *Nanhaia* | 9.0 |
| *Indigofera* | 17.5 |
| *Cullen* | 5.6 |
| *Dalbergia* | 396.3 |
| *Kotschya* | 24.7 |
| *Stylosanthes* | 24.8 |
| *Arachis* | 145.8 |
| *Pterocarpus* | 49.1 |
| *Sophora* | 617.8 |
| *Ammopiptanthus* | 3.3 |
| *Maackia* | 11.9 |
| *Piptanthus* | 1.3 |
| *Lupinus* | 53.5 |
| *Crotalaria* | 10.1 |
| *Podalyria* | 3.4 |
| *Ormosia* | 30.8 |
| *Cladrastis* | 8.8 |
| *Styphnolobium* | 8.2 |
| *Vachellia* | 624.6 |
| *Acacia* | 73.9 |
| *Senegalia* | 12.7 |
| *Mimosa* | 42.8 |
| *Xylia* | 19.3 |
| *Prosopis* | 55.9 |
| *Dichrostachys* | 6.8 |
| *Stryphnodendron* | 23.8 |
| *Leucaena* | 10.8 |
| *Adenanthera* | 19.8 |
| *Albizia* | 22.6 |
| *Pararchidendron* | 18.4 |
| *Archidendron* | 7.7 |
| *Faidherbia* | 8.6 |
| *Inga* | 6.7 |
| *Balsamocarpon* | 21.4 |
| *Erythrostemon* | 28.7 |
| *Biancaea* | 10.2 |
| *Senna* | 20.5 |
| *Haematoxylum* | 5.8 |
| *Gleditsia* | 7.7 |
| *Colvillea* | 10.5 |
| *Piliostigma* | 23.3 |
| *Tylosema* | 26.8 |
| *Bauhinia* | 18.6 |
| *Barklya* | 11.9 |
| *Schnella* | 13.3 |
| *Adenolobus* | 32.3 |
| *Griffonia* | 17.5 |
| *Cercis* | 8.2 |
| *Daniellia* | 31.8 |
| *Guibourtia* | 16.6 |
| *Saraca* | 25.9 |
| *Crudia* | 29.7 |
| *Schotia* | 15.1 |
| *Afzelia* | 12.5 |
| *Zenia* | 16.5 |
| *Distemonanthus* | 14.5 |
| *Ceratonia* | 43.4 |
| *Polygala* | 97.8 |
| *Salomonia* | 21.0 |
| *Epirixanthes* | 2.8 |
| *Passiflora* | 450.2 |
| *Adenia* | 25.2 |
| *Dilkea* | 7.4 |
| *Vernicia* | 51.4 |
| *Deutzianthus* | 18.7 |
| *Jatropha* | 14.9 |
| *Manihot* | 20.9 |
| *Croton* | 21.9 |
| *Hevea* | 12.1 |
| *Euphorbia* | 129.2 |
| *Balakata* | 21.8 |
| *Mallotus* | 43.0 |
| *Ricinus* | 16.5 |
| *Plukenetia* | 30.7 |
| *Populus* | 301.2 |
| *Salix* | 127.6 |
| *Itoa* | 24.7 |
| *Idesia* | 20.3 |
| *Poliothyrsis* | 12.0 |
| *Dianyuea* | 33.0 |
| *Homalium* | 45.8 |
| *Banara* | 7.3 |
| *Grangeria* | 37.4 |
| *Licania* | 65.5 |
| *Hirtella* | 14.0 |
| *Kostermanthus* | 7.3 |
| *Parinari* | 8.1 |
| *Couepia* | 10.7 |
| *Dactyladenia* | 42.8 |
| *Magnistipula* | 5.3 |
| *Chrysobalanus* | 6.5 |
| *Phyllanthus* | 48.8 |
| *Flueggea* | 8.8 |
| *Breynia* | 8.0 |
| *Glochidion* | 5.9 |
| *Sauropus* | 1.3 |
| *Leptopus* | 45.7 |
| *Baccaurea* | 32.4 |
| *Linum* | 88.9 |
| *Bruguiera* | 50.9 |
| *Kandelia* | 28.0 |
| *Rhizophora* | 20.9 |
| *Pellacalyx* | 15.1 |
| *Viola* | 68.5 |
| *Garcinia* | 70.0 |
| *Erythroxylum* | 38.3 |
| *Hydnocarpus* | 49.4 |
| *Galphimia* | 15.4 |
| *Byrsonima* | 18.1 |
| *Bunchosia* | 10.0 |
| *Ctenolophon* | 43.9 |
| *Fagus* | 72.0 |
| *Quercus* | 441.9 |
| *Castanea* | 59.6 |
| *Trigonobalanus* | 33.9 |
| *Castanopsis* | 100.4 |
| *Alnus* | 66.4 |
| *Corylus* | 25.0 |
| *Carpinus* | 120.0 |
| *Betula* | 2.7 |
| *Ostryopsis* | 6.9 |
| *Carya* | 199.9 |
| *Juglans* | 375.0 |
| *Engelhardia* | 10.7 |
| *Platycarya* | 8.2 |
| *Rhoiptelea* | 15.6 |
| *Pterocarya* | 4.4 |
| *Casuarina* | 37.7 |
| *Morella* | 28.5 |
| *Trichosanthes* | 89.3 |
| *Nothoalsomitra* | 54.9 |
| *Cyclanthera* | 17.7 |
| *Linnaeosicyos* | 18.4 |
| *Hodgsonia* | 29.6 |
| *Sechium* | 25.0 |
| *Gynostemma* | 133.2 |
| *Hemsleya* | 25.0 |
| *Cucumis* | 52.4 |
| *Citrullus* | 37.1 |
| *Benincasa* | 11.6 |
| *Coccinia* | 9.9 |
| *Cyclantheropsis* | 37.7 |
| *Cucurbita* | 36.2 |
| *Cionosicys* | 14.0 |
| *Momordica* | 34.3 |
| *Gerrardanthus* | 21.9 |
| *Dendrosicyos* | 9.8 |
| *Indofevillea* | 14.9 |
| *Herpetospermum* | 10.3 |
| *Thladiantha* | 12.9 |
| *Bryonia* | 12.6 |
| *Siraitia* | 23.7 |
| *Ampelosycios* | 6.4 |
| *Begonia* | 51.2 |
| *Corynocarpus* | 38.5 |
| *Zygophyllum* | 62.3 |
| *Tetraena* | 28.4 |
| *Guaiacum* | 30.3 |
| *Larrea* | 27.3 |
| *Tribulus* | 30.6 |
| *Krameria* | 35.9 |
| *Oxalis* | 118.2 |
| *Averrhoa* | 27.1 |
| *Sloanea* | 7.9 |
| *Elaeocarpus* | 35.3 |
| *Parnassia* | 53.4 |
| *Euonymus* | 22.4 |
| *Salacia* | 14.9 |
| *Maytenus* | 12.6 |
| *Eucalyptus* | 71283.4 |
| *Corymbia* | 13349.1 |
| *Stockwellia* | 1083.0 |
| *Allosyncarpia* | 851.7 |
| *Syzygium* | 2458.4 |
| *Plinia* | 1847.9 |
| *Psidium* | 229.2 |
| *Rhodomyrtus* | 840.9 |
| *Eugenia* | 19.1 |
| *Melaleuca* | 1114.2 |
| *Heteropyxis* | 326.4 |
| *Duabanga* | 1479.4 |
| *Trapa* | 110.8 |
| *Lagerstroemia* | 597.4 |
| *Cuphea* | 48.8 |
| *Lythrum* | 31.7 |
| *Woodfordia* | 893.1 |
| *Sonneratia* | 36.6 |
| *Punica* | 59.4 |
| *Heimia* | 34.7 |
| *Salpinga* | 178.3 |
| *Opisthocentra* | 33.8 |
| *Phyllagathis* | 17.0 |
| *Nepsera* | 44.8 |
| *Heterocentron* | 43.4 |
| *Chaetogastra* | 46.5 |
| *Pleroma* | 14.2 |
| *Graffenrieda* | 58.0 |
| *Triolena* | 34.2 |
| *Rhexia* | 49.3 |
| *Microlicia* | 20.4 |
| *Rhynchanthera* | 27.3 |
| *Bertolonia* | 34.6 |
| *Merianthera* | 136.4 |
| *Henriettea* | 22.8 |
| *Blakea* | 26.4 |
| *Allomaieta* | 33.8 |
| *Medinilla* | 20.0 |
| *Memecylon* | 103.4 |
| *Tigridiopalma* | 314.5 |
| *Pterogastra* | 12.7 |
| *Eriocnema* | 35.3 |
| *Ludwigia* | 229.0 |
| *Oenothera* | 218.1 |
| *Chamaenerion* | 86.2 |
| *Epilobium* | 49.1 |
| *Korupodendron* | 151.2 |
| *Erisma* | 74.1 |
| *Callisthene* | 149.2 |
| *Ruizterania* | 119.0 |
| *Qualea* | 59.3 |
| *Vochysia* | 47.2 |
| *Terminalia* | 176.9 |
| *Combretum* | 68.1 |
| *Laguncularia* | 75.5 |
| *Lumnitzera* | 34.8 |
| *Quisqualis* | 10.7 |
| *Saltera* | 214.9 |
| *Solms-laubachia* | 136.8 |
| *Lepidostemon* | 5.7 |
| *Dilophia* | 6.5 |
| *Braya* | 18.2 |
| *Dichasianthus* | 4.9 |
| *Christolea* | 14.1 |
| *Cryptospora* | 4.0 |
| *Tetracme* | 13.0 |
| *Atelanthera* | 90.6 |
| *Neotorularia* | 6.3 |
| *Pycnoplinthus* | 2.8 |
| *Strigosella* | 8.1 |
| *Ricotia* | 60.5 |
| *Biscutella* | 20.8 |
| *Lunaria* | 12.0 |
| *Heldreichia* | 6.1 |
| *Megadenia* | 4.0 |
| *Hemilophia* | 26.6 |
| *Chamira* | 17.6 |
| *Idahoa* | 42.0 |
| *Dipoma* | 11.6 |
| *Asperuginoides* | 6.3 |
| *Ochthodium* | 2.6 |
| *Crambe* | 5.3 |
| *Orychophragmus* | 5.9 |
| *Brassica* | 85.1 |
| *Sinalliaria* | 6.8 |
| *Henophyton* | 3.1 |
| *Cremolobus* | 19.2 |
| *Menonvillea* | 15.5 |
| *Diptychocarpus* | 19.7 |
| *Chorispora* | 11.4 |
| *Heliophila* | 35.7 |
| *Aphragmus* | 33.1 |
| *Draba* | 28.8 |
| *Arabis* | 16.7 |
| *Aethionema* | 32.4 |
| *Moriera* | 11.1 |
| *Cardamine* | 27.9 |
| *Nasturtium* | 5.9 |
| *Iodanthus* | 4.7 |
| *Alyssum* | 10.6 |
| *Aurinia* | 7.4 |
| *Meniocus* | 0.7 |
| *Matthiola* | 60.3 |
| *Lepidium* | 21.7 |
| *Delpinophytum* | 4.0 |
| *Notoceras* | 4.8 |
| *Lobularia* | 2.9 |
| *Eutrema* | 24.8 |
| *Cochlearia* | 10.1 |
| *Mostacillastrum* | 25.6 |
| *Hornungia* | 4.8 |
| *Descurainia* | 6.3 |
| *Arabidopsis* | 15.0 |
| *Capsella* | 1.0 |
| *Asta* | 11.3 |
| *Mancoa* | 6.7 |
| *Macropodium* | 6.9 |
| *Microthlaspi* | 7.4 |
| *Dontostemon* | 4.2 |
| *Ladakiella* | 3.6 |
| *Crucihimalaya* | 8.6 |
| *Megacarpaea* | 8.9 |
| *Hesperis* | 7.7 |
| *Notothlaspi* | 5.5 |
| *Smelowskia* | 2.8 |
| *Iberis* | 3.8 |
| *Myagrum* | 0.9 |
| *Capparis* | 42.5 |
| *Maerua* | 12.3 |
| *Crateva* | 18.4 |
| *Cadaba* | 13.3 |
| *Azima* | 19.1 |
| *Salvadora* | 22.4 |
| *Ochradenus* | 20.3 |
| *Caylusea* | 21.7 |
| *Cleomella* | 22.1 |
| *Tarenaya* | 6.1 |
| *Carica* | 21.9 |
| *Tropaeolum* | 30.4 |
| *Pentadiplandra* | 16.9 |
| *Bretschneidera* | 8.7 |
| *Moringa* | 47.4 |
| *Acer* | 354.6 |
| *Dipteronia* | 21.2 |
| *Handeliodendron* | 20.3 |
| *Aesculus* | 32.2 |
| *Eurycorymbus* | 36.9 |
| *Dodonaea* | 23.0 |
| *Koelreuteria* | 15.7 |
| *Xanthoceras* | 34.5 |
| *Pometia* | 9.8 |
| *Sapindus* | 7.2 |
| *Dimocarpus* | 1.1 |
| *Zanthoxylum* | 272.8 |
| *Casimiroa* | 19.2 |
| *Melicope* | 20.0 |
| *Citrus* | 219.7 |
| *Murraya* | 19.3 |
| *Glycosmis* | 16.2 |
| *Clausena* | 15.5 |
| *Ruta* | 32.7 |
| *Tetradium* | 10.7 |
| *Orixa* | 12.4 |
| *Micromelum* | 8.9 |
| *Azadirachta* | 26.8 |
| *Khaya* | 16.8 |
| *Entandrophragma* | 15.5 |
| *Xylocarpus* | 28.8 |
| *Heynea* | 7.2 |
| *Melia* | 6.4 |
| *Carapa* | 6.9 |
| *Aphanamixis* | 7.5 |
| *Mangifera* | 34.3 |
| *Spondias* | 21.3 |
| *Rhus* | 17.8 |
| *Toxicodendron* | 114.7 |
| *Anacardium* | 16.1 |
| *Astronium* | 5.2 |
| *Sclerocarya* | 8.2 |
| *Pistacia* | 17.4 |
| *Lannea* | 6.3 |
| *Commiphora* | 123.0 |
| *Canarium* | 10.9 |
| *Peganum* | 27.1 |
| *Leitneria* | 23.0 |
| *Ailanthus* | 11.1 |
| *Gossypium* | 1053.9 |
| *Hibiscus* | 21.4 |
| *Sida* | 5.8 |
| *Heritiera* | 70.7 |
| *Firmiana* | 28.3 |
| *Sterculia* | 24.4 |
| *Durio* | 34.4 |
| *Reevesia* | 21.1 |
| *Corchorus* | 25.0 |
| *Grewia* | 30.8 |
| *Colona* | 6.9 |
| *Pterospermum* | 7.3 |
| *Craigia* | 19.0 |
| *Wikstroemia* | 72.6 |
| *Daphne* | 189.3 |
| *Gonystylus* | 26.8 |
| *Pimelea* | 36.3 |
| *Aquilaria* | 14.4 |
| *Phaleria* | 10.4 |
| *Edgeworthia* | 19.0 |
| *Diarthron* | 0.6 |
| *Vatica* | 31.4 |
| *Shorea* | 27.9 |
| *Dipterocarpus* | 14.6 |
| *Hopea* | 43.2 |
| *Muntingia* | 38.4 |
| *Bixa* | 32.0 |
| *Pelargonium* | 153.4 |
| *Erodium* | 165.9 |
| *Monsonia* | 53.4 |
| *Hypseocharis* | 32.9 |
| *Geranium* | 24.6 |
| *Viviania* | 42.7 |
| *Euscaphis* | 21.0 |
| *Turpinia* | 10.6 |
| *Stachyurus* | 29.5 |
| *Dipentodon* | 26.5 |
| *Tapiscia* | 24.1 |
| *Vitis* | 363.7 |
| *Ampelopsis* | 40.3 |
| *Tetrastigma* | 10.9 |
| *Hypochaeris* | 68772.9 |
| *Ixeris* | 1216.1 |
| *Taraxacum* | 659.3 |
| *Crepidiastrum* | 770.1 |
| *Lactuca* | 1393.4 |
| *Sonchus* | 1449.5 |
| *Reichardia* | 653.3 |
| *Cichorium* | 275.9 |
| *Bidens* | 1483.8 |
| *Cosmos* | 110.4 |
| *Ambrosia* | 718.2 |
| *Aldama* | 465.6 |
| *Parthenium* | 570.6 |
| *Guizotia* | 340.8 |
| *Galinsoga* | 127.5 |
| *Praxelis* | 325.7 |
| *Mikania* | 35.1 |
| *Ageratina* | 119.6 |
| *Chromolaena* | 75.3 |
| *Marshallia* | 272.0 |
| *Tagetes* | 49.2 |
| *Achyrachaena* | 110.6 |
| *Crossostephium* | 65.5 |
| *Stilpnolepis* | 16.2 |
| *Leucanthemum* | 92.5 |
| *Soliva* | 106.7 |
| *Tanacetum* | 48.4 |
| *Diplostephium* | 1651.6 |
| *Exostigma* | 53.1 |
| *Archibaccharis* | 249.6 |
| *Linochilus* | 5158.7 |
| *Baccharis* | 75.9 |
| *Symphyotrichum* | 23.6 |
| *Aster* | 225.0 |
| *Eschenbachia* | 227.3 |
| *Dendrosenecio* | 340.6 |
| *Farfugium* | 33.0 |
| *Ligularia* | 149.4 |
| *Petasites* | 14.7 |
| *Pluchea* | 78.3 |
| *Saussurea* | 1608.7 |
| *Atractylodes* | 382.8 |
| *Tugarinovia* | 110.6 |
| *Cirsium* | 201.8 |
| *Doniophyton* | 86.5 |
| *Gerbera* | 136.8 |
| *Gymnanthemum* | 131.4 |
| *Nymphoides* | 188.8 |
| *Menyanthes* | 31.4 |
| *Adenophora* | 44.2 |
| *Campanula* | 19.0 |
| *Burmeistera* | 122.1 |
| *Platycodon* | 30.7 |
| *Leptocodon* | 12.8 |
| *Trachelium* | 10.6 |
| *Codonopsis* | 16.4 |
| *Viburnum* | 654.6 |
| *Sambucus* | 14.4 |
| *Lonicera* | 91.1 |
| *Patrinia* | 42.0 |
| *Acanthocalyx* | 10.2 |
| *Triosteum* | 25.6 |
| *Pterocephalus* | 8.5 |
| *Dipsacus* | 19.1 |
| *Nardostachys* | 11.7 |
| *Heptacodium* | 19.0 |
| *Scabiosa* | 11.4 |
| *Valeriana* | 16.5 |
| *Abelia* | 10.8 |
| *Zabelia* | 21.1 |
| *Morina* | 7.5 |
| *Weigela* | 16.8 |
| *Triplostegia* | 8.8 |
| *Angelica* | 62.4 |
| *Peucedanum* | 15.1 |
| *Glehnia* | 9.8 |
| *Heracleum* | 16.5 |
| *Semenovia* | 8.4 |
| *Carum* | 12.9 |
| *Crithmum* | 14.3 |
| *Anethum* | 13.1 |
| *Prangos* | 5.1 |
| *Coriandrum* | 4.8 |
| *Bupleurum* | 114.8 |
| *Ligusticum* | 29.8 |
| *Meeboldia* | 5.3 |
| *Pterygopleurum* | 9.3 |
| *Pternopetalum* | 2.3 |
| *Ferula* | 11.9 |
| *Cuminum* | 6.0 |
| *Anthriscus* | 6.9 |
| *Pleurospermum* | 17.6 |
| *Haplosphaera* | 5.5 |
| *Heteromorpha* | 6.5 |
| *Cicuta* | 2.2 |
| *Changium* | 5.7 |
| *Panax* | 80.0 |
| *Dendropanax* | 17.1 |
| *Hydrocotyle* | 10.5 |
| *Brassaiopsis* | 6.6 |
| *Schefflera* | 14.1 |
| *Aralia* | 20.6 |
| *Pittosporum* | 29.2 |
| *Pennantia* | 9.3 |
| *Ilex* | 156.5 |
| *Helwingia* | 22.1 |
| *Gonocaryum* | 92.7 |
| *Aphyllon* | 82.4 |
| *Orobanche* | 68.8 |
| *Cistanche* | 42.4 |
| *Phelipanche* | 18.2 |
| *Epifagus* | 2.9 |
| *Lathraea* | 65.0 |
| *Euphrasia* | 22.6 |
| *Melampyrum* | 37.1 |
| *Schwalbea* | 29.0 |
| *Siphonostegia* | 13.3 |
| *Phtheirospermum* | 3.8 |
| *Pedicularis* | 130.0 |
| *Triphysaria* | 7.4 |
| *Lindenbergia* | 9.6 |
| *Triaenophora* | 11.4 |
| *Rehmannia* | 11.3 |
| *Salvia* | 61.4 |
| *Nepeta* | 10.6 |
| *Dracocephalum* | 6.3 |
| *Prunella* | 3.4 |
| *Clinopodium* | 3.7 |
| *Coleus* | 3.8 |
| *Lavandula* | 7.8 |
| *Hanceola* | 5.3 |
| *Elsholtzia* | 12.1 |
| *Teucrium* | 47.4 |
| *Caryopteris* | 8.6 |
| *Ajuga* | 5.4 |
| *Clerodendrum* | 351.1 |
| *Pogostemon* | 22.8 |
| *Leucosceptrum* | 10.7 |
| *Eriophyton* | 4.2 |
| *Dasymalla* | 15.4 |
| *Dicrastylis* | 27.8 |
| *Premna* | 15.2 |
| *Gmelina* | 6.5 |
| *Scutellaria* | 13.4 |
| *Holmskioldia* | 3.2 |
| *Vitex* | 8.4 |
| *Congea* | 12.7 |
| *Tectona* | 5.8 |
| *Callicarpa* | 1979.4 |
| *Clerodendranthus* | 2.5 |
| *Adenocalymma* | 126.0 |
| *Amphilophium* | 32.8 |
| *Anemopaegma* | 14.5 |
| *Dolichandra* | 4.6 |
| *Tanaecium* | 9.1 |
| *Neojobertia* | 2.1 |
| *Incarvillea* | 47.4 |
| *Tecomaria* | 6.4 |
| *Dolichandrone* | 17.3 |
| *Spathodea* | 5.7 |
| *Oroxylum* | 12.7 |
| *Catalpa* | 5.6 |
| *Syringa* | 36.3 |
| *Chionanthus* | 63.8 |
| *Ligustrum* | 22.2 |
| *Fraxinus* | 29.1 |
| *Osmanthus* | 93.4 |
| *Olea* | 30.8 |
| *Noronhia* | 23.1 |
| *Jasminum* | 27.2 |
| *Chrysojasminum* | 17.7 |
| *Nyctanthes* | 17.4 |
| *Justicia* | 38.4 |
| *Peristrophe* | 4.8 |
| *Pseuderanthemum* | 5.8 |
| *Clinacanthus* | 3.7 |
| *Echinacanthus* | 61.1 |
| *Strobilanthes* | 10.7 |
| *Aphelandra* | 26.2 |
| *Acanthus* | 77.0 |
| *Blepharis* | 6.3 |
| *Andrographis* | 11.3 |
| *Avicennia* | 16.5 |
| *Utricularia* | 183.5 |
| *Genlisea* | 53.0 |
| *Pinguicula* | 35.8 |
| *Primulina* | 29.7 |
| *Oreocharis* | 23.5 |
| *Petrocodon* | 9.8 |
| *Lysionotus* | 4.7 |
| *Hemiboea* | 2.1 |
| *Paraboea* | 60.5 |
| *Corallodiscus* | 11.5 |
| *Streptocarpus* | 4.6 |
| *Haberlea* | 8.2 |
| *Achimenes* | 198.9 |
| *Veronica* | 6.4 |
| *Veronicastrum* | 12.6 |
| *Lagotis* | 7.0 |
| *Plantago* | 46.0 |
| *Hippuris* | 14.6 |
| *Hemiphragma* | 13.9 |
| *Bacopa* | 62.7 |
| *Lippia* | 10.3 |
| *Duranta* | 17.2 |
| *Scrophularia* | 17.1 |
| *Verbascum* | 6.0 |
| *Wightia* | 71.5 |
| *Buddleja* | 11.7 |
| *Mazus* | 15.4 |
| *Torenia* | 9.3 |
| *Paulownia* | 4.2 |
| *Erythranthe* | 8.2 |
| *Phryma* | 6.4 |
| *Sesamum* | 1.4 |
| *Gentiana* | 73.4 |
| *Kuepferia* | 24.0 |
| *Tripterospermum* | 9.8 |
| *Swertia* | 51.4 |
| *Comastoma* | 8.8 |
| *Lomatogonium* | 3.9 |
| *Exacum* | 29.8 |
| *Cyrtophyllum* | 10.7 |
| *Leptodermis* | 29.4 |
| *Paederia* | 12.2 |
| *Gynochthodes* | 63.2 |
| *Morinda* | 19.4 |
| *Oldenlandia* | 120.4 |
| *Rubia* | 9.7 |
| *Ophiorrhiza* | 16.8 |
| *Gardenia* | 10.8 |
| *Coffea* | 9.8 |
| *Scyphiphora* | 14.1 |
| *Emmenopterys* | 7.9 |
| *Antirhea* | 8.1 |
| *Asclepias* | 12.1 |
| *Calotropis* | 0.6 |
| *Cynanchum* | 11.4 |
| *Pentasachme* | 46.7 |
| *Hoya* | 10.0 |
| *Cerbera* | 22.6 |
| *Plumeria* | 23.4 |
| *Carissa* | 12.6 |
| *Catharanthus* | 11.0 |
| *Periploca* | 15.0 |
| *Hemidesmus* | 2.3 |
| *Chonemorpha* | 16.5 |
| *Apocynum* | 13.1 |
| *Wrightia* | 12.9 |
| *Mitrasacme* | 42.0 |
| *Mitreola* | 8.3 |
| *Cuscuta* | 87.3 |
| *Cressa* | 21.2 |
| *Evolvulus* | 15.3 |
| *Ipomoea* | 75.9 |
| *Convolvulus* | 36.6 |
| *Solanum* | 82.6 |
| *Przewalskia* | 7.1 |
| *Atropanthe* | 2.7 |
| *Capsicum* | 26.3 |
| *Datura* | 7.9 |
| *Nicotiana* | 43.4 |
| *Eucommia* | 43.9 |
| *Aucuba* | 27.1 |
| *Arnebia* | 31.7 |
| *Lithospermum* | 2.3 |
| *Onosma* | 4.6 |
| *Borago* | 10.6 |
| *Lennoa* | 5.4 |
| *Pholisma* | 11.4 |
| *Iodes* | 107.2 |
| *Primula* | 338.9 |
| *Androsace* | 52.4 |
| *Aegiceras* | 180.1 |
| *Ardisia* | 72.2 |
| *Myrsine* | 19.1 |
| *Embelia* | 8.6 |
| *Impatiens* | 107.1 |
| *Hydrocera* | 16.2 |
| *Agapetes* | 22.1 |
| *Gaultheria* | 21.4 |
| *Rhododendron* | 42.0 |
| *Camellia* | 440.4 |
| *Stewartia* | 31.1 |
| *Bruinsmia* | 39.7 |
| *Styrax* | 53.4 |
| *Alniphyllum* | 19.7 |
| *Actinidia* | 58.4 |
| *Pouteria* | 17.5 |
| *Synsepalum* | 7.1 |
| *Chrysophyllum* | 3.0 |
| *Sideroxylon* | 29.3 |
| *Diospyros* | 103.1 |
| *Bertholletia* | 49.1 |
| *Polemonium* | 20.4 |
| *Symplocos* | 16.6 |
| *Clethra* | 14.3 |
| *Cornus* | 150.5 |
| *Alangium* | 14.4 |
| *Hydrangea* | 132.8 |
| *Kirengeshoma* | 10.3 |
| *Whipplea* | 9.5 |
| *Loasa* | 40.2 |
| *Nasa* | 32.1 |
| *Mentzelia* | 15.8 |
| *Caiophora* | 10.1 |
| *Eucnide* | 11.1 |
| *Petalonyx* | 10.2 |
| *Hydrostachys* | 46.1 |
| *Camptotheca* | 15.3 |
| *Nyssa* | 28.3 |
| *Diplopanax* | 13.1 |
| *Grubbia* | 20.2 |
| *Curtisia* | 6.9 |
| *Atriplex* | 62.6 |
| *Chenopodium* | 171.2 |
| *Oxybasis* | 72.1 |
| *Dysphania* | 55.4 |
| *Suaeda* | 47.5 |
| *Salicornia* | 148.1 |
| *Patellifolia* | 8.4 |
| *Beta* | 29.7 |
| *Caroxylon* | 49.2 |
| *Silene* | 80.9 |
| *Agrostemma* | 7.6 |
| *Gypsophila* | 12.4 |
| *Dianthus* | 17.2 |
| *Colobanthus* | 23.5 |
| *Gymnocarpos* | 17.7 |
| *Pseudostellaria* | 11.3 |
| *Rheum* | 40.3 |
| *Rumex* | 177.1 |
| *Oxyria* | 8.0 |
| *Fagopyrum* | 45.9 |
| *Persicaria* | 25.5 |
| *Muehlenbeckia* | 23.1 |
| *Polygonum* | 10.4 |
| *Calligonum* | 18.9 |
| *Drosera* | 41.5 |
| *Aldrovanda* | 20.5 |
| *Dionaea* | 18.6 |
| *Rhipsalis* | 46.1 |
| *Lophocereus* | 10.8 |
| *Selenicereus* | 12.2 |
| *Portulaca* | 11.4 |
| *Cistanthe* | 8.8 |
| *Ptilotus* | 22.4 |
| *Celosia* | 22.0 |
| *Alternanthera* | 17.5 |
| *Amaranthus* | 1.0 |
| *Achyranthes* | 5.7 |
| *Limonium* | 27.7 |
| *Plumbago* | 29.0 |
| *Nepenthes* | 50.7 |
| *Bougainvillea* | 96.6 |
| *Nyctaginia* | 8.4 |
| *Boerhavia* | 4.2 |
| *Myricaria* | 26.2 |
| *Tamarix* | 13.9 |
| *Monococcus* | 30.9 |
| *Phytolacca* | 16.7 |
| *Petiveria* | 17.0 |
| *Tetragonia* | 6.1 |
| *Mesembryanthemum* | 10.0 |
| *Stegnosperma* | 15.3 |
| *Chunia* | 331.2 |
| *Mytilaria* | 15.2 |
| *Disanthus* | 14.0 |
| *Sinowilsonia* | 20.0 |
| *Rhodoleia* | 18.3 |
| *Corylopsis* | 9.1 |
| *Fortunearia* | 7.8 |
| *Loropetalum* | 4.3 |
| *Saxifraga* | 69.1 |
| *Chrysosplenium* | 44.6 |
| *Tanakaea* | 22.2 |
| *Micranthes* | 30.1 |
| *Bergenia* | 25.0 |
| *Tiarella* | 20.7 |
| *Mitella* | 16.5 |
| *Mukdenia* | 3.6 |
| *Sedum* | 54.2 |
| *Rhodiola* | 86.1 |
| *Crassula* | 21.0 |
| *Kalanchoe* | 7.2 |
| *Aeonium* | 14.1 |
| *Cotyledon* | 11.5 |
| *Orostachys* | 12.2 |
| *Phedimus* | 3.4 |
| *Sempervivum* | 7.8 |
| *Hylotelephium* | 3.9 |
| *Sinocrassula* | 5.4 |
| *Paeonia* | 76.0 |
| *Myriophyllum* | 33.8 |
| *Itea* | 28.4 |
| *Penthorum* | 121.1 |
| *Daphniphyllum* | 21.5 |
| *Cercidiphyllum* | 10.2 |
| *Altingia* | 26.9 |
| *Loranthus* | 40.0 |
| *Cecarria* | 18.7 |
| *Taxillus* | 40.1 |
| *Scurrula* | 21.1 |
| *Tolypanthus* | 16.8 |
| *Dendrophthoe* | 9.9 |
| *Helixanthera* | 13.1 |
| *Moquiniella* | 7.2 |
| *Nuytsia* | 30.4 |
| *Macrosolen* | 13.8 |
| *Elytranthe* | 13.1 |
| *Viscum* | 130.9 |
| *Osyris* | 27.2 |
| *Santalum* | 28.2 |
| *Schoepfia* | 48.3 |
| *Malania* | 46.8 |
| *Erythropalum* | 43.1 |
| *Dendrotrophe* | 18.8 |
| *Phacellaria* | 9.1 |
| *Pyrularia* | 27.1 |
| *Dillenia* | 61.2 |
| *Dendrobium* | 72.1 |
| *Bulbophyllum* | 19.9 |
| *Oberonioides* | 4.7 |
| *Oberonia* | 6.0 |
| *Liparis* | 5.1 |
| *Palmorchis* | 44.8 |
| *Epipactis* | 13.4 |
| *Neottia* | 7.5 |
| *Cymbidium* | 28.4 |
| *Geodorum* | 2.2 |
| *Dipodium* | 1.9 |
| *Corallorhiza* | 18.5 |
| *Phalaenopsis* | 4.2 |
| *Gastrochilus* | 2.8 |
| *Tainia* | 5.3 |
| *Gastrodia* | 0.1 |
| *Chamaegastrodia* | 25.2 |
| *Goodyera* | 8.8 |
| *Ludisia* | 5.8 |
| *Lankesterella* | 2.5 |
| *Cyclopogon* | 3.2 |
| *Prescottia* | 3.6 |
| *Platanthera* | 6.9 |
| *Habenaria* | 7.2 |
| *Corybas* | 6.8 |
| *Paphiopedilum* | 33.8 |
| *Phragmipedium* | 5.3 |
| *Vanilla* | 6.6 |
| *Apostasia* | 28.2 |
| *Agave* | 13.4 |
| *Anemarrhena* | 3.0 |
| *Polygonatum* | 8.1 |
| *Sansevieria* | 1.3 |
| *Asparagus* | 4.8 |
| *Cordyline* | 2.1 |
| *Milla* | 1.6 |
| *Allium* | 47.1 |
| *Hippeastrum* | 6.1 |
| *Lycoris* | 14.0 |
| *Narcissus* | 2.3 |
| *Agapanthus* | 2.3 |
| *Iris* | 23.6 |
| *Sisyrinchium* | 3.4 |
| *Geosiris* | 12.6 |
| *Aloe* | 2.9 |
| *Xanthorrhoea* | 2.9 |
| *Barnardia* | 1.9 |
| *Albuca* | 3.5 |
| *Molineria* | 0.7 |
| *Fargesia* | 155.9 |
| *Neomicrocalamus* | 1.0 |
| *Triticum* | 11.3 |
| *Secale* | 0.8 |
| *Alopecurus* | 1.6 |
| *Lolium* | 7.2 |
| *Avena* | 1.5 |
| *Lygeum* | 1.6 |
| *Oryza* | 17.8 |
| *Whiteochloa* | 1.6 |
| *Echinochloa* | 5.0 |
| *Panicum* | 5.9 |
| *Paspalum* | 1.7 |
| *Oncorachis* | 2.8 |
| *Tristachya* | 3.2 |
| *Tragus* | 7.9 |
| *Elytrophorus* | 2.7 |
| *Dregeochloa* | 1.1 |
| *Eriachne* | 7.2 |
| *Alloeochaete* | 1.4 |
| *Sartidia* | 1.2 |
| *Pharus* | 2.7 |
| *Anomochloa* | 3.8 |
| *Carex* | 17.4 |
| *Isolepis* | 11.9 |
| *Cyperus* | 2.3 |
| *Bolboschoenus* | 11.1 |
| *Eleocharis* | 4.0 |
| *Eriocaulon* | 11.7 |
| *Sparganium* | 21.2 |
| *Typha* | 3.6 |
| *Ananas* | 5.0 |
| *Flagellaria* | 3.9 |
| *Roscoea* | 2.0 |
| *Zingiber* | 3.0 |
| *Alpinia* | 1.2 |
| *Curcuma* | 2.3 |
| *Ensete* | 5.1 |
| *Pollia* | 8.0 |
| *Anigozanthos* | 8.4 |
| *Pontederia* | 4.5 |
| *Podococcus* | 1.2 |
| *Mauritia* | 6.8 |
| *Burmannia* | 56.3 |
| *Dioscorea* | 28.8 |
| *Trichopus* | 6.6 |
| *Aletris* | 2.7 |
| *Metanarthecium* | 4.4 |
| *Tacca* | 6.9 |
| *Lilium* | 28.5 |
| *Medeola* | 4.2 |
| *Calochortus* | 2.2 |
| *Tricyrtis* | 3.5 |
| *Fritillaria* | 7.6 |
| *Veratrum* | 10.6 |
| *Paris* | 11.8 |
| *Heloniopsis* | 4.5 |
| *Iphigenia* | 5.6 |
| *Alstroemeria* | 4.0 |
| *Luzuriaga* | 2.4 |
| *Campynema* | 6.4 |
| *Stemona* | 10.3 |
| *Croomia* | 4.7 |
| *Xerophyta* | 5.2 |
| *Acanthochlamys* | 5.3 |
| *Carludovica* | 2.1 |
| *Ottelia* | 56.3 |
| *Najas* | 13.4 |
| *Elodea* | 41.7 |
| *Halophila* | 22.9 |
| *Enhalus* | 34.0 |
| *Thalassia* | 13.4 |
| *Ruppia* | 51.9 |
| *Arisarum* | 6.6 |
| *Pinellia* | 10.5 |
| *Arisaema* | 16.4 |
| *Amorphophallus* | 4.2 |
| *Lemna* | 11.8 |
| *Wolffia* | 17.4 |
| *Orontium* | 7.4 |
| *Anthurium* | 6.2 |
| *Homalomena* | 8.3 |
| *Stylochaeton* | 3.8 |
| *Lasia* | 2.9 |
| *Zostera* | 17.8 |
| *Phyllospadix* | 10.2 |
| *Stuckenia* | 20.0 |
| *Sagittaria* | 8.0 |
| *Caldesia* | 3.8 |
| *Alisma* | 3.1 |
| *Aponogeton* | 10.6 |
| *Butomus* | 8.4 |
| *Acorus* | 6.7 |
| *Aconitum* | 14.6 |
| *Staphisagria* | 1.4 |
| *Anemone* | 10.8 |
| *Anemoclema* | 3.9 |
| *Ranunculus* | 7.8 |
| *Actaea* | 5.4 |
| *Nigella* | 4.2 |
| *Asteropyrum* | 8.1 |
| *Callianthemum* | 4.5 |
| *Helleborus* | 7.9 |
| *Caltha* | 3.8 |
| *Enemion* | 10.6 |
| *Urophysa* | 2.1 |
| *Thalictrum* | 14.9 |
| *Glaucidium* | 13.9 |
| *Coptis* | 9.1 |
| *Corydalis* | 55.1 |
| *Papaver* | 6.0 |
| *Meconopsis* | 6.8 |
| *Macleaya* | 6.0 |
| *Bongardia* | 8.1 |
| *Epimedium* | 20.0 |
| *Gymnospermium* | 5.9 |
| *Berberis* | 1.8 |
| *Achlys* | 8.1 |
| *Diphylleia* | 7.3 |
| *Jeffersonia* | 2.0 |
| *Stephania* | 18.2 |
| *Sinomenium* | 10.3 |
| *Tinospora* | 4.9 |
| *Decaisnea* | 9.9 |
| *Circaeaster* | 19.0 |
| *Kingdonia* | 5.2 |
| *Euptelea* | 5.1 |
| *Cassytha* | 9.9 |
| *Beilschmiedia* | 25.8 |
| *Phoebe* | 32.0 |
| *Cinnamomum* | 1.3 |
| *Lindera* | 9.0 |
| *Aristolochia* | 22.3 |
| *Saruma* | 1.2 |
| *Gymnotheca* | 3.0 |
| *Piper* | 7.7 |
| *Annona* | 13.5 |
| *Uvaria* | 2.8 |
| *Chieniodendron* | 2.8 |
| *Greenwayodendron* | 2.5 |
| *Magnolia* | 20.2 |
| *Liriodendron* | 0.7 |
| *Meliosma* | 7.8 |
| *Sabia* | 7.4 |
| *Nelumbo* | 6.2 |
| *Grevillea* | 5.0 |
| *Platanus* | 4.0 |
| *Buxus* | 33.4 |
| *Pachysandra* | 1.9 |
| *Ceratophyllum* | 27.1 |
| *Trochodendron* | 4.1 |
| *Tetracentron* | 1.8 |
| *Chloranthus* | 5.7 |
| *Schisandra* | 1.1 |
| *Amborella* | 1.4 |
| *Dendrolobium* | 6.1 |
| *Echinosophora* | 2.8 |
| *Libidibia* | 3.1 |
| *Gymnocladus* | 8.6 |
| *Erythrophleum* | 4.5 |
| *Bennettiodendron* | 5.6 |
| *Azara* | 4.2 |
| *Gaulettia* | 2.0 |
| *Lagenaria* | 2.6 |
| *Baijiania* | 5.3 |
| *Myrcia* | 707.8 |
| *Salvertia* | 291.6 |
| *Leiospora* | 8.4 |
| *Sinapis* | 4.0 |
| *Barbarea* | 4.1 |
| *Ionopsidium* | 2.8 |
| *Noccaea* | 3.7 |
| *Oreophyton* | 3.8 |
| *Kernera* | 2.9 |
| *Gynandropsis* | 4.1 |
| *Vasconcellea* | 14.8 |
| *Nephelium* | 6.1 |
| *Theobroma* | 18.8 |
| *Artemisia* | 416.0 |
| *Leontopodium* | 94.3 |
| *Helichrysum* | 57.8 |
| *Anaphalis* | 78.8 |
| *Gynura* | 10.6 |
| *Crassocephalum* | 132.8 |
| *Siphocampylus* | 13.5 |
| *Leycesteria* | 3.5 |
| *Tongoloa* | 6.8 |
| *Eleutherococcus* | 14.6 |
| *Mentha* | 5.8 |
| *Barleria* | 11.1 |
| *Notelaea* | 5.8 |
| *Dorcoceras* | 6.6 |
| *Puchiumazus* | 3.0 |
| *Dodartia* | 2.6 |
| *Metagentiana* | 3.1 |
| *Fosbergia* | 5.6 |
| *Uncaria* | 4.8 |
| *Atropa* | 5.5 |
| *Physochlaina* | 3.2 |
| *Lycium* | 0.7 |
| *Elingamita* | 7.2 |
| *Lysimachia* | 16.8 |
| *Monotropa* | 1.3 |
| *Saurauia* | 7.9 |
| *Mimusops* | 11.5 |
| *Deutzia* | 8.3 |
| *Philadelphus* | 2.7 |
| *Rodgersia* | 3.3 |
| *Talinum* | 5.0 |
| *Eulophia* | 0.8 |
| *Masdevallia* | 2.2 |
| *Cephalantheropsis* | 2.9 |
| *Sobralia* | 0.1 |
| *Yucca* | 5.8 |
| *Chlorogalum* | 3.6 |
| *Chlorophytum* | 3.5 |
| *Oziroe* | 0.6 |
| *Chasechloa* | 1.1 |
| *Hypolytrum* | 14.4 |
| *Kaempferia* | 2.2 |
| *Musa* | 8.3 |
| *Trillium* | 4.7 |
| *Xerophyllum* | 2.0 |
| *Lloydia* | 1.1 |
| *Colchicum* | 1.6 |
| *Gloriosa* | 0.5 |
| *Pistia* | 3.4 |
| *Clematis* | 2.9 |
| *Adonis* | 2.5 |
| *Dichocarpum* | 1.5 |
| *Leptopyrum* | 0.3 |
| *Coreanomecon* | 2.3 |
| *Caryodaphnopsis* | 1.8 |
| *Macadamia* | 4.0 |
| *Sarcandra* | 2.5 |
| *Pemphis* | 108.3 |
| *Stebbinsia* | 121.8 |
| *Xanthium* | 77.6 |
| *Helianthus* | 378.9 |
| *Solidago* | 29.8 |
| *Nannoglottis* | 89.2 |
| *Erigeron* | 69.6 |
| *Parastrephia* | 49.6 |
| *Lagenophora* | 40.3 |
| *Pericallis* | 42.0 |
| *Jacobaea* | 264.8 |
| *Emilia* | 36.4 |
| *Senecio* | 39.5 |
| *Sinosenecio* | 289.9 |
| *Chrysanthemum* | 1104.0 |
| *Phaeostigma* | 22.6 |
| *Synurus* | 53.2 |
| *Arctium* | 103.9 |
| ***Arctotheca*** | **25.1** |
| *Myripnois* | 83.3 |
| *Daucus* | 11.9 |
| *Oresitrophe* | 1.1 |
| *Halimodendron* | 3.8 |
| *Eriosema* | 3.4 |
| *Kummerowia* | 3.2 |
| *Thermopsis* | 1.1 |
| *Piptadenia* | 13.4 |
| *Parkia* | 1.4 |
| *Pithecellobium* | 4.5 |
| *Tamarindus* | 3.5 |
| *Mitostemma* | 1.0 |
| *Xylosma* | 3.1 |
| *Olmediella* | 2.3 |
| *Parastemon* | 3.6 |
| *Exellodendron* | 0.4 |
| *Aspidopterys* | 8.9 |
| *Cyclocarya* | 0.9 |
| *Ostrya* | 11.0 |
| *Corallocarpus* | 5.5 |
| *Angophora* | 37820.5 |
| *Lachnoloma* | 3.3 |
| *Spryginia* | 14.0 |
| *Leptaleum* | 2.3 |
| *Fourraea* | 2.3 |
| *Horwoodia* | 3.6 |
| *Litwinowia* | 5.9 |
| *Iskandera* | 1.7 |
| *Clypeola* | 5.8 |
| *Clausia* | 56.6 |
| *Dithyrea* | 3.1 |
| *Thulinella* | 2.5 |
| *Akania* | 7.8 |
| *Aglaia* | 6.3 |
| *Cedrela* | 3.8 |
| *Boswellia* | 2.2 |
| *Urena* | 5.1 |
| *Alcea* | 0.8 |
| *Tilia* | 3.8 |
| *Pachira* | 258.5 |
| *Parashorea* | 2.6 |
| *Helianthemum* | 3.6 |
| *Dendroseris* | 167.7 |
| *Oritrophium* | 48.4 |
| *Cynara* | 122.9 |
| *Carthamus* | 21.2 |
| *Centaurea* | 36.9 |
| *Pertya* | 50.7 |
| *Ainsliaea* | 33.3 |
| *Centropogon* | 5.4 |
| *Hanabusaya* | 1.2 |
| *Tetradoxa* | 1.4 |
| *Dipelta* | 4.2 |
| *Brandisia* | 2.7 |
| *Paraphlomis* | 7.4 |
| *Chelonopsis* | 2.8 |
| *Jacaranda* | 2.8 |
| *Schrebera* | 2.9 |
| *Picconia* | 30.9 |
| *Myxopyrum* | 4.0 |
| *Neopicrorhiza* | 0.8 |
| *Lancea* | 2.5 |
| *Lomatogoniopsis* | 6.1 |
| *Rauvolfia* | 10.4 |
| *Alstonia* | 9.2 |
| *Calibrachoa* | 1.9 |
| *Jamesia* | 2.1 |
| *Davidia* | 5.8 |
| *Haloxylon* | 6.4 |
| *Acleisanthes* | 3.8 |
| *Graptopetalum* | 2.0 |
| *Erycina* | 3.1 |
| *Changnienia* | 2.5 |
| *Calanthe* | 4.6 |
| *Vanda* | 1.3 |
| *Eurystyles* | 3.4 |
| *Ponerorchis* | 0.7 |
| *Lecanorchis* | 0.3 |
| *Setaria* | 0.7 |
| *Urochloa* | 0.4 |
| *Zea* | 5.5 |
| *Dactyloctenium* | 3.1 |
| *Merxmuellera* | 0.3 |
| *Streptogyna* | 0.9 |
| *Ehrharta* | 1.2 |
| *Joinvillea* | 0.6 |
| *Ypsilandra* | 1.0 |
| *Amana* | 0.9 |
| *Spirodela* | 3.8 |
| *Zamioculcas* | 2.5 |
| *Colocasia* | 0.2 |
| *Oxygraphis* | 1.9 |
| *Hepatica* | 3.5 |
| *Delphinium* | 0.6 |
| *Semiaquilegia* | 2.4 |
| *Ranzania* | 2.8 |
| *Pericampylus* | 2.5 |
| *Stauntonia* | 1.4 |
| *Sinofranchetia* | 2.3 |
| *Litsea* | 10.7 |
| *Calycanthus* | 3.0 |
| *Idiospermum* | 1.5 |
| *Houttuynia* | 2.4 |
| *Pseudowintera* | 1.0 |
| *Helicia* | 4.5 |
| *Uraria* | 2.5 |
| *Kissenia* | 5.9 |
| *Salsola* | 8.6 |
| *Afrolicania* | 4.4 |
| *Acioa* | 1.2 |
| *Osbeckia* | 14.0 |
| *Barthea* | 11.5 |
| *Miconia* | 16.9 |
| *Schrenkiella* | 2.5 |
| *Malcolmia* | 1.1 |
| *Boechera* | 7.3 |
| *Bunias* | 2.2 |
| *Erysimum* | 2.5 |
| *Cleome* | 7.8 |
| *Thespesia* | 5.1 |
| *Balanocarpus* | 6.4 |
| *Lapsanastrum* | 318.3 |
| *Tithonia* | 28.6 |
| *Sigesbeckia* | 7.2 |
| *Laestadia* | 29.0 |
| *Llerasia* | 30.7 |
| *Neopallasia* | 119.1 |
| *Isodon* | 0.5 |
| *Stachys* | 5.7 |
| *Cymaria* | 2.6 |
| *Pleonotoma* | 3.0 |
| *Foonchewia* | 7.1 |
| *Neolamarckia* | 4.4 |
| *Gymnema* | 2.2 |
| *Tubocapsicum* | 0.9 |
| *Hosta* | 9.3 |
| *Aphyllanthes* | 3.1 |
| *Sciaphila* | 0.3 |
| *Gymnaconitum* | 1.3 |
| *Eranthis* | 3.4 |
| *Hylomecon* | 0.9 |
| *Plagiorhegma* | 1.6 |
| *Saururus* | 2.0 |
| *Tasmannia* | 2.7 |
| *Trithuria* | 0.6 |
| *Diphelypaea* | 1.3 |
| *Carpenteria* | 2.6 |
| *Nassella* | 1.7 |
| *Achnatherum* | 1.5 |
| *Brachypodium* | 0.8 |
| *Eria* | 0.3 |
| *Wolffiella* | 1.3 |
| *Spathiphyllum* | 0.6 |
| *Torminalis* | 0.0 |
| *Decorsea* | 1.0 |
| *Cakile* | 0.7 |
| *Swietenia* | 7.4 |
| *Trachyspermum* | 0.2 |
| *Pterygocalyx* | 2.2 |
| *Dactylorhiza* | 0.1 |
| *Curculigo* | 0.6 |
| *Calepina* | 2.0 |
| *Withania* | 16.7 |
| *Triodia* | 18.0 |
| *Eragrostis* | 4.0 |
| *Coelachne* | 0.8 |
| *Leptaspis* | 1.2 |
| *Maianthemum* | 1.1 |
| *Silybum* | 84.4 |
| *Seguieria* | 8.5 |
| *Dunbaria* | 2.0 |
| *Urariopsis* | 1.7 |
| *Carrierea* | 2.7 |
| *Melastoma* | 21.9 |
| *Blastus* | 7.4 |
| *Anzhengxia* | 4.3 |
| *Cymatocarpus* | 7.6 |
| *Rhammatophyllum* | 1.9 |
| *Raphanus* | 2.3 |
| *Dimorphocarpa* | 8.1 |
| *Exhalimolobos* | 4.0 |
| *Brayopsis* | 2.6 |
| *Anelsonia* | 4.7 |
| *Conringia* | 1.6 |
| *Thlaspi* | 2.2 |
| *Sisymbrium* | 1.4 |
| *Pachycladon* | 2.6 |
| *Cotinus* | 1.7 |
| *Abelmoschus* | 5.7 |
| *Ceiba* | 1.7 |
| *Stellera* | 2.1 |
| *Eclipta* | 4.3 |
| *Heteroplexis* | 19.7 |
| *Conyza* | 6.9 |
| *Hinterhubera* | 26.3 |
| *Floscaldasia* | 11.8 |
| *Laennecia* | 20.5 |
| *Carduus* | 8.5 |
| *Linnaea* | 2.6 |
| *Apium* | 3.3 |
| *Plectranthus* | 1.4 |
| *Perilla* | 2.9 |
| *Forestiera* | 3.0 |
| *Priogymnanthus* | 1.9 |
| *Silvianthus* | 2.4 |
| *Halenia* | 5.6 |
| *Pergularia* | 5.7 |
| *Vincetoxicum* | 2.5 |
| *Petunia* | 5.3 |
| *Eurya* | 2.0 |
| *Carnegiea* | 3.5 |
| *Semiliquidambar* | 26.1 |
| *Astilboides* | 2.9 |
| *Cephalanthera* | 0.5 |
| *Cypripedium* | 2.4 |
| *Leptagrostis* | 0.8 |
| *Amphipogon* | 0.4 |
| *Gelidocalamus* | 3.7 |
| *Catapodium* | 9.8 |
| *Cautleya* | 2.8 |
| *Scoliopus* | 0.8 |
| *Tulipa* | 1.1 |
| *Disporum* | 1.8 |
| *Isopyrum* | 1.4 |
| *Nandina* | 0.7 |
| *Arcangelisia* | 2.2 |
| *Alseodaphnopsis* | 2.7 |
| *Cryptocarya* | 0.8 |
| *Drimys* | 1.6 |
| *Callianthe* | 1.3 |
| *Youngia* | 35.0 |
| *Iostephane* | 13.8 |
| *Blakiella* | 8.9 |
| *Scopolia* | 0.6 |
| *Tapeinosperma* | 12.8 |
| *Clivia* | 0.4 |
| *Zeugites* | 1.3 |
| *Sporobolus* | 27.6 |
| *Styppeiochloa* | 2.2 |
| *Aristida* | 2.8 |
| *Smilax* | 4.3 |
| *Sinopodophyllum* | 0.8 |
| *Menispermum* | 2.7 |
| *Flacourtia* | 1.9 |
| *Octoceras* | 2.0 |
| *Colquhounia* | 9.9 |
| *Physalis* | 1.8 |
| *Oxychloris* | 2.7 |
| *Chloris* | 1.7 |
| *Eleusine* | 0.7 |
| *Astrebla* | 3.6 |
| *Enteropogon* | 0.5 |
| *Melanocenchris* | 3.0 |
| *Tripogonella* | 1.2 |
| *Oropetium* | 0.5 |
| *Trichoneura* | 0.5 |
| *Aeluropus* | 0.2 |
| *Vaseyochloa* | 0.5 |
| *Enneapogon* | 1.1 |
| *Lecomtella* | 0.6 |
| *Oplismenus* | 3.6 |
| *Paraneurachne* | 1.1 |
| *Chasmanthium* | 0.7 |
| *Limnopoa* | 1.4 |
| *Pratochloa* | 0.3 |
| *Lamarckia* | 0.6 |
| *Nardus* | 0.3 |
| *Leersia* | 0.3 |
| *Syndiclis* | 4.2 |
| *Tetrataenium* | 0.5 |
| *Astelia* | 1.1 |
| *Stenospermation* | 1.0 |
| *Heterotis* | 4.0 |
| *Streptoloma* | 2.1 |
| *Alliaria* | 1.2 |
| *Hilliella* | 1.8 |
| *Litchi* | 1.2 |
| *Abutilon* | 1.0 |
| *Excentrodendron* | 2.9 |
| *Atuna* | 0.9 |
| *Diabelia* | 1.3 |
| *Melanosciadium* | 0.9 |
| *Ostericum* | 1.7 |
| *Hansenia* | 5.8 |
| *Metapanax* | 2.9 |
| *Stenogyne* | 1.6 |
| *Leonurus* | 2.0 |
| *Halesia* | 4.2 |
| *Vitellaria* | 2.3 |
| *Euryodendron* | 2.6 |
| *Mirabilis* | 1.5 |
| *Cattleya* | 1.4 |
| *Oncidium* | 1.0 |
| *Beschorneria* | 3.8 |
| *Hesperoyucca* | 2.8 |
| *Eremurus* | 1.7 |
| *Hyacinthoides* | 1.0 |
| *Phyllostachys* | 2.4 |
| *Bambusa* | 1.6 |
| *Hitchcockella* | 0.2 |
| *Froesiochloa* | 0.8 |
| *Thinopyrum* | 0.9 |
| *Axonopus* | 1.2 |
| *Danthoniopsis* | 0.6 |
| *Eustachys* | 0.4 |
| *Uniola* | 0.8 |
| *Elaeis* | 1.4 |
| *Areca* | 1.3 |
| *Clintonia* | 0.9 |
| *Dieffenbachia* | 0.6 |
| *Paraquilegia* | 2.7 |
| *Aquilegia* | 6.6 |
| *Caulophyllum* | 0.9 |
| *Dysosma* | 2.1 |
| *Leontice* | 1.6 |
| *Nothaphoebe* | 11.1 |
| *Chimonanthus* | 1.2 |
| *Shangrilaia* | 0.6 |
| *Aubrieta* | 0.1 |
| *Camelina* | 0.9 |
| *Arabidella* | 0.2 |
| *Eudema* | 0.7 |
| *Merrillia* | 0.1 |
| *Malva* | 1.2 |
| *Cytinus* | 0.5 |
| *Dolichos* | 0.8 |
| *Westoniella* | 4.8 |
| *Ajania* | 6.5 |
| *Sinadoxa* | 1.0 |
| *Craniotome* | 0.6 |
| *Trompettia* | 0.6 |
| *Pterostyrax* | 0.8 |
| *Changiostyrax* | 1.7 |
| *Lucuma* | 3.5 |
| *Parrotia* | 0.6 |
| *Liquidambar* | 3.0 |
| *Anathallis* | 0.9 |
| *Thunia* | 0.2 |
| *Bletilla* | 0.2 |
| *Aspidogyne* | 0.2 |
| *Sauroglossum* | 0.3 |
| *Dracaena* | 0.6 |
| *Chusquea* | 0.1 |
| *Bromus* | 1.8 |
| *Chikusichloa* | 0.4 |
| *Alloteropsis* | 0.5 |
| *Streptochaeta* | 1.0 |
| *Trachycarpus* | 1.6 |
| *Actinodaphne* | 1.9 |
| *Barclaya* | 0.7 |
| *Nymphaea* | 0.7 |
| *Christia* | 0.7 |
| *Cyamopsis* | 6.7 |
| *Leucomeris* | 11.6 |
| *Petroselinum* | 1.2 |
| *Thrixspermum* | 0.1 |
| *Speirantha* | 0.3 |
| *Indocalamus* | 0.1 |
| *Hordeum* | 0.5 |
| *Melica* | 0.2 |
| *Zizania* | 0.2 |
| *Cenchrus* | 0.5 |
| *Sacciolepis* | 0.2 |
| *Typhonium* | 0.2 |
| *Carlephyton* | 0.1 |
| *Morettia* | 0.3 |
| *Toona* | 0.6 |
| *Arracacia* | 0.8 |
| *Agastache* | 1.0 |
| *Nestegis* | 4.4 |
| *Iochroma* | 5.1 |
| *Anthericum* | 0.2 |
| *Nolina* | 1.0 |
| *Dendrocalamus* | 12.5 |
| *Imperata* | 0.6 |
| *Monstera* | 0.5 |
| *Goldbachia* | 0.2 |
| *Ocimum* | 1.1 |
| *Perkinsiodendron* | 1.7 |
| *Hesperocallis* | 0.3 |
| *Streptopus* | 0.2 |
| *Aloidendron* | 0.1 |
| *Poa* | 0.1 |
| *Humbertochloa* | 0.1 |
| *Arthraxon* | 0.1 |
| *Stenotaphrum* | 0.2 |
| *Triraphis* | 0.1 |
| *Xanthosoma* | 0.2 |
| *Alseodaphne* | 0.2 |
| *Glandularia* | 0.1 |
| *Cynosurus* | 1.5 |
| *Puccinellia* | 0.2 |
| *Isatis* | 0.1 |
| *Cheirodendron* | 1.0 |
| *Sphagneticola* | 0.9 |
| *Aztecaster* | 1.9 |
| *Opisthopappus* | 4.3 |
| *Fatsia* | 0.4 |
| *Phyllostegia* | 0.9 |
| *Comoranthus* | 1.2 |
| *Abeliophyllum* | 0.3 |
| *Forsythia* | 1.2 |
| *Cremastra* | 0.1 |
| *Galearis* | 0.2 |
| *Convallaria* | 0.2 |
| *Beesia* | 0.2 |
| *Chengiodendron* | 0.6 |
| *Urochondra* | 12.2 |
| *Orinus* | 0.8 |
| *Eremochloa* | 0.4 |
| *Loudetiopsis* | 1.3 |
| *Dichaetaria* | 0.8 |
| *Ampelocalamus* | 0.5 |
| *Gynerium* | 1.1 |
| *Garnotia* | 0.4 |
| *Stipagrostis* | 0.3 |
| *Rehia* | 0.2 |
| *Leymus* | 0.7 |
| *Hygroryza* | 0.1 |
| *Alyssopsis* | 0.3 |
| *Gymnadenia* | 0.2 |
| *Hesperaloe* | 0.2 |
| *Ophiopogon* | 0.2 |
| *Agrostis* | 0.1 |
| *Neolitsea* | 0.2 |
| *Phyllorachis* | 0.1 |
| *Cymbopogon* | 0.2 |
| *Verbena* | 0.3 |
| *Oreorchis* | 0.2 |
|  |  |
| **RefSeq db Species level** | |
| Taxon | Mean est. num. reads |
| ***Prunus dulcis*** | **128869.5** |
| *Prunus fruticosa* | 2087.2 |
| *Prunus yedoensis* | 9164.1 |
| *Prunus zippeliana* | 1193.2 |
| *Prunus domestica* | 1950.8 |
| *Prunus mira* | 2073.8 |
| *Prunus fasciculata* | 1796.1 |
| *Prunus padus* | 3162.5 |
| *Prunus humilis* | 4632.8 |
| *Prunus cerasoides* | 1564.9 |
| *Prunus salicina* | 831.4 |
| *Prunus matuurae* | 4878.5 |
| *Prunus gongshanensis* | 445.8 |
| *Prunus rufa* | 4071.5 |
| *Prunus armeniaca* | 188.3 |
| *Prunus himalaica* | 212.5 |
| *Prunus pseudocerasus* | 1643.2 |
| *Prunus tenella* | 218.9 |
| *Prunus serotina* | 114.9 |
| *Prunus avium* | 129.6 |
| *Prunus mongolica* | 246.4 |
| *Prunus pedunculata* | 284.7 |
| *Prunus discadenia* | 293.8 |
| *Prunus wilsonii* | 210.8 |
| *Prunus davidiana* | 148.9 |
| *Prunus kansuensis* | 151.0 |
| *Prunus triloba* | 121.1 |
| *Prunus persica* | 121.9 |
| *Prunus fujianensis* | 125.5 |
| *Prunus japonica* | 875.8 |
| *Prunus takesimensis* | 252.9 |
| *[Cerasus] fengyangshanica* | 65.8 |
| *Prunus dielsiana* | 201.8 |
| *Prunus jingningensis* | 201.2 |
| *Prunus maximowiczii* | 114.2 |
| *Prunus hypoxantha* | 25.9 |
| *Crataegus pinnatifida* | 6035.1 |
| *Crataegus kansuensis* | 43.6 |
| *Crataegus cuneata* | 14.4 |
| *Chaenomeles sinensis* | 182.4 |
| *Vauquelinia pauciflora* | 195.6 |
| *Vauquelinia australis* | 38.6 |
| *Malus ioensis* | 27.4 |
| *Malus hupehensis* | 485.4 |
| *Malus doumeri* | 85.7 |
| *Malus florentina* | 10.1 |
| *Malus toringoides* | 85.6 |
| *Rhaphiolepis bibas* | 53.6 |
| *Rhaphiolepis malipoensis* | 51.8 |
| *Rhaphiolepis seguinii* | 17.4 |
| *Rhaphiolepis laoshanica* | 13.3 |
| *Rhaphiolepis henryi* | 44.0 |
| *Rhaphiolepis salwinensis* | 17.5 |
| *Rhaphiolepis major* | 64.7 |
| *Kageneckia angustifolia* | 49.1 |
| *Kageneckia lanceolata* | 19.0 |
| *Kageneckia oblonga* | 5.9 |
| *Cotoneaster silvestrii* | 58.9 |
| *Cotoneaster buxifolius* | 89.7 |
| *Cotoneaster schantungensis* | 0.5 |
| *Cotoneaster wilsonii* | 101.4 |
| *Photinia sorbifolia* | 163.6 |
| *Photinia integrifolia* | 6.4 |
| *Photinia lochengensis* | 26.9 |
| *Sorbus aucuparia* | 39.5 |
| *Sorbus insignis* | 29.4 |
| *Sorbus chamaemespilus* | 17.2 |
| *Amelanchier asiatica* | 13.4 |
| *Amelanchier interior* | 362.9 |
| *Pyracantha fortuneana* | 49.4 |
| *Pyrus pyrifolia* | 84.4 |
| *Dichotomanthes tristaniicarpa* | 38.1 |
| *Pourthiaea amphidoxa* | 23.2 |
| *Pourthiaea tomentosa* | 9.5 |
| *Aronia arbutifolia* | 39.3 |
| *Phippsiomeles mexicana* | 4.8 |
| *Cydonia oblonga* | 30.5 |
| *Docynia delavayi* | 373.8 |
| *Malacomeles denticulata* | 25.0 |
| *Spiraea mongolica* | 340.7 |
| *Spiraea insularis* | 100.3 |
| *Sibiraea angustata* | 144.9 |
| *Pentactina rupicola* | 71.0 |
| *Sorbaria arborea* | 577.8 |
| *Neillia incisa* | 314.4 |
| *Gillenia stipulata* | 73.7 |
| *Gillenia trifoliata* | 32.1 |
| *Rubus hybrid cultivar* | 62.6 |
| *Rubus peltatus* | 30.3 |
| *Rubus amabilis* | 25.1 |
| *Rubus eucalyptus* | 26.6 |
| *Rubus lineatus* | 22.2 |
| *Rubus irritans* | 34.2 |
| *Rubus sachalinensis* | 16.9 |
| *Rubus xanthocarpus* | 12.7 |
| *Rubus taiwanicola* | 113.9 |
| *Rubus xanthoneurus* | 31.5 |
| *Rubus tsangii* | 7.8 |
| *Rubus boninensis* | 10.1 |
| *Rubus pileatus* | 9.1 |
| *Rubus wallichianus* | 18.2 |
| *Rubus niveus* | 7.1 |
| *Rosa laevigata* | 634.0 |
| *Rosa banksiae* | 7.1 |
| *Rosa roxburghii* | 8.6 |
| *Rosa praelucens* | 6.8 |
| *Rosa maximowicziana* | 64.6 |
| *Rosa davurica* | 25.7 |
| *Potentilla lineata* | 31.4 |
| *[Argentina] phanerophlebia* | 28.8 |
| *Potentilla ancistrifolia* | 37.2 |
| *Potentilla centigrana* | 33.8 |
| *Potentilla suavis* | 23.4 |
| *Potentilla hebiichigo* | 19.7 |
| *Potentilla sischanensis* | 18.5 |
| *Potentilla indica* | 21.9 |
| *Sibbaldia aphanopetala* | 45.5 |
| *Alchemilla pedata* | 23.8 |
| *Alchemilla argyrophylla* | 4.2 |
| *Sibbaldianthe bifurca* | 18.1 |
| *Sibbaldianthe adpressa* | 11.6 |
| *Fragaria orientalis* | 26.4 |
| *Fragaria vesca* | 19.2 |
| *Fragaria x ananassa* | 71.6 |
| *Drymocallis saviczii* | 11.8 |
| *Comarum salesovianum* | 13.4 |
| *Potaninia mongolica* | 8.9 |
| *Sanguisorba filiformis* | 40.9 |
| *Sanguisorba sitchensis* | 39.4 |
| *Sanguisorba hakusanensis* | 17.2 |
| *Bencomia exstipulata* | 34.4 |
| *Agrimonia nipponica* | 24.8 |
| *Agrimonia coreana* | 23.7 |
| *Geum rupestre* | 107.4 |
| *Geum macrophyllum* | 53.1 |
| *Pilea microphylla* | 66.6 |
| *Pilea pumila* | 66.0 |
| *Pilea glauca (nom. nud.)* | 149.9 |
| *Pilea thymifolia* | 53.2 |
| *Pilea cavaleriei* | 25.7 |
| *Pilea cadierei* | 43.2 |
| *Pilea plataniflora* | 67.0 |
| *Pilea peperomioides* | 57.8 |
| *Pilea mollis* | 42.8 |
| *Pilea peploides* | 50.7 |
| *Elatostema dissectum* | 117.6 |
| *Boehmeria spicata* | 27.7 |
| *Boehmeria umbrosa* | 116.4 |
| *Boehmeria nivea* | 12.5 |
| *Urtica lobatifolia* | 45.1 |
| *Poikilospermum lanceolatum* | 41.8 |
| *Pouzolzia hirta* | 38.9 |
| *Procris crenata* | 34.7 |
| *Cecropia pachystachya* | 13.5 |
| *Debregeasia orientalis* | 3.4 |
| *Pteroceltis tatarinowii* | 119.3 |
| *Celtis sinensis* | 23.2 |
| *Celtis biondii* | 8.6 |
| *Aphananthe aspera* | 28.7 |
| *Gironniera subaequalis* | 42.0 |
| *Lozanella enantiophylla* | 33.4 |
| *Cannabis sativa* | 22.6 |
| *Humulus scandens* | 19.2 |
| *Humulus lupulus* | 5.1 |
| *Parasponia rugosa* | 22.0 |
| *Trema orientale* | 16.7 |
| *Broussonetia kaempferi* | 14.4 |
| *Broussonetia kurzii* | 14.3 |
| *Broussonetia kazinoki x Broussonetia papyrifera* | 14.3 |
| *Broussonetia luzonica* | 11.0 |
| *Ficus religiosa* | 16.3 |
| *Ficus racemosa* | 5.8 |
| *Ficus microcarpa* | 27.0 |
| *Ficus lyrata* | 6.8 |
| *Ficus heteromorpha* | 1.1 |
| *Artocarpus integer* | 23.2 |
| *Artocarpus hypargyreus* | 10.7 |
| *Artocarpus petelotii* | 5.6 |
| *Trophis caucana* | 18.0 |
| *Trophis scandens* | 11.0 |
| *Maclura tricuspidata* | 32.4 |
| *Streblus indicus* | 49.9 |
| *Morus celtidifolia* | 6.9 |
| *Antiaris toxicaria* | 21.4 |
| *Rhamnus globosa* | 20.3 |
| *Berchemia lineata* | 15.9 |
| *Ventilago leiocarpa* | 48.8 |
| *Ziziphus attopensis* | 55.8 |
| *Ziziphus jujuba* | 36.9 |
| *Ziziphus incurva* | 18.1 |
| *Ulmus lanceifolia* | 12.9 |
| *Ulmus macrocarpa* | 134.2 |
| *Ulmus gaussenii* | 27.3 |
| *Ulmus parvifolia* | 4.2 |
| *Chaetachme aristata* | 19.5 |
| *Hippophae gyantsensis* | 14.1 |
| *Hippophae rhamnoides* | 3.9 |
| *Hippophae neurocarpa* | 24.4 |
| *Elaeagnus angustifolia* | 19.6 |
| *Elaeagnus pungens* | 6.7 |
| *Barbeya oleoides* | 55.6 |
| *Lathyrus sativus* | 17.3 |
| *Lathyrus pubescens* | 13.1 |
| *Lathyrus tingitanus* | 10.6 |
| *Lathyrus inconspicuus* | 9.3 |
| *Lathyrus odoratus* | 10.9 |
| *Lathyrus graminifolius* | 9.5 |
| *Lathyrus clymenum* | 6.6 |
| *Lathyrus venosus* | 7.6 |
| *Lathyrus palustris* | 4.7 |
| *Lathyrus ochroleucus* | 6.3 |
| *Vicia costata* | 27.6 |
| *Vicia cracca* | 15.9 |
| *Vicia sepium* | 7.3 |
| *Vicia sativa* | 14.3 |
| *Pisum sativum* | 27.8 |
| *Lens culinaris* | 11.4 |
| *Vavilovia formosa* | 9.7 |
| *Trifolium boissieri* | 24.3 |
| *Trifolium subterraneum* | 15.0 |
| *Medicago truncatula* | 18.3 |
| *Medicago edgeworthii* | 11.0 |
| *Parochetus communis* | 13.9 |
| *Melilotus albus* | 8.3 |
| *Galega officinalis* | 13.4 |
| *Sphaerophysa salsula* | 62.9 |
| *Lessertia frutescens* | 5.9 |
| *Caragana kozlowii* | 15.6 |
| *Hedysarum taipeicum* | 20.7 |
| *Onobrychis viciifolia* | 16.4 |
| *Alhagi sparsifolia* | 13.9 |
| *Cicer arietinum* | 12.3 |
| *Lotus japonicus* | 19.8 |
| *Securigera varia* | 31.4 |
| *Sesbania cannabina* | 23.9 |
| *Robinia pseudoacacia* | 20.4 |
| *Centrosema pubescens* | 19.1 |
| *Spatholobus suberectus* | 9.6 |
| *Spatholobus pulcher* | 9.4 |
| *Erythrina crista-galli* | 23.4 |
| *Vigna subterranea* | 9.3 |
| *Vigna angularis* | 1.5 |
| *Canavalia gladiata* | 31.7 |
| *Mucuna macrocarpa* | 16.9 |
| *Phaseolus vulgaris* | 26.0 |
| *Hardenbergia violacea* | 12.1 |
| *Pachyrhizus erosus* | 17.1 |
| *Apios americana* | 12.6 |
| *Lablab purpureus* | 7.6 |
| *Haymondia wallichii* | 10.0 |
| *Cajanus crassus* | 5.7 |
| *Psophocarpus tetragonolobus* | 12.9 |
| *Amphicarpaea edgeworthii* | 12.9 |
| *Kennedia prostrata* | 6.8 |
| *Austrosteenisia blackii* | 14.8 |
| *Philenoptera violacea* | 13.7 |
| *Dahlstedtia araripensis* | 18.5 |
| *Pongamia pinnata* | 14.3 |
| *Aganope dinghuensis* | 20.3 |
| *Millettia dura* | 12.0 |
| *Derris harrowiana* | 13.4 |
| *Alysicarpus vaginalis* | 13.2 |
| *Tadehagi triquetrum* | 6.7 |
| *Grona styracifolia* | 3.4 |
| *Ohwia caudata* | 4.6 |
| *Phyllodium pulchellum* | 4.1 |
| *Hanslia ormocarpoides* | 3.2 |
| *Campylotropis macrocarpa* | 2.4 |
| *Nanhaia speciosa* | 10.2 |
| *Indigofera tinctoria* | 18.6 |
| *Cullen corylifolium* | 6.0 |
| *Dalbergia yunnanensis* | 123.3 |
| *Dalbergia cultrata* | 33.0 |
| *Dalbergia cochinchinensis* | 17.2 |
| *Dalbergia sissoo* | 31.6 |
| *Dalbergia nigra* | 35.5 |
| *Kotschya aeschynomenoides* | 25.4 |
| *Stylosanthes guianensis* | 3.7 |
| *Stylosanthes viscosa* | 18.3 |
| *Arachis hypogaea* | 148.0 |
| *Pterocarpus pedatus* | 40.1 |
| *Sophora moorcroftiana* | 590.4 |
| *Sophora alopecuroides* | 8.0 |
| *Sophora tonkinensis* | 6.2 |
| *Maackia floribunda* | 12.1 |
| *Lupinus westianus* | 18.6 |
| *Lupinus luteus* | 24.1 |
| *Crotalaria pallida* | 10.3 |
| *Podalyria calyptrata* | 3.4 |
| *Ormosia hosiei* | 13.3 |
| *Cladrastis yungchunii* | 8.9 |
| *Styphnolobium japonicum* | 8.4 |
| *Vachellia nilotica* | 631.4 |
| *Acacia ligulata* | 11.8 |
| *Acacia dealbata* | 62.7 |
| *Senegalia senegal* | 2.5 |
| *Mimosa pudica* | 43.4 |
| *Xylia xylocarpa* | 19.7 |
| *Dichrostachys cinerea* | 6.9 |
| *Stryphnodendron adstringens* | 24.4 |
| *Leucaena trichandra* | 11.0 |
| *Adenanthera microsperma* | 20.4 |
| *Albizia odoratissima* | 15.8 |
| *Albizia julibrissin* | 5.4 |
| *Pararchidendron pruinosum* | 18.9 |
| *Archidendron lucyi* | 7.8 |
| *Faidherbia albida* | 8.8 |
| *Inga leiocalycina* | 6.9 |
| *Balsamocarpon brevifolium* | 21.7 |
| *Erythrostemon gilliesii* | 29.1 |
| *Biancaea sappan* | 10.4 |
| *Senna occidentalis* | 7.9 |
| *Haematoxylum brasiletto* | 5.9 |
| *Colvillea racemosa* | 10.7 |
| *Piliostigma thonningii* | 23.7 |
| *Tylosema fassoglense* | 27.8 |
| *Bauhinia brachycarpa* | 16.1 |
| *Barklya syringifolia* | 12.5 |
| *Schnella trichosepala* | 14.0 |
| *Adenolobus garipensis* | 32.8 |
| *Griffonia simplicifolia* | 17.9 |
| *Daniellia pilosa* | 32.0 |
| *Guibourtia leonensis* | 16.7 |
| *Saraca indica* | 26.0 |
| *Crudia harmsiana* | 29.9 |
| *Schotia brachypetala* | 15.3 |
| *Afzelia xylocarpa* | 12.6 |
| *Zenia insignis* | 16.6 |
| *Distemonanthus benthamianus* | 14.6 |
| *Ceratonia siliqua* | 44.3 |
| *Polygala karensium* | 29.9 |
| *Polygala tenuifolia* | 10.8 |
| *Polygala fallax* | 37.0 |
| *Polygala arillata* | 16.0 |
| *Salomonia cantoniensis* | 21.8 |
| *Epirixanthes elongata* | 2.8 |
| *Passiflora cerradensis* | 20.1 |
| *Passiflora filipes* | 17.3 |
| *Passiflora obovata* | 16.6 |
| *Passiflora contracta* | 27.2 |
| *Passiflora arbelaezii* | 16.8 |
| *Passiflora jatunsachensis* | 15.5 |
| *Passiflora auriculata* | 9.5 |
| *Passiflora pittieri* | 11.6 |
| *Passiflora microstipula* | 9.4 |
| *Passiflora affinis* | 16.7 |
| *Passiflora cincinnata* | 9.6 |
| *Passiflora rufa* | 6.5 |
| *Passiflora actinia* | 9.1 |
| *Passiflora foetida* | 10.9 |
| *Passiflora tetrandra* | 7.6 |
| *Passiflora serratifolia* | 7.1 |
| *Passiflora menispermifolia* | 132.9 |
| *Passiflora mucronata* | 4.3 |
| *Passiflora watsoniana* | 14.9 |
| *Passiflora tenuiloba* | 1.7 |
| *Passiflora cristalina* | 2.4 |
| *Passiflora recurva* | 4.7 |
| *Adenia mannii* | 25.9 |
| *Dilkea retusa* | 7.9 |
| *Vernicia montana* | 29.3 |
| *Vernicia fordii* | 22.4 |
| *Deutzianthus tonkinensis* | 19.1 |
| *Jatropha curcas* | 15.2 |
| *Manihot esculenta* | 22.2 |
| *Croton tiglium* | 22.3 |
| *Hevea brasiliensis* | 12.3 |
| *Euphorbia lathyris* | 24.3 |
| *Euphorbia pekinensis* | 13.4 |
| *Euphorbia esula* | 20.3 |
| *Euphorbia peplus* | 15.8 |
| *Euphorbia ebracteolata* | 3.2 |
| *Euphorbia hirta* | 13.3 |
| *Euphorbia maculata* | 11.6 |
| *Euphorbia tirucalli* | 23.6 |
| *Balakata baccata* | 22.2 |
| *Mallotus peltatus* | 28.1 |
| *Mallotus paniculatus* | 8.3 |
| *Ricinus communis* | 17.0 |
| *Plukenetia volubilis* | 31.5 |
| *Populus ilicifolia* | 90.7 |
| *Populus fremontii* | 22.3 |
| *Populus cathayana* | 4.6 |
| *Populus tremula* | 22.5 |
| *Salix wilsonii* | 18.8 |
| *Salix tetrasperma* | 6.8 |
| *Salix minjiangensis* | 55.4 |
| *Salix interior* | 6.2 |
| *Salix gordejevii* | 6.1 |
| *Itoa orientalis* | 27.2 |
| *Idesia polycarpa* | 22.9 |
| *Poliothyrsis sinensis* | 13.5 |
| *Dianyuea turbinata* | 34.4 |
| *Homalium stenophyllum* | 18.3 |
| *Banara guianensis* | 8.0 |
| *Grangeria borbonica* | 42.3 |
| *Licania canescens* | 17.5 |
| *Licania heteromorpha* | 4.4 |
| *Hirtella zanzibarica* | 12.4 |
| *Kostermanthus robustus* | 7.9 |
| *Dactyladenia bellayana* | 24.0 |
| *Magnistipula butayei* | 5.8 |
| *Chrysobalanus icaco* | 7.2 |
| *Phyllanthus amarus* | 41.3 |
| *Phyllanthus emblica* | 6.7 |
| *Flueggea virosa* | 9.0 |
| *Breynia fruticosa* | 8.2 |
| *Glochidion chodoense* | 6.0 |
| *Sauropus spatulifolius* | 1.4 |
| *Leptopus cordifolius* | 46.5 |
| *Baccaurea ramiflora* | 33.0 |
| *Linum lewisii* | 30.7 |
| *Linum usitatissimum* | 24.6 |
| *Linum grandiflorum* | 18.2 |
| *Linum narbonense* | 14.3 |
| *Bruguiera gymnorhiza* | 53.6 |
| *Kandelia obovata* | 29.3 |
| *Rhizophora apiculata* | 11.0 |
| *Pellacalyx yunnanensis* | 15.8 |
| *Viola phalacrocarpa* | 18.2 |
| *Garcinia paucinervis* | 11.7 |
| *Garcinia oblongifolia* | 26.9 |
| *Garcinia pedunculata* | 7.8 |
| *Garcinia mangostana* | 4.3 |
| *Garcinia gummi-gutta* | 5.8 |
| *Erythroxylum novogranatense* | 39.0 |
| *Hydnocarpus hainanensis* | 50.8 |
| *Galphimia angustifolia* | 16.1 |
| *Bunchosia argentea* | 10.3 |
| *Ctenolophon englerianus* | 44.8 |
| *Quercus spinosa* | 114.2 |
| *Quercus acuta* | 193.4 |
| *Castanea sativa* | 38.7 |
| *Trigonobalanus doichangensis* | 66.4 |
| *Castanopsis mekongensis* | 69.2 |
| *Alnus rubra* | 7.3 |
| *Alnus alnobetula* | 7.5 |
| *Alnus nitida* | 7.9 |
| *Alnus cremastogyne* | 7.8 |
| *Alnus subcordata* | 12.4 |
| *Carpinus rupestris* | 15.6 |
| *Carya sinensis* | 46.4 |
| *Carya kweichowensis* | 9.6 |
| *Carya hunanensis* | 84.1 |
| *Juglans nigra* | 38.0 |
| *Platycarya strobilacea* | 11.1 |
| *Rhoiptelea chiliantha* | 20.5 |
| *Casuarina cunninghamiana* | 14.9 |
| *Morella cerifera* | 10.2 |
| *Trichosanthes tubiflora* | 19.8 |
| *Trichosanthes truncata* | 25.6 |
| *Trichosanthes homophylla* | 18.6 |
| *Nothoalsomitra suberosa* | 58.4 |
| *Cyclanthera pedata* | 18.2 |
| *Linnaeosicyos amara* | 19.0 |
| *Hodgsonia macrocarpa* | 18.7 |
| *Hodgsonia heteroclita* | 9.3 |
| *Sechium edule* | 26.1 |
| *Gynostemma microspermum* | 25.3 |
| *Gynostemma pentagynum* | 27.7 |
| *Gynostemma cardiospermum* | 39.7 |
| *Hemsleya zhejiangensis* | 14.8 |
| *Hemsleya lijiangensis* | 6.5 |
| *Cucumis sativus* | 45.5 |
| *Cucumis melo* | 4.9 |
| *Citrullus naudinianus* | 12.4 |
| *Benincasa hispida* | 12.0 |
| *Coccinia grandis* | 10.3 |
| *Cyclantheropsis parviflora* | 38.7 |
| *Cucurbita ficifolia* | 8.5 |
| *Cucurbita pepo* | 13.2 |
| *Cionosicys macranthus* | 14.8 |
| *Momordica charantia* | 19.7 |
| *Momordica sessilifolia* | 14.5 |
| *Gerrardanthus macrorhizus* | 22.5 |
| *Dendrosicyos socotranus* | 10.1 |
| *Indofevillea khasiana* | 15.4 |
| *Herpetospermum pedunculosum* | 10.6 |
| *Thladiantha dubia* | 13.2 |
| *Bryonia marmorata* | 13.3 |
| *Siraitia grosvenorii* | 16.7 |
| *Ampelosycios humblotii* | 6.6 |
| *Begonia pulchrifolia* | 9.0 |
| *Begonia coptidifolia* | 18.9 |
| *Begonia versicolor* | 18.6 |
| *Corynocarpus laevigatus* | 39.1 |
| *Zygophyllum fabago* | 35.9 |
| *Zygophyllum xanthoxylon* | 25.5 |
| *Tetraena mongolica* | 28.5 |
| *Guaiacum angustifolium* | 30.4 |
| *Larrea tridentata* | 27.3 |
| *Tribulus terrestris* | 30.9 |
| *Krameria bicolor* | 23.7 |
| *Oxalis corniculata* | 35.1 |
| *Oxalis drummondii* | 7.9 |
| *Averrhoa carambola* | 27.8 |
| *Elaeocarpus japonicus* | 23.9 |
| *Parnassia trinervis* | 34.1 |
| *Parnassia palustris* | 18.7 |
| *Euonymus phellomanus* | 10.7 |
| *Euonymus maackii* | 5.2 |
| *Salacia amplifolia* | 15.2 |
| *Maytenus guangxiensis* | 12.8 |
| *Eucalyptus curtisii* | 1322.1 |
| *Eucalyptus erythrocorys* | 1778.9 |
| *Eucalyptus patens* | 3176.4 |
| *Eucalyptus guilfoylei* | 2016.3 |
| *Eucalyptus microcorys* | 1484.8 |
| *Eucalyptus salmonophloia* | 276.8 |
| *Eucalyptus behriana* | 6755.0 |
| *Eucalyptus fasciculosa* | 346.8 |
| *Eucalyptus pauciflora* | 2413.3 |
| *Eucalyptus torquata* | 1178.4 |
| *Corymbia tessellaris* | 490.6 |
| *Stockwellia quadrifida* | 1101.9 |
| *Allosyncarpia ternata* | 865.2 |
| *Syzygium nervosum* | 855.5 |
| *Syzygium malaccense* | 87.6 |
| *Syzygium aromaticum* | 1432.3 |
| *Plinia trunciflora* | 206.9 |
| *Psidium guajava* | 233.4 |
| *Rhodomyrtus tomentosa* | 852.0 |
| *Eugenia brasiliensis* | 19.4 |
| *Heteropyxis natalensis* | 330.9 |
| *Duabanga grandiflora* | 1501.7 |
| *Trapa maximowiczii* | 45.6 |
| *Lagerstroemia villosa* | 22.5 |
| *Cuphea hyssopifolia* | 49.1 |
| *Lythrum salicaria* | 32.1 |
| *Woodfordia fruticosa* | 904.9 |
| *Sonneratia alba* | 36.9 |
| *Punica granatum* | 60.3 |
| *Heimia apetala* | 35.1 |
| *Salpinga maranonensis* | 179.5 |
| *Opisthocentra clidemioides* | 34.3 |
| *Phyllagathis hainanensis* | 17.2 |
| *Nepsera aquatica* | 45.4 |
| *Heterocentron elegans* | 44.9 |
| *Chaetogastra longifolia* | 47.4 |
| *Graffenrieda moritziana* | 58.6 |
| *Triolena amazonica* | 34.6 |
| *Rhexia virginica* | 49.8 |
| *Microlicia cogniauxiana* | 20.6 |
| *Rhynchanthera bracteata* | 27.4 |
| *Bertolonia acuminata* | 35.0 |
| *Merianthera pulchra* | 137.4 |
| *Henriettea barkeri* | 23.1 |
| *Blakea schlimii* | 26.8 |
| *Allomaieta villosa* | 34.2 |
| *Medinilla magnifica* | 20.3 |
| *Memecylon pauciflorum* | 104.2 |
| *Tigridiopalma longmenensis* | 169.4 |
| *Tigridiopalma magnifica* | 124.8 |
| *Pterogastra divaricata* | 12.8 |
| *Eriocnema fulva* | 35.8 |
| *Ludwigia octovalvis* | 231.3 |
| *Oenothera argillicola* | 13.9 |
| *Chamaenerion angustifolium* | 108.3 |
| *Korupodendron songweanum* | 152.4 |
| *Erisma bracteosum* | 74.6 |
| *Callisthene erythroclada* | 150.1 |
| *Ruizterania albiflora* | 119.7 |
| *Qualea grandiflora* | 59.6 |
| *Vochysia acuminata* | 47.4 |
| *Terminalia phillyreifolia* | 20.0 |
| *Terminalia guyanensis* | 30.4 |
| *Terminalia neotaliala* | 16.4 |
| *Terminalia chebula* | 80.6 |
| *Combretum kraussii* | 53.0 |
| *Laguncularia racemosa* | 76.7 |
| *Quisqualis littorea* | 10.9 |
| *Saltera sarcocolla* | 216.4 |
| *Solms-laubachia calcicola* | 15.1 |
| *Solms-laubachia mieheorum* | 2.9 |
| *Solms-laubachia villosa* | 3.6 |
| *Lepidostemon rosularis* | 6.1 |
| *Dilophia salsa* | 6.8 |
| *Braya scharnhorstii* | 8.6 |
| *Braya humilis* | 6.8 |
| *Dichasianthus subtilissimus* | 5.3 |
| *Christolea crassifolia* | 15.1 |
| *Cryptospora falcata* | 3.5 |
| *Tetracme recurvata* | 9.9 |
| *Atelanthera perpusilla* | 95.5 |
| *Neotorularia torulosa* | 2.6 |
| *Pycnoplinthus uniflorus* | 2.9 |
| *Strigosella strigosa* | 3.7 |
| *Ricotia isatoides* | 19.5 |
| *Ricotia lunaria* | 17.5 |
| *Ricotia aucheri* | 14.0 |
| *Ricotia davisiana* | 8.8 |
| *Biscutella baetica* | 4.2 |
| *Biscutella vincentina* | 9.3 |
| *Biscutella lyrata* | 3.3 |
| *Lunaria rediviva* | 7.0 |
| *Lunaria annua* | 3.3 |
| *Heldreichia bupleurifolia* | 6.4 |
| *Megadenia pygmaea* | 4.1 |
| *Hemilophia sessilifolia* | 27.6 |
| *Chamira circaeoides* | 18.1 |
| *Idahoa scapigera* | 43.2 |
| *Dipoma iberideum* | 12.2 |
| *Asperuginoides axillaris* | 6.5 |
| *Ochthodium aegyptiacum* | 2.8 |
| *Crambe kralikii* | 5.8 |
| *Orychophragmus diffusus* | 4.9 |
| *Brassica oleracea* | 18.2 |
| *Sinalliaria limprichtiana* | 7.3 |
| *Henophyton deserti* | 3.3 |
| *Cremolobus peruvianus* | 20.6 |
| *Menonvillea linearis* | 3.5 |
| *Diptychocarpus strictus* | 19.9 |
| *Chorispora tenella* | 11.5 |
| *Heliophila pusilla* | 12.3 |
| *Heliophila juncea* | 12.3 |
| *Heliophila longifolia* | 4.3 |
| *Heliophila amplexicaulis* | 6.2 |
| *Aphragmus nepalensis* | 24.4 |
| *Aphragmus involucratus* | 9.3 |
| *Draba nemorosa* | 7.5 |
| *Draba oreades* | 0.3 |
| *Draba verna* | 9.8 |
| *Arabis alpina* | 5.0 |
| *Aethionema grandiflorum* | 15.7 |
| *Moriera spinosa* | 11.8 |
| *Cardamine glanduligera* | 12.8 |
| *Nasturtium officinale* | 6.2 |
| *Iodanthus pinnatifidus* | 5.0 |
| *Aurinia corymbosa* | 7.7 |
| *Meniocus linifolius* | 0.8 |
| *Matthiola alyssifolia* | 10.3 |
| *Lepidium perfoliatum* | 8.5 |
| *Lepidium echinatum* | 4.1 |
| *Delpinophytum patagonicum* | 4.2 |
| *Notoceras bicorne* | 5.1 |
| *Eutrema yunnanense* | 12.7 |
| *Mostacillastrum orbignyanum* | 28.0 |
| *Hornungia petraea* | 5.0 |
| *Arabidopsis cebennensis* | 6.0 |
| *Asta schaffneri* | 12.1 |
| *Mancoa hispida* | 6.9 |
| *Macropodium nivale* | 7.4 |
| *Microthlaspi perfoliatum* | 7.9 |
| *Dontostemon micranthus* | 4.4 |
| *Ladakiella klimesii* | 3.8 |
| *Notothlaspi australe* | 5.8 |
| *Smelowskia integrifolia* | 2.9 |
| *Iberis amara* | 3.9 |
| *Myagrum perfoliatum* | 1.0 |
| *Capparis spinosa* | 37.6 |
| *Maerua crassifolia* | 3.9 |
| *Crateva tapia* | 13.7 |
| *Cadaba glandulosa* | 3.6 |
| *Cadaba farinosa* | 8.3 |
| *Azima tetracantha* | 19.2 |
| *Salvadora persica* | 22.5 |
| *Ochradenus baccatus* | 20.5 |
| *Caylusea hexagyna* | 21.8 |
| *Cleomella lutea* | 22.4 |
| *Tarenaya hassleriana* | 6.2 |
| *Carica papaya* | 22.1 |
| *Tropaeolum pentaphyllum* | 30.7 |
| *Pentadiplandra brazzeana* | 17.3 |
| *Bretschneidera sinensis* | 8.9 |
| *Moringa oleifera* | 48.2 |
| *Acer distylum* | 63.0 |
| *Acer laevigatum* | 18.3 |
| *Acer mandshuricum* | 10.5 |
| *Acer crataegifolium* | 10.4 |
| *Acer pycnanthum* | 13.1 |
| *Acer tataricum* | 9.1 |
| *Acer pectinatum* | 10.5 |
| *Dipteronia sinensis* | 13.9 |
| *Dipteronia dyeriana* | 8.4 |
| *Handeliodendron bodinieri* | 23.0 |
| *Eurycorymbus cavaleriei* | 37.5 |
| *Dodonaea viscosa* | 23.2 |
| *Xanthoceras sorbifolium* | 35.5 |
| *Pometia tomentosa* | 11.4 |
| *Dimocarpus longan* | 1.3 |
| *Zanthoxylum madagascariense* | 6.8 |
| *Zanthoxylum asiaticum* | 2.1 |
| *Zanthoxylum schinifolium* | 5.3 |
| *Casimiroa edulis* | 19.7 |
| *Melicope pteleifolia* | 20.3 |
| *Citrus aurantiifolia* | 8.3 |
| *Citrus medica* | 9.7 |
| *Citrus sinensis* | 160.7 |
| *Murraya koenigii* | 17.2 |
| *Murraya paniculata* | 2.1 |
| *Glycosmis pentaphylla* | 5.2 |
| *Clausena excavata* | 16.4 |
| *Ruta graveolens* | 33.1 |
| *Tetradium ruticarpum* | 11.3 |
| *Orixa japonica* | 12.8 |
| *Micromelum minutum* | 9.2 |
| *Azadirachta indica* | 29.7 |
| *Khaya senegalensis* | 4.1 |
| *Entandrophragma cylindricum* | 10.7 |
| *Entandrophragma caudatum* | 3.1 |
| *Heynea velutina* | 7.6 |
| *Melia azedarach* | 6.8 |
| *Carapa guianensis* | 7.4 |
| *Aphanamixis polystachya* | 7.8 |
| *Mangifera indica* | 6.3 |
| *Rhus chinensis* | 10.7 |
| *Rhus typhina* | 2.8 |
| *Toxicodendron griffithii* | 112.8 |
| *Toxicodendron vernicifluum* | 3.9 |
| *Anacardium occidentale* | 16.7 |
| *Astronium urundeuva* | 5.3 |
| *Sclerocarya birrea* | 8.7 |
| *Lannea coromandelica* | 6.6 |
| *Commiphora gileadensis* | 99.4 |
| *Commiphora foliacea* | 21.9 |
| *Canarium album* | 11.1 |
| *Peganum harmala* | 12.5 |
| *Leitneria floridana* | 23.3 |
| *Ailanthus altissimus* | 11.2 |
| *Gossypium herbaceum* | 731.2 |
| *Gossypium barbadense* | 54.3 |
| *Gossypium longicalyx* | 18.1 |
| *Gossypium turneri* | 74.4 |
| *Gossypium robinsonii* | 4.6 |
| *Hibiscus syriacus* | 5.6 |
| *Sida szechuensis* | 5.8 |
| *Heritiera javanica* | 19.8 |
| *Heritiera fomes* | 25.9 |
| *Firmiana colorata* | 20.4 |
| *Durio zibethinus* | 34.9 |
| *Reevesia botingensis* | 11.8 |
| *Corchorus capsularis* | 13.7 |
| *Grewia biloba* | 11.3 |
| *Grewia chungii* | 19.1 |
| *Colona floribunda* | 7.0 |
| *Pterospermum menglunense* | 7.4 |
| *Craigia yunnanensis* | 19.5 |
| *Wikstroemia chamaedaphne* | 14.6 |
| *Wikstroemia indica* | 27.4 |
| *Wikstroemia dolichantha* | 11.0 |
| *Wikstroemia capitata* | 6.6 |
| *Wikstroemia scytophylla* | 9.3 |
| *Daphne genkwa* | 14.2 |
| *Daphne kiusiana* | 4.5 |
| *Daphne giraldii* | 138.5 |
| *Daphne retusa* | 19.8 |
| *Gonystylus affinis* | 27.3 |
| *Pimelea aquilonia* | 37.1 |
| *Phaleria macrocarpa* | 10.6 |
| *Diarthron linifolium* | 0.6 |
| *Shorea zeylanica* | 21.2 |
| *Shorea pachyphylla* | 9.1 |
| *Dipterocarpus turbinatus* | 11.8 |
| *Hopea hainanensis* | 20.0 |
| *Muntingia calabura* | 38.8 |
| *Bixa orellana* | 32.3 |
| *Pelargonium myrrhifolium* | 31.1 |
| *Pelargonium transvaalense* | 21.9 |
| *Pelargonium x hortorum* | 22.6 |
| *Pelargonium trifidum* | 13.8 |
| *Pelargonium alternans* | 8.3 |
| *Erodium carvifolium* | 69.5 |
| *Erodium absinthoides* | 84.8 |
| *Erodium chrysanthum* | 10.8 |
| *Monsonia marlothii* | 28.3 |
| *Monsonia emarginata* | 24.7 |
| *Hypseocharis bilobata* | 33.3 |
| *Geranium sibiricum* | 24.9 |
| *Viviania marifolia* | 42.9 |
| *Euscaphis japonica* | 27.2 |
| *Dipentodon sinicus* | 26.9 |
| *Tapiscia sinensis* | 24.4 |
| *Vitis ficifolia* | 50.6 |
| *Vitis davidii* | 1.1 |
| *Vitis mustangensis* | 84.1 |
| *Vitis bloodworthiana* | 18.2 |
| *Vitis betulifolia* | 5.9 |
| *Hypochaeris radicata* | 69705.4 |
| *Ixeris polycephala* | 1132.6 |
| *Ixeris repens* | 107.5 |
| *Taraxacum platycarpum* | 99.6 |
| *Crepidiastrum sonchifolium* | 483.6 |
| *Lactuca raddeana* | 874.7 |
| *Lactuca sativa* | 321.4 |
| *Sonchus boulosii* | 134.8 |
| *Sonchus webbii* | 624.4 |
| *Reichardia ligulata* | 671.7 |
| *Cichorium intybus* | 284.2 |
| *Bidens schimperi* | 746.3 |
| *Bidens alba* | 60.0 |
| *Bidens hillebrandiana* | 199.4 |
| *Cosmos bipinnatus* | 114.6 |
| *Ambrosia trifida* | 739.8 |
| *Aldama anchusifolia* | 40.3 |
| *Parthenium argentatum* | 589.3 |
| *Guizotia abyssinica* | 354.1 |
| *Praxelis clematidea* | 331.3 |
| *Mikania micrantha* | 36.2 |
| *Ageratina adenophora* | 122.6 |
| *Chromolaena odorata* | 76.9 |
| *Marshallia obovata* | 39.8 |
| *Tagetes erecta* | 50.4 |
| *Achyrachaena mollis* | 113.3 |
| *Crossostephium chinense* | 68.7 |
| *Stilpnolepis centiflora* | 16.6 |
| *Soliva sessilis* | 109.7 |
| *Tanacetum cinerariifolium* | 19.1 |
| *Diplostephium azureum* | 1127.4 |
| *Diplostephium oxapampanum* | 78.7 |
| *Exostigma notobellidiastrum* | 56.1 |
| *Archibaccharis asperifolia* | 258.6 |
| *Linochilus costaricensis* | 18.4 |
| *Symphyotrichum subulatum* | 24.2 |
| *Eschenbachia blinii* | 234.5 |
| *Dendrosenecio meruensis* | 292.4 |
| *Farfugium japonicum* | 35.0 |
| *Petasites japonicus* | 15.5 |
| *Pluchea indica* | 81.1 |
| *Saussurea polylepis* | 436.1 |
| *Saussurea tsoongii* | 328.8 |
| *Tugarinovia mongolica* | 115.6 |
| *Cirsium setosum* | 201.3 |
| *Doniophyton anomalum* | 88.0 |
| *Gerbera jamesonii* | 141.0 |
| *Gymnanthemum amygdalinum* | 135.6 |
| *Nymphoides coronata* | 98.8 |
| *Menyanthes trifoliata* | 32.5 |
| *Adenophora divaricata* | 24.2 |
| *Adenophora remotiflora* | 8.8 |
| *Campanula zangezura* | 8.2 |
| *Campanula takesimana* | 10.2 |
| *Platycodon grandiflorus* | 33.5 |
| *Leptocodon hirsutus* | 15.4 |
| *Trachelium caeruleum* | 11.9 |
| *Codonopsis tsinlingensis* | 8.5 |
| *Viburnum brachybotryum* | 661.0 |
| *Viburnum betulifolium* | 14.1 |
| *Sambucus nigra* | 7.6 |
| *Lonicera nervosa* | 5.6 |
| *Patrinia saniculifolia* | 10.6 |
| *Acanthocalyx alba* | 10.8 |
| *Triosteum pinnatifidum* | 28.0 |
| *Pterocephalus hookeri* | 9.1 |
| *Dipsacus japonicus* | 18.8 |
| *Nardostachys jatamansi* | 12.3 |
| *Heptacodium miconioides* | 19.6 |
| *Scabiosa tschiliensis* | 11.9 |
| *Valeriana officinalis* | 16.8 |
| *Abelia x grandiflora* | 4.0 |
| *Morina longifolia* | 7.9 |
| *Weigela florida* | 17.6 |
| *Triplostegia glandulifera* | 9.2 |
| *Angelica morii* | 12.7 |
| *Peucedanum insolens* | 6.3 |
| *Peucedanum terebinthaceum* | 5.0 |
| *Glehnia littoralis* | 11.1 |
| *Heracleum candicans* | 14.9 |
| *Carum carvi* | 14.2 |
| *Crithmum maritimum* | 15.6 |
| *Anethum foeniculum* | 11.1 |
| *Prangos trifida* | 5.4 |
| *Coriandrum sativum* | 5.2 |
| *Bupleurum chinense* | 14.6 |
| *Ligusticum tenuissimum* | 9.6 |
| *Meeboldia yunnanensis* | 5.8 |
| *Pterygopleurum neurophyllum* | 9.9 |
| *Pternopetalum davidii* | 2.5 |
| *Ferula sinkiangensis* | 12.6 |
| *Cuminum cyminum* | 6.2 |
| *Anthriscus cerefolium* | 7.3 |
| *Pleurospermum camtschaticum* | 18.7 |
| *Haplosphaera himalayensis* | 6.2 |
| *Heteromorpha arborescens* | 6.9 |
| *Cicuta virosa* | 2.6 |
| *Changium smyrnioides* | 6.1 |
| *Panax trifolius* | 12.3 |
| *Panax ginseng* | 31.6 |
| *Hydrocotyle sibthorpioides* | 12.1 |
| *Brassaiopsis hainla* | 7.6 |
| *Pennantia cunninghamii* | 9.5 |
| *Ilex x attenuata* | 31.6 |
| *Gonocaryum lobbianum* | 94.5 |
| *Aphyllon californicum* | 64.2 |
| *Aphyllon fasciculatum* | 18.4 |
| *Orobanche crenata* | 26.0 |
| *Orobanche gracilis* | 9.4 |
| *Orobanche pancicii* | 10.5 |
| *Orobanche densiflora* | 4.2 |
| *Orobanche rapum-genistae* | 7.8 |
| *Cistanche deserticola* | 42.6 |
| *Phelipanche ramosa* | 14.8 |
| *Epifagus virginiana* | 2.9 |
| *Lathraea squamaria* | 65.8 |
| *Euphrasia regelii* | 22.8 |
| *Melampyrum roseum* | 31.7 |
| *Melampyrum koreanum* | 2.9 |
| *Schwalbea americana* | 29.3 |
| *Siphonostegia chinensis* | 13.5 |
| *Phtheirospermum japonicum* | 3.9 |
| *Pedicularis oederi* | 12.2 |
| *Pedicularis longiflora* | 69.1 |
| *Pedicularis muscicola* | 16.3 |
| *Pedicularis ishidoyana* | 9.3 |
| *Pedicularis hallaisanensis* | 6.9 |
| *Triphysaria versicolor* | 7.5 |
| *Lindenbergia philippensis* | 9.9 |
| *Triaenophora shennongjiaensis* | 12.0 |
| *Salvia yunnanensis* | 6.6 |
| *Salvia japonica* | 9.1 |
| *Salvia rosmarinus* | 7.4 |
| *Nepeta tenuifolia* | 7.8 |
| *Prunella vulgaris* | 3.8 |
| *Clinopodium abyssinicum* | 2.6 |
| *Coleus xanthanthus* | 4.6 |
| *Lavandula dentata* | 5.1 |
| *Hanceola exserta* | 6.1 |
| *Elsholtzia densa* | 7.0 |
| *Teucrium mascatense* | 48.8 |
| *Ajuga forrestii* | 5.6 |
| *Clerodendrum japonicum* | 3.3 |
| *Pogostemon cablin* | 15.6 |
| *Pogostemon yatabeanus* | 4.5 |
| *Leucosceptrum canum* | 11.4 |
| *Eriophyton wallichii* | 4.4 |
| *Dasymalla teckiana* | 15.7 |
| *Dicrastylis parvifolia* | 28.3 |
| *Premna microphylla* | 15.7 |
| *Gmelina hainanensis* | 6.7 |
| *Holmskioldia sanguinea* | 3.3 |
| *Congea tomentosa* | 13.0 |
| *Tectona grandis* | 6.1 |
| *Callicarpa formosana* | 2052.1 |
| *Clerodendranthus spicatus* | 2.6 |
| *Adenocalymma cristicalyx* | 1.5 |
| *Adenocalymma peregrinum* | 75.6 |
| *Amphilophium dusenianum* | 15.1 |
| *Amphilophium steyermarkii* | 5.0 |
| *Anemopaegma chamberlaynii* | 8.2 |
| *Dolichandra cynanchoides* | 4.8 |
| *Tanaecium tetragonolobum* | 9.8 |
| *Neojobertia candolleana* | 2.2 |
| *Incarvillea compacta* | 26.6 |
| *Incarvillea sinensis* | 12.9 |
| *Incarvillea arguta* | 6.8 |
| *Tecomaria capensis* | 6.6 |
| *Dolichandrone spathacea* | 17.9 |
| *Spathodea campanulata* | 5.9 |
| *Oroxylum indicum* | 13.0 |
| *Syringa oblata* | 4.9 |
| *Chionanthus macrobotrys* | 12.6 |
| *Chionanthus maxwellii* | 1.1 |
| *Chionanthus panamensis* | 2.1 |
| *Ligustrum quihoui* | 3.6 |
| *Osmanthus aff. armatus Besnard 02-2013* | 6.2 |
| *Olea europaea* | 10.7 |
| *Jasminum nudiflorum* | 16.8 |
| *Jasminum fluminense* | 9.1 |
| *Chrysojasminum fruticans* | 18.2 |
| *Nyctanthes arbor-tristis* | 17.9 |
| *Justicia leptostachya* | 13.9 |
| *Justicia flava* | 13.3 |
| *Justicia adhatoda* | 10.3 |
| *Peristrophe japonica* | 4.8 |
| *Pseuderanthemum haikangense* | 5.9 |
| *Clinacanthus nutans* | 3.7 |
| *Echinacanthus attenuatus* | 16.7 |
| *Echinacanthus lofouensis* | 10.7 |
| *Echinacanthus longzhouensis* | 28.0 |
| *Strobilanthes medahinnensis* | 5.9 |
| *Aphelandra knappiae* | 26.7 |
| *Acanthus ilicifolius* | 78.1 |
| *Blepharis ciliaris* | 6.3 |
| *Andrographis paniculata* | 11.5 |
| *Avicennia marina* | 16.7 |
| *Utricularia reniformis* | 63.5 |
| *Utricularia gibba* | 104.2 |
| *Genlisea violacea* | 17.3 |
| *Genlisea margaretae* | 13.5 |
| *Pinguicula ehlersiae* | 23.6 |
| *Pinguicula alpina* | 11.9 |
| *Primulina ophiopogoides* | 13.5 |
| *Primulina linearifolia* | 5.0 |
| *Primulina liboensis* | 4.6 |
| *Oreocharis esquirolii* | 13.6 |
| *Oreocharis cotinifolia* | 10.0 |
| *Petrocodon jingxiensis* | 10.2 |
| *Lysionotus pauciflorus* | 4.9 |
| *Hemiboea ovalifolia* | 2.2 |
| *Paraboea glutinosa* | 8.5 |
| *Paraboea dolomitica* | 10.1 |
| *Paraboea guilinensis* | 8.3 |
| *Paraboea clavisepala* | 1.6 |
| *Corallodiscus flabellatus* | 11.8 |
| *Streptocarpus teitensis* | 4.7 |
| *Haberlea rhodopensis* | 8.5 |
| *Achimenes erecta* | 204.8 |
| *Veronica eriogyne* | 6.6 |
| *Veronicastrum axillare* | 13.2 |
| *Plantago lagopus* | 28.0 |
| *Plantago ovata* | 8.7 |
| *Hippuris vulgaris* | 14.9 |
| *Hemiphragma heterophyllum* | 14.2 |
| *Bacopa monnieri* | 63.9 |
| *Lippia origanoides* | 10.4 |
| *Duranta erecta* | 17.7 |
| *Scrophularia dentata* | 10.7 |
| *Wightia speciosissima* | 75.4 |
| *Erythranthe lutea* | 8.4 |
| *Phryma leptostachya* | 6.6 |
| *Sesamum indicum* | 1.5 |
| *Gentiana zollingeri* | 16.3 |
| *Kuepferia otophora* | 26.5 |
| *Tripterospermum membranaceum* | 11.1 |
| *Swertia cordata* | 11.3 |
| *Swertia souliei* | 13.4 |
| *Swertia tetraptera* | 6.8 |
| *Comastoma pulmonarium* | 9.4 |
| *Lomatogonium perenne* | 4.1 |
| *Exacum affine* | 30.9 |
| *Cyrtophyllum fragrans* | 11.6 |
| *Leptodermis oblonga* | 27.2 |
| *Paederia scandens* | 12.9 |
| *Gynochthodes nanlingensis* | 14.7 |
| *Morinda citrifolia* | 25.2 |
| *Oldenlandia corymbosa* | 40.4 |
| *Rubia cordifolia* | 10.0 |
| *Gardenia jasminoides* | 11.9 |
| *Coffea canephora* | 3.7 |
| *Scyphiphora hydrophyllacea* | 14.9 |
| *Emmenopterys henryi* | 8.2 |
| *Antirhea chinensis* | 8.4 |
| *Asclepias syriaca* | 4.9 |
| *Cynanchum chinense* | 5.9 |
| *Pentasachme caudatum* | 48.4 |
| *Cerbera manghas* | 23.5 |
| *Plumeria rubra* | 24.4 |
| *Carissa macrocarpa* | 13.1 |
| *Catharanthus roseus* | 11.3 |
| *Periploca forrestii* | 15.6 |
| *Hemidesmus indicus* | 2.4 |
| *Chonemorpha megacalyx* | 17.2 |
| *Apocynum venetum* | 13.8 |
| *Wrightia laevis* | 13.4 |
| *Mitrasacme pygmaea* | 42.9 |
| *Mitreola yangchunensis* | 8.5 |
| *Cuscuta exaltata* | 28.2 |
| *Cuscuta reflexa* | 18.9 |
| *Cuscuta pentagona* | 32.1 |
| *Cressa cretica* | 22.0 |
| *Evolvulus alsinoides* | 15.9 |
| *Ipomoea setifera* | 15.6 |
| *Ipomoea batatas* | 11.7 |
| *Ipomoea goyazensis* | 13.4 |
| *Ipomoea splendor-sylvae* | 16.7 |
| *Ipomoea quamoclit* | 1.4 |
| *Convolvulus arvensis* | 38.4 |
| *Solanum dasyphyllum* | 9.2 |
| *Solanum cerasiferum* | 17.3 |
| *Solanum agnewiorum* | 1.4 |
| *Solanum nigrum* | 3.8 |
| *Przewalskia tangutica* | 8.4 |
| *Atropanthe sinensis* | 3.0 |
| *Capsicum pubescens* | 16.0 |
| *Datura stramonium* | 8.6 |
| *Nicotiana amplexicaulis* | 10.4 |
| *Nicotiana otophora* | 11.1 |
| *Nicotiana tomentosiformis* | 2.3 |
| *Eucommia ulmoides* | 44.3 |
| *Aucuba eriobotryifolia* | 23.3 |
| *Arnebia euchroma* | 13.1 |
| *Lithospermum erythrorhizon* | 2.6 |
| *Onosma fuyunensis* | 5.0 |
| *Borago officinalis* | 11.3 |
| *Lennoa madreporoides* | 5.5 |
| *Pholisma arenarium* | 11.4 |
| *Iodes cirrhosa* | 108.7 |
| *Primula kwangtungensis* | 19.4 |
| *Primula handeliana* | 11.6 |
| *Primula ranunculoides* | 21.9 |
| *Primula moupinensis* | 9.5 |
| *Primula sinensis* | 17.6 |
| *Primula pulchella* | 6.1 |
| *Primula densa* | 30.5 |
| *Primula pellucida* | 9.8 |
| *Primula jiugongshanensis* | 12.2 |
| *Primula bulleyana* | 23.8 |
| *Primula denticulata* | 3.6 |
| *Primula persimilis* | 9.9 |
| *Androsace bulleyana* | 19.3 |
| *Androsace paxiana* | 13.2 |
| *Aegiceras corniculatum* | 193.4 |
| *Myrsine stolonifera* | 6.8 |
| *Impatiens davidii* | 15.3 |
| *Impatiens piufanensis* | 7.5 |
| *Impatiens cyanantha* | 8.1 |
| *Impatiens alpicola* | 24.9 |
| *Impatiens hawkeri* | 29.5 |
| *Impatiens glandulifera* | 5.8 |
| *Hydrocera triflora* | 16.6 |
| *Agapetes malipoensis* | 23.6 |
| *Gaultheria griffithiana* | 23.0 |
| *Camellia fraterna* | 265.6 |
| *Camellia impressinervis* | 37.3 |
| *Bruinsmia polysperma* | 34.0 |
| *Bruinsmia styracoides* | 6.8 |
| *Alniphyllum fortunei* | 17.4 |
| *Actinidia tetramera* | 16.0 |
| *Actinidia valvata* | 1.2 |
| *Actinidia lanceolata* | 5.1 |
| *Pouteria caimito* | 18.2 |
| *Synsepalum dulcificum* | 7.3 |
| *Chrysophyllum cainito* | 3.1 |
| *Sideroxylon wightianum* | 30.5 |
| *Diospyros hainanensis* | 17.2 |
| *Diospyros blancoi* | 12.2 |
| *Diospyros maclurei* | 9.8 |
| *Bertholletia excelsa* | 50.5 |
| *Polemonium chinense* | 20.7 |
| *Clethra delavayi* | 14.8 |
| *Cornus oblonga* | 47.6 |
| *Cornus nuttallii* | 19.8 |
| *Alangium kurzii* | 7.4 |
| *Hydrangea caerulea* | 8.7 |
| *Hydrangea petiolaris* | 12.3 |
| *Hydrangea densifolia* | 22.7 |
| *Hydrangea davidii* | 16.2 |
| *Hydrangea platyarguta* | 43.6 |
| *Hydrangea hydrangeoides* | 3.4 |
| *Hydrangea febrifuga* | 6.4 |
| *Kirengeshoma palmata* | 10.6 |
| *Whipplea modesta* | 10.0 |
| *Loasa nitida* | 31.7 |
| *Loasa nana* | 8.6 |
| *Nasa triphylla* | 20.3 |
| *Nasa urens* | 11.5 |
| *Mentzelia aspera* | 7.2 |
| *Mentzelia albicaulis* | 7.6 |
| *Eucnide grandiflora* | 11.5 |
| *Petalonyx linearis* | 10.4 |
| *Hydrostachys polymorpha* | 15.0 |
| *Hydrostachys imbricata* | 15.0 |
| *Hydrostachys goudotiana* | 13.3 |
| *Camptotheca acuminata* | 16.2 |
| *Nyssa ogeche* | 21.8 |
| *Diplopanax stachyanthus* | 13.4 |
| *Grubbia tomentosa* | 12.1 |
| *Grubbia rosmarinifolia* | 6.0 |
| *Curtisia dentata* | 7.1 |
| *Atriplex gmelinii* | 49.7 |
| *Atriplex centralasiatica* | 10.8 |
| *Chenopodium ficifolium* | 46.5 |
| *Oxybasis glauca* | 74.4 |
| *Dysphania ambrosioides* | 40.6 |
| *Suaeda glauca* | 34.2 |
| *Suaeda salsa* | 5.8 |
| *Caroxylon passerinum* | 52.0 |
| *Silene noctiflora* | 15.8 |
| *Silene conoidea* | 4.0 |
| *Silene vulgaris* | 4.0 |
| *Silene latifolia* | 3.2 |
| *Silene wilfordii* | 12.4 |
| *Silene kiusiana* | 11.7 |
| *Silene chalcedonica* | 4.3 |
| *Silene paradoxa* | 14.8 |
| *Agrostemma githago* | 8.0 |
| *Gypsophila oldhamiana* | 14.7 |
| *Gymnocarpos przewalskii* | 18.1 |
| *Rheum tanguticum* | 37.8 |
| *Rumex hastatus* | 19.8 |
| *Oxyria sinensis* | 8.7 |
| *Fagopyrum esculentum* | 23.6 |
| *Persicaria chinensis* | 23.2 |
| *Persicaria filiformis* | 2.9 |
| *Muehlenbeckia australis* | 5.1 |
| *Muehlenbeckia gunnii* | 7.7 |
| *Polygonum aviculare* | 12.0 |
| *Drosera rotundifolia* | 25.4 |
| *Drosera regia* | 15.8 |
| *Aldrovanda vesiculosa* | 20.7 |
| *Dionaea muscipula* | 18.7 |
| *Rhipsalis baccifera* | 26.2 |
| *Rhipsalis teres* | 20.0 |
| *Lophocereus schottii* | 10.9 |
| *Selenicereus undatus* | 12.4 |
| *Portulaca oleracea* | 11.8 |
| *Cistanthe longiscapa* | 9.1 |
| *Ptilotus polystachyus* | 23.7 |
| *Celosia cristata* | 22.8 |
| *Alternanthera philoxeroides* | 18.4 |
| *Limonium aureum* | 28.4 |
| *Plumbago auriculata* | 29.6 |
| *Nepenthes ventricosa x Nepenthes alata* | 29.0 |
| *Nepenthes khasiana* | 21.0 |
| *Bougainvillea pachyphylla* | 15.8 |
| *Bougainvillea peruviana* | 69.6 |
| *Nyctaginia capitata* | 8.7 |
| *Boerhavia diffusa* | 4.3 |
| *Myricaria prostrata* | 26.5 |
| *Tamarix taklamakanensis* | 14.0 |
| *Monococcus echinophorus* | 33.1 |
| *Phytolacca insularis* | 17.6 |
| *Petiveria alliacea* | 18.3 |
| *Tetragonia tetragonoides* | 6.4 |
| *Mesembryanthemum crystallinum* | 10.5 |
| *Stegnosperma halimifolium* | 16.0 |
| *Chunia bucklandioides* | 338.9 |
| *Mytilaria laosensis* | 15.6 |
| *Disanthus cercidifolius* | 14.4 |
| *Sinowilsonia henryi* | 20.6 |
| *Rhodoleia championii* | 18.7 |
| *Fortunearia sinensis* | 8.1 |
| *Loropetalum subcordatum* | 4.4 |
| *Saxifraga stolonifera* | 50.8 |
| *Saxifraga umbellulata* | 19.0 |
| *Chrysosplenium sinicum* | 20.1 |
| *Chrysosplenium alternifolium* | 10.0 |
| *Chrysosplenium aureobracteatum* | 6.1 |
| *Chrysosplenium kamtschaticum* | 6.5 |
| *Tanakaea radicans* | 23.2 |
| *Micranthes melanocentra* | 30.8 |
| *Bergenia scopulosa* | 26.6 |
| *Mitella diphylla* | 9.4 |
| *Mukdenia rossii* | 4.0 |
| *Sedum lineare* | 20.7 |
| *Sedum emarginatum* | 13.1 |
| *Sedum sarmentosum* | 7.4 |
| *Sedum oryzifolium* | 12.8 |
| *Rhodiola kirilowii* | 53.7 |
| *Rhodiola rosea* | 10.9 |
| *Rhodiola sacra* | 4.4 |
| *Crassula perforata* | 21.3 |
| *Aeonium arboreum* | 14.9 |
| *Cotyledon tomentosa* | 12.2 |
| *Orostachys fimbriata* | 12.8 |
| *Sempervivum tectorum* | 8.2 |
| *Hylotelephium verticillatum* | 4.1 |
| *Sinocrassula densirosulata* | 5.7 |
| *Paeonia suffruticosa* | 19.5 |
| *Paeonia lutea* | 24.8 |
| *Paeonia sp. CV Hwang-Moran* | 15.6 |
| *Myriophyllum aquaticum* | 11.6 |
| *Myriophyllum spicatum* | 19.7 |
| *Itea chinensis* | 28.9 |
| *Penthorum chinense* | 123.3 |
| *Daphniphyllum oldhamii* | 22.0 |
| *Cecarria obtusifolia* | 23.5 |
| *Taxillus matsudae* | 19.4 |
| *Taxillus liquidambaricola* | 4.9 |
| *Taxillus chinensis* | 3.2 |
| *Taxillus lonicerifolius* | 5.2 |
| *Scurrula notothixoides* | 12.0 |
| *Tolypanthus maclurei* | 18.0 |
| *Dendrophthoe pentandra* | 10.4 |
| *Helixanthera parasitica* | 14.0 |
| *Moquiniella rubra* | 7.6 |
| *Nuytsia floribunda* | 31.1 |
| *Macrosolen cochinchinensis* | 14.5 |
| *Elytranthe albida* | 13.4 |
| *Viscum articulatum* | 45.4 |
| *Viscum crassulae* | 23.2 |
| *Viscum liquidambaricola* | 25.9 |
| *Viscum minimum* | 23.0 |
| *Osyris alba* | 15.4 |
| *Osyris wightiana* | 9.3 |
| *Santalum album* | 28.8 |
| *Schoepfia jasminodora* | 48.5 |
| *Malania oleifera* | 47.3 |
| *Erythropalum scandens* | 43.6 |
| *Dendrotrophe varians* | 23.3 |
| *Pyrularia edulis* | 27.4 |
| *Dillenia indica* | 61.6 |
| *Dendrobium salaccense* | 1.8 |
| *Dendrobium aduncum* | 4.0 |
| *Dendrobium wilsonii* | 12.6 |
| *Dendrobium primulinum* | 6.2 |
| *Oberonioides microtatantha* | 5.3 |
| *Oberonia seidenfadenii* | 4.5 |
| *Palmorchis pabstii* | 55.4 |
| *Epipactis thunbergii* | 8.5 |
| *Neottia cordata* | 2.6 |
| *Neottia ovata* | 0.8 |
| *Cymbidium aloifolium* | 5.2 |
| *Geodorum eulophioides* | 2.4 |
| *Dipodium roseum* | 2.1 |
| *Corallorhiza maculata* | 20.5 |
| *Tainia cordifolia* | 5.1 |
| *Gastrodia elata* | 0.1 |
| *Chamaegastrodia shikokiana* | 27.4 |
| *Goodyera rosulacea* | 2.8 |
| *Goodyera schlechtendaliana* | 1.9 |
| *Ludisia discolor* | 6.5 |
| *Lankesterella ceracifolia* | 2.6 |
| *Cyclopogon longibracteatus* | 3.3 |
| *Prescottia stachyodes* | 4.0 |
| *Platanthera japonica* | 7.0 |
| *Corybas taliensis* | 7.2 |
| *Paphiopedilum niveum* | 12.5 |
| *Paphiopedilum bellatulum* | 4.3 |
| *Paphiopedilum armeniacum* | 9.6 |
| *Apostasia odorata* | 1.0 |
| *Agave virginica* | 10.7 |
| *Anemarrhena asphodeloides* | 3.7 |
| *Sansevieria conspicua* | 1.7 |
| *Asparagus racemosus* | 1.3 |
| *Cordyline indivisa* | 2.3 |
| *Milla biflora* | 1.8 |
| *Allium cyathophorum* | 5.5 |
| *Allium paradoxum* | 0.8 |
| *Allium sativum* | 1.6 |
| *Allium neriniflorum* | 2.3 |
| *Hippeastrum rutilum* | 6.5 |
| *Narcissus poeticus* | 2.5 |
| *Agapanthus coddii* | 2.5 |
| *Iris domestica* | 1.1 |
| *Iris lactea* | 3.6 |
| *Sisyrinchium angustifolium* | 3.8 |
| *Geosiris australiensis* | 13.1 |
| *Xanthorrhoea preissii* | 3.4 |
| *Barnardia japonica* | 2.0 |
| *Albuca kirkii* | 3.8 |
| *Molineria capitulata* | 0.9 |
| *Fargesia denudata* | 168.1 |
| *Neomicrocalamus prainii* | 1.2 |
| *Triticum aestivum* | 10.2 |
| *Secale cereale* | 1.0 |
| *Lolium arundinaceum* | 3.7 |
| *Lygeum spartum* | 1.7 |
| *Whiteochloa capillipes* | 2.3 |
| *Echinochloa colona* | 4.1 |
| *Oncorachis ramosa* | 3.2 |
| *Tristachya leucothrix* | 2.8 |
| *Tragus australianus* | 10.0 |
| *Elytrophorus globularis* | 2.9 |
| *Dregeochloa pumilla* | 1.2 |
| *Pharus latifolius* | 2.2 |
| *Anomochloa marantoidea* | 3.9 |
| *Carex neurocarpa* | 17.7 |
| *Isolepis setacea* | 12.3 |
| *Bolboschoenus planiculmis* | 11.4 |
| *Eriocaulon buergerianum* | 5.2 |
| *Eriocaulon decemflorum* | 5.0 |
| *Sparganium natans* | 14.0 |
| *Ananas comosus* | 5.7 |
| *Flagellaria indica* | 4.2 |
| *Zingiber montanum* | 2.6 |
| *Curcuma flaviflora* | 1.5 |
| *Pollia japonica* | 8.5 |
| *Anigozanthos flavidus* | 8.8 |
| *Podococcus barteri* | 1.4 |
| *Mauritia flexuosa* | 7.6 |
| *Burmannia cryptopetala* | 27.9 |
| *Burmannia nepalensis* | 17.1 |
| *Burmannia coelestis* | 4.3 |
| *Burmannia championii* | 1.5 |
| *Burmannia disticha* | 3.5 |
| *Dioscorea esculenta* | 2.5 |
| *Dioscorea bulbifera* | 2.1 |
| *Dioscorea schimperiana* | 2.5 |
| *Trichopus zeylanicus* | 7.3 |
| *Metanarthecium luteoviride* | 4.9 |
| *Tacca leontopetaloides* | 7.5 |
| *Lilium pardanthinum* | 3.3 |
| *Medeola virginiana* | 5.1 |
| *Calochortus uniflorus* | 2.7 |
| *Heloniopsis tubiflora* | 6.1 |
| *Iphigenia indica* | 5.9 |
| *Alstroemeria hybrid cultivar* | 4.3 |
| *Luzuriaga radicans* | 2.4 |
| *Campynema lineare* | 6.9 |
| *Stemona japonica* | 3.5 |
| *Stemona mairei* | 3.0 |
| *Croomia pauciflora* | 2.9 |
| *Xerophyta schlechteri* | 5.8 |
| *Acanthochlamys bracteata* | 5.7 |
| *Carludovica palmata* | 2.3 |
| *Ottelia alismoides* | 19.2 |
| *Ottelia cordata* | 18.9 |
| *Najas flexilis* | 13.6 |
| *Elodea canadensis* | 48.6 |
| *Halophila beccarii* | 24.9 |
| *Enhalus acoroides* | 38.1 |
| *Thalassia hemprichii* | 14.6 |
| *Ruppia brevipedunculata* | 53.6 |
| *Arisarum simorrhinum* | 7.7 |
| *Lemna minor* | 12.5 |
| *Wolffia globosa* | 9.8 |
| *Orontium aquaticum* | 7.8 |
| *Anthurium huixtlense* | 6.7 |
| *Homalomena occulta* | 9.1 |
| *Stylochaeton bogneri* | 4.0 |
| *Lasia spinosa* | 3.1 |
| *Zostera marina* | 10.3 |
| *Zostera japonica* | 6.0 |
| *Phyllospadix iwatensis* | 10.8 |
| *Stuckenia pectinata* | 20.8 |
| *Sagittaria trifolia* | 8.2 |
| *Alisma plantago-aquatica* | 3.3 |
| *Butomus umbellatus* | 9.0 |
| *Staphisagria macrosperma* | 2.2 |
| *Anemoclema glaucifolium* | 4.7 |
| *Ranunculus occidentalis* | 6.0 |
| *Nigella damascena* | 4.4 |
| *Asteropyrum peltatum* | 7.9 |
| *Callianthemum taipaicum* | 5.1 |
| *Helleborus thibetanus* | 8.6 |
| *Caltha palustris* | 4.2 |
| *Enemion raddeanum* | 13.3 |
| *Thalictrum thalictroides* | 4.0 |
| *Glaucidium palmatum* | 14.6 |
| *Coptis japonica* | 2.2 |
| *Corydalis shensiana* | 7.0 |
| *Corydalis inopinata* | 8.6 |
| *Corydalis davidii* | 7.1 |
| *Corydalis hsiaowutaishanensis* | 7.7 |
| *Corydalis adunca* | 10.0 |
| *Papaver orientale* | 2.8 |
| *Meconopsis henrici* | 3.2 |
| *Macleaya microcarpa* | 6.8 |
| *Bongardia chrysogonum* | 9.4 |
| *Epimedium sagittatum* | 7.3 |
| *Gymnospermium microrrhynchum* | 5.2 |
| *Achlys triphylla* | 9.3 |
| *Jeffersonia diphylla* | 2.1 |
| *Stephania tetrandra* | 8.9 |
| *Stephania epigaea* | 4.2 |
| *Stephania japonica* | 1.4 |
| *Sinomenium acutum* | 10.8 |
| *Tinospora cordifolia* | 5.0 |
| *Decaisnea insignis* | 10.2 |
| *Circaeaster agrestis* | 19.1 |
| *Kingdonia uniflora* | 5.3 |
| *Euptelea pleiosperma* | 5.3 |
| *Cassytha filiformis* | 10.3 |
| *Beilschmiedia brachythyrsa* | 12.6 |
| *Phoebe formosana* | 32.2 |
| *Cinnamomum verum* | 0.6 |
| *Aristolochia macrophylla* | 11.9 |
| *Aristolochia contorta* | 2.8 |
| *Saruma henryi* | 1.2 |
| *Gymnotheca chinensis* | 3.1 |
| *Annona muricata* | 8.1 |
| *Uvaria macrophylla* | 2.9 |
| *Chieniodendron hainanense* | 2.9 |
| *Greenwayodendron suaveolens* | 2.6 |
| *Meliosma oldhamii* | 3.0 |
| *Meliosma aff. cuneifolia Moore 333* | 4.1 |
| *Sabia yunnanensis* | 7.5 |
| *Nelumbo lutea* | 5.0 |
| *Grevillea robusta* | 5.2 |
| *Platanus occidentalis* | 4.1 |
| *Buxus microphylla* | 33.6 |
| *Pachysandra terminalis* | 1.9 |
| *Ceratophyllum demersum* | 27.2 |
| *Trochodendron aralioides* | 4.1 |
| *Tetracentron sinense* | 1.8 |
| *Amborella trichopoda* | 1.4 |
| *Malus kansuensis* | 32.8 |
| *Rhaphiolepis lanceolata* | 15.6 |
| *Cotoneaster horizontalis* | 6.9 |
| *Photinia serratifolia* | 28.4 |
| *Phippsiomeles matudae* | 6.7 |
| *Hesperomeles ferruginea* | 6.5 |
| *Rubus columellaris* | 10.8 |
| *Rubus pectinaris* | 4.5 |
| *Rosa canina* | 7.6 |
| *Fragaria moupinensis* | 5.8 |
| *Hovenia dulcis* | 6.5 |
| *Hippophae salicifolia* | 5.5 |
| *Lathyrus littoralis* | 2.9 |
| *Lathyrus davidii* | 8.4 |
| *Vigna unguiculata* | 3.8 |
| *Cajanus cajan* | 3.8 |
| *Dendrolobium lanceolatum* | 6.6 |
| *Dalbergia chlorocarpa* | 65.3 |
| *Dalbergia cearensis* | 4.9 |
| *Dalbergia frutescens* | 5.5 |
| *Echinosophora koreensis* | 2.9 |
| *Senegalia laeta* | 2.5 |
| *Libidibia coriaria* | 3.2 |
| *Senna spectabilis* | 11.3 |
| *Gymnocladus chinensis* | 9.5 |
| *Erythrophleum fordii* | 4.5 |
| *Cercis canadensis* | 1.1 |
| *Passiflora vitifolia* | 6.1 |
| *Passiflora deidamioides* | 10.8 |
| *Passiflora suberosa* | 3.3 |
| *Populus hopeiensis* | 8.8 |
| *Azara serrata* | 4.7 |
| *Licania sprucei* | 8.5 |
| *Licania alba* | 5.6 |
| *Licania michauxii* | 3.1 |
| *Licania majuscula* | 3.7 |
| *Dactyladenia buchneri* | 7.2 |
| *Gaulettia elata* | 2.1 |
| *Viola mirabilis* | 10.3 |
| *Quercus virginiana* | 18.8 |
| *Quercus aquifolioides* | 4.4 |
| *Fagus sylvatica* | 25.7 |
| *Castanopsis concinna* | 10.9 |
| *Carya ovata* | 5.2 |
| *Juglans hopeiensis* | 125.6 |
| *Juglans cathayensis* | 0.5 |
| *Alnus orientalis* | 5.3 |
| *Corylus jacquemontii* | 3.7 |
| *Morella salicifolia* | 9.4 |
| *Trichosanthes baviensis* | 5.1 |
| *Lagenaria siceraria* | 2.6 |
| *Baijiania yunnanensis* | 5.4 |
| *Oxalis corymbosa* | 74.4 |
| *Eucalyptus deglupta* | 12406.0 |
| *Eucalyptus radiata* | 684.9 |
| *Corymbia eximia* | 337.9 |
| *Corymbia gummifera* | 6339.2 |
| *Myrcia amethystina* | 717.9 |
| *Oenothera lindheimeri* | 52.1 |
| *Salvertia convallariodora* | 293.2 |
| *Solms-laubachia eurycarpa* | 7.6 |
| *Leiospora exscapa* | 6.7 |
| *Sinapis arvensis* | 0.4 |
| *Aethionema arabicum* | 2.1 |
| *Draba aizoides* | 6.5 |
| *Cardamine enneaphyllos* | 3.3 |
| *Barbarea verna* | 4.3 |
| *Matthiola longipetala* | 0.8 |
| *Ionopsidium acaule* | 3.0 |
| *Descurainia sophia* | 5.0 |
| *Megacarpaea delavayi* | 7.4 |
| *Oreophyton falcatum* | 4.1 |
| *Kernera saxatilis* | 3.1 |
| *Maerua oblongifolia* | 6.5 |
| *Gynandropsis gynandra* | 4.2 |
| *Vasconcellea cundinamarcensis* | 14.9 |
| *Acer davidii* | 2.8 |
| *Acer pictum* | 26.9 |
| *Nephelium lappaceum* | 6.8 |
| *Citrus sunki* | 6.4 |
| *Zanthoxylum motuoense* | 3.0 |
| *Xylocarpus granatum* | 10.9 |
| *Hibiscus rosa-sinensis* | 12.8 |
| *Reevesia thyrsoidea* | 2.7 |
| *Pelargonium echinatum* | 4.5 |
| *Pelargonium dolomiticum* | 2.0 |
| *Linochilus apiculatus* | 2418.3 |
| *Leontopodium leiolepis* | 95.9 |
| *Helichrysum italicum* | 58.8 |
| *Anaphalis sinica* | 80.1 |
| *Gynura cusimbua* | 10.7 |
| *Crassocephalum crepidioides* | 136.4 |
| *Saussurea kingii* | 61.3 |
| *Siphocampylus krauseanus* | 15.9 |
| *Zabelia tyaihyoni* | 20.2 |
| *Leycesteria formosa* | 3.7 |
| *Bupleurum angustissimum* | 45.8 |
| *Bupleurum rockii* | 3.9 |
| *Tongoloa silaifolia* | 7.1 |
| *Panax vietnamensis* | 8.4 |
| *Helwingia himalaica* | 8.9 |
| *Orobanche austrohispanica* | 8.7 |
| *Mentha canadensis* | 3.9 |
| *Elsholtzia rugulosa* | 4.6 |
| *Clerodendrum bungei* | 316.3 |
| *Clerodendrum trichotomum* | 4.6 |
| *Adenocalymma hatschbachii* | 11.3 |
| *Adenocalymma allamandiflorum* | 4.6 |
| *Adenocalymma subspicatum* | 3.0 |
| *Barleria prionitis* | 11.3 |
| *Syringa wolfii* | 20.4 |
| *Chionanthus ramiflorus* | 1.0 |
| *Ligustrum japonicum* | 7.1 |
| *Notelaea venosa* | 1.0 |
| *Paraboea dictyoneura* | 4.6 |
| *Paraboea rufescens* | 0.7 |
| *Dorcoceras hygrometricum* | 6.7 |
| *Plantago depressa* | 8.4 |
| *Puchiumazus lanceifolius* | 3.1 |
| *Torenia concolor* | 0.8 |
| *Dodartia orientalis* | 2.7 |
| *Gentiana apiata* | 7.6 |
| *Metagentiana rhodantha* | 3.6 |
| *Fosbergia shweliensis* | 5.9 |
| *Uncaria rhynchophylla* | 4.9 |
| *Hoya liangii* | 5.3 |
| *Atropa belladonna* | 6.5 |
| *Physochlaina orientalis* | 3.4 |
| *Primula hubeiensis* | 8.0 |
| *Primula rubifolia* | 6.6 |
| *Primula oreodoxa* | 10.9 |
| *Primula vilmoriniana* | 21.2 |
| *Primula dumicola* | 3.0 |
| *Androsace mariae* | 5.6 |
| *Androsace laxa* | 8.5 |
| *Myrsine africana* | 7.0 |
| *Ardisia bullata* | 13.1 |
| *Elingamita johnsonii* | 8.1 |
| *Monotropa hypopitys* | 1.3 |
| *Styrax chinensis* | 25.1 |
| *Actinidia rubus* | 10.1 |
| *Saurauia tristyla* | 8.6 |
| *Mimusops coriacea* | 12.0 |
| *Diospyros celebica* | 14.8 |
| *Cornus peruviana* | 8.5 |
| *Deutzia glabrata* | 5.7 |
| *Philadelphus calvescens* | 2.8 |
| *Rodgersia aesculifolia* | 3.4 |
| *Colobanthus acicularis* | 5.7 |
| *Calligonum jeminaicum* | 4.9 |
| *Talinum paniculatum* | 5.1 |
| *Dendrobium nobile* | 3.2 |
| *Cymbidium mannii* | 1.4 |
| *Eulophia zollingeri* | 0.8 |
| *Cephalantheropsis obcordata* | 3.5 |
| *Allium chinense* | 1.0 |
| *Allium mairei* | 1.2 |
| *Allium trifurcatum* | 1.0 |
| *Allium plurifoliatum* | 1.4 |
| *Lycoris aurea* | 9.8 |
| *Chlorogalum pomeridianum* | 4.3 |
| *Chlorophytum comosum* | 4.0 |
| *Oziroe biflora* | 0.7 |
| *Hypolytrum nemorum* | 15.0 |
| *Roscoea alpina* | 1.1 |
| *Ensete superbum* | 1.6 |
| *Xerophyllum tenax* | 2.5 |
| *Tricyrtis formosana* | 2.5 |
| *Lloydia tibetica* | 1.1 |
| *Colchicum autumnale* | 1.6 |
| *Gloriosa superba* | 0.6 |
| *Pistia stratiotes* | 3.7 |
| *Anemone trullifolia* | 2.2 |
| *Dichocarpum fargesii* | 1.8 |
| *Leptopyrum fumarioides* | 0.4 |
| *Corydalis saxicola* | 5.6 |
| *Coreanomecon hylomeconoides* | 2.5 |
| *Diphylleia grayi* | 6.8 |
| *Beilschmiedia rufohirtella* | 2.6 |
| *Caryodaphnopsis tonkinensis* | 1.8 |
| *Macadamia integrifolia* | 4.3 |
| *Sarcandra glabra* | 2.6 |
| *Juglans cinerea* | 15.0 |
| *Eucalyptus cloeziana* | 1436.6 |
| *Eucalyptus saligna* | 4104.6 |
| *Eucalyptus marginata* | 4401.4 |
| *Eucalyptus cladocalyx* | 8642.5 |
| *Eucalyptus nitens* | 1123.8 |
| *Pemphis acidula* | 109.6 |
| *Taraxacum kok-saghyz* | 77.9 |
| *Stebbinsia umbrella* | 125.9 |
| *Lactuca tatarica* | 228.1 |
| *Sonchus arvensis* | 43.3 |
| *Bidens pilosa* | 45.2 |
| *Marshallia ramosa* | 135.7 |
| *Baccharis genistelloides* | 31.5 |
| *Solidago decurrens* | 31.1 |
| *Nannoglottis ravida* | 91.8 |
| *Parastrephia quadrangularis* | 52.2 |
| *Lagenophora cuchumatanica* | 42.5 |
| *Pericallis hybrida* | 43.6 |
| *Jacobaea vulgaris* | 273.7 |
| *Emilia sonchifolia* | 37.5 |
| *Senecio vulgaris* | 41.2 |
| *Sinosenecio jishouensis* | 274.9 |
| *Chrysanthemum lucidum* | 89.3 |
| *Phaeostigma variifolium* | 23.7 |
| *Synurus deltoides* | 55.1 |
| *Arctium lappa* | 109.4 |
| ***Arctotheca calendula*** | **25.5** |
| *Myripnois dioica* | 85.5 |
| *Daucus carota* | 12.5 |
| *Adenocalymma nodosum* | 2.7 |
| *Oresitrophe rupifraga* | 1.2 |
| *Prunus speciosa* | 10.0 |
| *Prunus apetala* | 17.1 |
| *Malus baccata* | 142.6 |
| *Malus trilobata* | 3.3 |
| *Cotoneaster microphyllus* | 3.7 |
| *Photinia blinii* | 81.1 |
| *Pyrus ussuriensis* | 2.0 |
| *Hesperomeles goudotiana* | 6.1 |
| *Rubus pentagonus* | 8.0 |
| *Rubus crassifolius* | 3.7 |
| *Rosa cymosa* | 1.9 |
| *Fragaria nilgerrensis* | 1.3 |
| *Fragaria viridis* | 3.2 |
| *Elaeagnus macrophylla* | 1.7 |
| *Lathyrus japonicus* | 1.5 |
| *Trifolium meduseum* | 9.8 |
| *Medicago ruthenica* | 0.7 |
| *Oxytropis arctobia* | 9.4 |
| *Caragana stenophylla* | 1.8 |
| *Halimodendron halodendron* | 4.2 |
| *Glycine sp. D3 (G. tomentella sensu lato)* | 1.5 |
| *Glycine cyrtoloba* | 9.5 |
| *Eriosema crinitum* | 3.6 |
| *Kummerowia striata* | 3.5 |
| *Dalbergia armata* | 10.3 |
| *Dalbergia hupeana* | 3.2 |
| *Pterocarpus santalinus* | 0.7 |
| *Thermopsis turkestanica* | 1.1 |
| *Lupinus albus* | 4.0 |
| *Piptadenia communis* | 13.8 |
| *Parkia javanica* | 1.4 |
| *Pithecellobium flexicaule* | 4.6 |
| *Gleditsia japonica* | 2.9 |
| *Cercis glabra* | 3.8 |
| *Tamarindus indica* | 3.5 |
| *Passiflora serratodigitata* | 2.4 |
| *Passiflora nitida* | 2.4 |
| *Passiflora biflora* | 3.0 |
| *Passiflora candollei* | 1.8 |
| *Mitostemma brevifilis* | 1.0 |
| *Populus wilsonii* | 0.2 |
| *Salix triandroides* | 3.6 |
| *Xylosma longifolia* | 3.3 |
| *Olmediella betschleriana* | 2.5 |
| *Licania minutiflora* | 3.4 |
| *Licania tomentosa* | 7.9 |
| *Parastemon urophyllus* | 3.8 |
| *Exellodendron barbatum* | 0.4 |
| *Garcinia subelliptica* | 9.4 |
| *Aspidopterys obcordata* | 9.2 |
| *Fagus engleriana* | 3.0 |
| *Fagus japonica* | 13.5 |
| *Quercus coccinea* | 6.9 |
| *Quercus pannosa* | 6.1 |
| *Carya cathayensis* | 14.6 |
| *Cyclocarya paliurus* | 1.0 |
| *Alnus japonica* | 1.2 |
| *Carpinus hebestroma* | 24.9 |
| *Trichosanthes lobata* | 4.1 |
| *Gynostemma laxiflorum* | 11.0 |
| *Corallocarpus boehmii* | 5.7 |
| *Eucalyptus diversicolor* | 4298.0 |
| *Eucalyptus verrucata* | 215.4 |
| *Eucalyptus dumosa* | 138.5 |
| *Eucalyptus grandis* | 475.6 |
| *Corymbia torelliana* | 379.0 |
| *Plinia aureana* | 1599.7 |
| *Melaleuca cajuputi* | 762.8 |
| *Melaleuca leucadendra* | 363.6 |
| *Oenothera villaricae* | 75.8 |
| *Epilobium hirsutum* | 30.6 |
| *Terminalia catappa* | 26.3 |
| *Lumnitzera racemosa* | 15.1 |
| *Solms-laubachia prolifera* | 66.7 |
| *Solms-laubachia minor* | 6.6 |
| *Solms-laubachia zhongdianensis* | 5.0 |
| *Solms-laubachia linearis* | 1.9 |
| *Solms-laubachia kashgarica* | 1.2 |
| *Lachnoloma lehmannii* | 3.5 |
| *Leptaleum filifolium* | 2.4 |
| *Fourraea alpina* | 2.5 |
| *Horwoodia dicksoniae* | 3.8 |
| *Litwinowia tenuissima* | 6.0 |
| *Menonvillea spathulata* | 11.7 |
| *Aethionema cordifolium* | 11.0 |
| *Cardamine hirsuta* | 1.0 |
| *Brassica nigra* | 0.2 |
| *Iskandera alaica* | 1.8 |
| *Lepidium latifolium* | 1.2 |
| *Clypeola jonthlaspi* | 5.9 |
| *Noccaea caerulescens* | 1.9 |
| *Clausia aprica* | 58.6 |
| *Dithyrea californica* | 3.2 |
| *Crateva unilocularis* | 2.7 |
| *Thulinella chrysantha* | 2.6 |
| *Akania lucens* | 8.0 |
| *Acer macrophyllum* | 4.8 |
| *Acer negundo* | 2.4 |
| *Aesculus wangii* | 17.8 |
| *Glycosmis mauritiana* | 9.7 |
| *Zanthoxylum nitidum* | 233.3 |
| *Aglaia odorata* | 6.5 |
| *Cedrela odorata* | 4.0 |
| *Mangifera persiciforma* | 20.7 |
| *Spondias bahiensis* | 6.9 |
| *Boswellia sacra* | 2.2 |
| *Gossypium thurberi* | 58.1 |
| *Gossypium sturtianum* | 17.9 |
| *Gossypium anomalum* | 19.2 |
| *Urena procumbens* | 5.2 |
| *Alcea rosea* | 0.9 |
| *Firmiana pulcherrima* | 0.5 |
| *Corchorus olitorius* | 9.6 |
| *Pachira macrocarpa* | 264.2 |
| *Edgeworthia gardneri* | 7.1 |
| *Vatica guangxiensis* | 4.1 |
| *Parashorea chinensis* | 2.8 |
| *Helianthemum songaricum* | 3.9 |
| *Pelargonium tetragonum* | 9.9 |
| *Pelargonium australe* | 6.2 |
| *Stachyurus chinensis* | 3.7 |
| *Ampelopsis humulifolia* | 26.7 |
| *Tetrastigma planicaule* | 3.8 |
| *Crepidiastrum denticulatum* | 168.7 |
| *Sonchus brachyotus* | 157.3 |
| *Bidens pachyloma* | 33.7 |
| *Ambrosia artemisiifolia* | 15.3 |
| *Marshallia trinervia* | 15.9 |
| *Baccharis aliena* | 34.2 |
| *Oritrophium peruvianum* | 49.0 |
| *Chrysanthemum indicum* | 1004.8 |
| *Saussurea japonica* | 61.5 |
| *Saussurea tridactyla* | 51.7 |
| *Atractylodes japonica* | 132.5 |
| *Carthamus tinctorius* | 21.7 |
| *Centaurea diffusa* | 37.8 |
| *Pertya multiflora* | 52.3 |
| *Ainsliaea latifolia* | 34.2 |
| *Nymphoides crenata* | 26.0 |
| *Adenophora erecta* | 8.1 |
| *Burmeistera fuscoapicata* | 2.0 |
| *Burmeistera parviflora* | 5.3 |
| *Burmeistera cyclostigmata* | 11.7 |
| *Burmeistera ceratocarpa* | 11.7 |
| *Codonopsis minima* | 8.9 |
| *Centropogon nigricans* | 6.2 |
| *Hanabusaya asiatica* | 1.3 |
| *Tetradoxa omeiensis* | 1.4 |
| *Lonicera ferdinandi* | 5.8 |
| *Lonicera calcarata* | 3.6 |
| *Lonicera vesicaria* | 0.7 |
| *Patrinia heterophylla* | 10.2 |
| *Patrinia scabra* | 18.1 |
| *Peucedanum japonicum* | 2.3 |
| *Angelica megaphylla* | 6.7 |
| *Bupleurum dracaenoides* | 7.2 |
| *Bupleurum yinchowense* | 10.2 |
| *Schefflera heptaphylla* | 3.7 |
| *Eleutherococcus trifoliatus* | 4.7 |
| *Pedicularis dissecta* | 2.1 |
| *Brandisia swinglei* | 2.8 |
| *Salvia petrophila* | 2.7 |
| *Dracocephalum moldavica* | 4.8 |
| *Paraphlomis koreana* | 7.6 |
| *Chelonopsis souliei* | 2.9 |
| *Adenocalymma marginatum* | 6.6 |
| *Jacaranda mimosifolia* | 2.9 |
| *Strobilanthes cusia* | 3.3 |
| *Chionanthus ligustrinus* | 0.4 |
| *Fraxinus hupehensis* | 4.6 |
| *Noronhia brevituba* | 1.1 |
| *Picconia azorica* | 9.7 |
| *Myxopyrum hainanense* | 4.1 |
| *Primulina eburnea* | 1.0 |
| *Paraboea filipes* | 4.1 |
| *Genlisea filiformis* | 5.2 |
| *Neopicrorhiza scrophulariiflora* | 0.8 |
| *Scrophularia cephalantha* | 1.2 |
| *Mazus omeiensis* | 9.6 |
| *Torenia fournieri* | 1.6 |
| *Gentiana trichotoma* | 8.3 |
| *Swertia hispidicalyx* | 6.5 |
| *Lomatogoniopsis alpina* | 6.9 |
| *Oldenlandia brachypoda* | 20.6 |
| *Leptodermis scabrida* | 2.0 |
| *Rauvolfia serpentina* | 10.7 |
| *Alstonia scholaris* | 9.5 |
| *Nicotiana sylvestris* | 6.9 |
| *Calibrachoa hybrid cultivar* | 2.5 |
| *Primula obconica* | 11.6 |
| *Primula waltonii* | 8.1 |
| *Primula tsiangii* | 3.2 |
| *Ardisia solanacea* | 48.6 |
| *Ardisia japonica* | 10.6 |
| *Myrsine sandwicensis* | 1.9 |
| *Rhododendron simsii* | 20.5 |
| *Camellia yunnanensis* | 9.4 |
| *Diospyros nigra* | 4.4 |
| *Jamesia americana* | 2.2 |
| *Caiophora cirsiifolia* | 5.7 |
| *Davidia involucrata* | 6.1 |
| *Chenopodium quinoa* | 120.9 |
| *Dysphania botrys* | 10.1 |
| *Salicornia bigelovii* | 110.0 |
| *Fagopyrum tataricum* | 8.7 |
| *Rheum pumilum* | 0.2 |
| *Rheum nobile* | 4.2 |
| *Rumex acetosa* | 87.4 |
| *Rumex nepalensis* | 27.2 |
| *Silene capitata* | 5.3 |
| *Acleisanthes obtusa* | 4.0 |
| *Graptopetalum amethystinum* | 2.0 |
| *Paeonia brownii* | 3.1 |
| *Paeonia obovata* | 1.9 |
| *Neottia fugongensis* | 2.1 |
| *Dendrobium crepidatum* | 0.1 |
| *Bulbophyllum steyermarkii* | 5.6 |
| *Bulbophyllum exaltatum* | 2.5 |
| *Bulbophyllum weddellii* | 0.8 |
| *Liparis nervosa* | 2.3 |
| *Erycina pusilla* | 3.3 |
| *Changnienia amoena* | 2.9 |
| *Calanthe lyroglossa* | 1.6 |
| *Vanda concolor* | 1.5 |
| *Gastrochilus calceolaris* | 1.1 |
| *Eurystyles cotyledon* | 3.6 |
| *Habenaria pantlingiana* | 1.6 |
| *Ponerorchis gracilis* | 0.8 |
| *Allium ampeloprasum* | 0.4 |
| *Polygonatum cyrtonema* | 0.9 |
| *Iris odaesanensis* | 1.3 |
| *Iris rossii* | 2.2 |
| *Aloe maculata* | 0.6 |
| *Setaria viridis* | 0.8 |
| *Zea mays* | 6.4 |
| *Merxmuellera tsaratananensis* | 0.3 |
| *Streptogyna americana* | 0.9 |
| *Joinvillea ascendens* | 0.7 |
| *Dioscorea preussii* | 2.5 |
| *Lilium philadelphicum* | 0.8 |
| *Lilium distichum* | 2.3 |
| *Wolffia brasiliensis* | 3.6 |
| *Spirodela polyrhiza* | 4.0 |
| *Pinellia peltata* | 6.3 |
| *Zamioculcas zamiifolia* | 2.6 |
| *Amorphophallus titanum* | 1.7 |
| *Colocasia esculenta* | 0.2 |
| *Oxygraphis glacialis* | 2.0 |
| *Actaea dahurica* | 1.0 |
| *Adonis amurensis* | 0.2 |
| *Semiaquilegia adoxoides* | 1.6 |
| *Ranzania japonica* | 3.0 |
| *Pericampylus glaucus* | 2.6 |
| *Stauntonia chinensis* | 1.4 |
| *Sinofranchetia chinensis* | 2.3 |
| *Litsea ichangensis* | 4.8 |
| *Calycanthus chinensis* | 2.3 |
| *Idiospermum australiense* | 1.7 |
| *Aristolochia tagala* | 1.1 |
| *Houttuynia cordata* | 2.4 |
| *Annona reticulata* | 2.6 |
| *Pseudowintera colorata* | 1.1 |
| *Uraria lagopodoides* | 2.8 |
| *Spondias tuberosa* | 0.9 |
| *Syringa pinnatifolia* | 4.5 |
| *Symplocos ovatilobata* | 7.4 |
| *Kissenia capensis* | 6.2 |
| *Salsola abrotanoides* | 10.0 |
| *Dioscorea elephantipes* | 0.4 |
| *Aponogeton rehmannii* | 1.2 |
| *Prunus verecunda* | 18.5 |
| *Crataegus hupehensis* | 1.1 |
| *Chaenomeles japonica* | 154.7 |
| *Rubus trifidus* | 0.9 |
| *Potentilla glabra* | 2.8 |
| *Ficus hirta* | 0.9 |
| *Artocarpus camansi* | 7.8 |
| *Ulmus chenmoui* | 1.2 |
| *Gleditsia sinensis* | 1.1 |
| *Passiflora quadrangularis* | 1.1 |
| *Populus pseudoglauca* | 43.1 |
| *Afrolicania elaeosperma* | 4.6 |
| *Acioa guianensis* | 1.3 |
| *Viola websteri* | 7.0 |
| *Byrsonima crassifolia* | 9.6 |
| *Castanea seguinii* | 8.9 |
| *Engelhardia roxburghiana* | 1.2 |
| *Eucalyptus umbra* | 3341.3 |
| *Eucalyptus woollsiana* | 2503.3 |
| *Eucalyptus aromaphloia* | 293.0 |
| *Eucalyptus melliodora* | 317.9 |
| *Eucalyptus spathulata* | 216.4 |
| *Eucalyptus globulus* | 275.5 |
| *Eucalyptus regnans* | 784.9 |
| *Eucalyptus cajuputea* | 133.4 |
| *Corymbia henryi* | 2670.2 |
| *Corymbia maculata* | 3306.7 |
| *Angophora costata* | 35818.6 |
| *Lagerstroemia fauriei* | 266.3 |
| *Trapa kozhevnikoviorum* | 50.2 |
| *Osbeckia stellata* | 14.6 |
| *Barthea barthei* | 11.6 |
| *Miconia dodecandra* | 17.1 |
| *Combretum indicum* | 14.4 |
| *Solms-laubachia jafrii* | 1.1 |
| *Schrenkiella parvula* | 2.6 |
| *Arabis verna* | 5.1 |
| *Malcolmia maritima* | 1.0 |
| *Crucihimalaya wallichii* | 7.1 |
| *Bunias orientalis* | 1.3 |
| *Erysimum odoratum* | 2.5 |
| *Cleome pallida* | 7.9 |
| *Koelreuteria paniculata* | 6.0 |
| *Spondias dulcis* | 4.5 |
| *Pistacia chinensis* | 1.0 |
| *Thespesia populnea* | 5.1 |
| *Theobroma cacao* | 7.1 |
| *Aquilaria sinensis* | 0.6 |
| *Balanocarpus heimii* | 7.3 |
| *Taraxacum hallaisanense* | 56.8 |
| *Lapsanastrum humile* | 328.5 |
| *Helianthus atrorubens* | 25.0 |
| *Xanthium sibiricum* | 64.6 |
| *Xanthium spinosum* | 16.3 |
| *Aldama discolor* | 4.1 |
| *Tithonia diversifolia* | 31.8 |
| *Marshallia grandiflora* | 10.8 |
| *Sigesbeckia orientalis* | 7.4 |
| *Linochilus eriophorus* | 3.6 |
| *Erigeron canadensis* | 37.5 |
| *Diplostephium hippophae* | 18.7 |
| *Laestadia muscicola* | 30.0 |
| *Aster ageratoides* | 16.1 |
| *Aster spathulifolius* | 6.6 |
| *Llerasia caucana* | 31.1 |
| *Chrysanthemum vestitum* | 7.4 |
| *Artemisia maritima* | 240.9 |
| *Artemisia selengensis* | 3.4 |
| *Neopallasia pectinata* | 122.1 |
| *Saussurea medusa* | 16.9 |
| *Adenophora stricta* | 3.1 |
| *Lonicera praeflorens* | 1.8 |
| *Angelica sinensis* | 2.0 |
| *Ligusticum delavayi* | 3.2 |
| *Ligusticum pteridophyllum* | 0.3 |
| *Panax quinquefolius* | 5.4 |
| *Phelipanche purpurea* | 2.8 |
| *Pedicularis cheilanthifolia* | 1.3 |
| *Pedicularis shansiensis* | 6.0 |
| *Lavandula angustifolia* | 2.8 |
| *Cymaria dichotoma* | 2.7 |
| *Pleonotoma albiflora* | 3.1 |
| *Chionanthus quadristamineus* | 7.4 |
| *Chionanthus rupicola* | 0.9 |
| *Echinacanthus longipes* | 3.5 |
| *Primulina huaijiensis* | 2.1 |
| *Paraboea wenshanensis* | 0.6 |
| *Lagotis yunnanensis* | 4.2 |
| *Ophiorrhiza densa* | 3.6 |
| *Foonchewia coriacea* | 7.5 |
| *Neolamarckia cadamba* | 4.4 |
| *Gymnema sylvestre* | 2.3 |
| *Ipomoea carnea* | 0.2 |
| *Tubocapsicum anomalum* | 1.0 |
| *Primula filchnerae* | 6.7 |
| *Androsace erecta* | 3.4 |
| *Lysimachia hemsleyana* | 12.3 |
| *Camellia granthamiana* | 19.0 |
| *Stewartia villosa* | 6.8 |
| *Diospyros rhombifolia* | 10.6 |
| *Diospyros virginiana* | 12.3 |
| *Cornus eydeana* | 7.4 |
| *Cornus disciflora* | 4.5 |
| *Rhodiola sexifolia* | 2.4 |
| *Scurrula parasitica* | 6.4 |
| *Phalaenopsis lowii* | 3.2 |
| *Paphiopedilum delenatii* | 3.6 |
| *Paphiopedilum philippinense* | 0.3 |
| *Hosta plantaginea* | 5.9 |
| *Aphyllanthes monspeliensis* | 3.2 |
| *Allium schoenoprasoides* | 2.2 |
| *Lolium perenne* | 2.8 |
| *Oryza coarctata* | 4.9 |
| *Eriachne compacta* | 0.1 |
| *Trillium camschatcense* | 1.9 |
| *Lilium regale* | 0.6 |
| *Sciaphila densiflora* | 0.3 |
| *Gymnaconitum gymnandrum* | 1.5 |
| *Eranthis stellata* | 3.9 |
| *Hylomecon japonica* | 1.0 |
| *Epimedium xichangense* | 8.1 |
| *Plagiorhegma dubium* | 1.8 |
| *Beilschmiedia appendiculata* | 4.4 |
| *Saururus chinensis* | 2.0 |
| *Piper laetispicum* | 1.1 |
| *Tasmannia lanceolata* | 2.7 |
| *Trithuria inconspicua* | 0.6 |
| *Populus davidiana* | 1.7 |
| *Capparis decidua* | 5.2 |
| *Edgeworthia chrysantha* | 4.9 |
| *Linochilus coriaceus* | 60.7 |
| *Diphelypaea coccinea* | 1.3 |
| *Paraboea sinensis* | 3.4 |
| *Gentiana urnula* | 1.5 |
| *Ophiorrhiza pumila* | 9.2 |
| *Primula veris* | 9.8 |
| *Carpenteria californica* | 2.8 |
| *Nassella hyalina* | 1.8 |
| *Achnatherum pekinense* | 1.4 |
| *Eria lasiopetala* | 0.4 |
| *Wolffiella lingulata* | 1.4 |
| *Torminalis clusii* | 0.0 |
| *Medicago falcata* | 6.4 |
| *Decorsea schlechteri* | 1.0 |
| *Passiflora lutea* | 1.6 |
| *Quercus tarokoensis* | 5.2 |
| *Swietenia mahagoni* | 7.8 |
| *Trachyspermum ammi* | 0.3 |
| *Pterygocalyx volubilis* | 2.3 |
| *Oldenlandia diffusa* | 51.0 |
| *Curculigo orchioides* | 0.7 |
| *Trillium govanianum* | 2.3 |
| *Syzygium jambos* | 115.2 |
| *Lagerstroemia subcostata* | 18.3 |
| *Matthiola incana* | 7.4 |
| *Matthiola ovatifolia* | 41.7 |
| *Calepina irregularis* | 2.0 |
| *Pelargonium cotyledonis* | 4.6 |
| *Diplostephium meyenii* | 272.5 |
| *Withania somnifera* | 16.4 |
| *Eleocharis dulcis* | 1.7 |
| *Triodia concinna* | 1.8 |
| *Eragrostis tenellula* | 0.4 |
| *Coelachne africana* | 1.0 |
| *Leptaspis banksii* | 0.1 |
| *Maianthemum bicolor* | 0.6 |
| *Plinia cauliflora* | 68.3 |
| *Linochilus mutiscuanus* | 2367.3 |
| *Aster hypoleucus* | 85.6 |
| *Silybum marianum* | 87.0 |
| *Seguieria aculeata* | 9.0 |
| *Ulmus americana* | 5.2 |
| *Ulmus pumila* | 39.2 |
| *Trifolium glanduliferum* | 6.3 |
| *Caragana rosea* | 3.5 |
| *Dunbaria nivea* | 2.0 |
| *Urariopsis brevissima* | 1.9 |
| *Sophora toromiro* | 14.6 |
| *Ormosia formosana* | 7.1 |
| *Senegalia catechu* | 2.8 |
| *Prosopis cineraria* | 46.8 |
| *Passiflora ligularis* | 2.4 |
| *Passiflora loefgrenii* | 4.1 |
| *Passiflora serrulata* | 4.7 |
| *Populus trichocarpa* | 1.8 |
| *Carrierea calycina* | 2.9 |
| *Homalium paniculiflorum* | 26.3 |
| *Quercus robur* | 7.8 |
| *Castanopsis echinocarpa* | 23.7 |
| *Carpinus caroliniana* | 10.2 |
| *Siraitia siamensis* | 4.7 |
| *Euonymus hamiltonianus* | 2.8 |
| *Eucalyptus albopurpurea* | 112.5 |
| *Eucalyptus camaldulensis* | 798.4 |
| *Eucalyptus aenea* | 674.6 |
| *Eucalyptus obliqua* | 130.3 |
| *Eucalyptus walshii* | 1266.3 |
| ***Eucalyptus baxteri*** | **149.3** |
| *Melastoma dodecandrum* | 15.1 |
| *Blastus pauciflorus* | 7.4 |
| *Lumnitzera littorea* | 15.8 |
| *Neotorularia korolkowii* | 1.7 |
| *Anzhengxia yechengnica* | 4.6 |
| *Raphanus sativus* | 2.5 |
| *Sinapis alba* | 3.2 |
| *Alyssum dasycarpum* | 4.2 |
| *Eutrema heterophyllum* | 6.6 |
| *Dimorphocarpa wislizeni* | 8.6 |
| *Exhalimolobos weddellii* | 4.2 |
| *Hesperis persica* | 3.3 |
| *Brayopsis alpaminae* | 2.7 |
| *Anelsonia eurycarpa* | 5.1 |
| *Conringia planisiliqua* | 1.0 |
| *Thlaspi arvense* | 2.3 |
| *Sisymbrium irio* | 1.4 |
| *Citrus cavaleriei* | 14.6 |
| *Citrus australasica* | 2.6 |
| *Acer henryi* | 3.8 |
| *Acer pseudosieboldianum* | 21.0 |
| *Acer truncatum* | 13.5 |
| *Cotinus coggygria* | 1.8 |
| *Peganum multisectum* | 8.5 |
| *Heritiera littoralis* | 6.9 |
| *Heritiera elata* | 9.3 |
| *Sterculia lanceolata* | 8.1 |
| *Sterculia monosperma* | 8.8 |
| *Ceiba speciosa* | 1.7 |
| *Daphne acutiloba* | 3.4 |
| *Stellera chamaejasme* | 2.2 |
| *Vatica rassak* | 3.6 |
| *Hopea chinensis* | 10.1 |
| *Hopea reticulata* | 12.2 |
| *Pelargonium citronellum* | 6.9 |
| *Ampelopsis japonica* | 3.4 |
| *Vitis rotundifolia* | 10.9 |
| *Crepidiastrum lanceolatum* | 127.7 |
| *Taraxacum brevicorniculatum* | 152.0 |
| *Taraxacum obtusifrons* | 110.8 |
| *Sonchus canariensis* | 420.6 |
| *Dendroseris berteroana* | 31.3 |
| *Bidens micrantha* | 69.1 |
| *Aldama linearis* | 37.6 |
| *Linochilus inesianus* | 4.1 |
| *Baccharis tricuneata* | 9.0 |
| *Heteroplexis incana* | 20.4 |
| *Conyza bonariensis* | 7.4 |
| *Diplostephium crypteriophyllum* | 11.3 |
| *Hinterhubera ericoides* | 27.5 |
| *Floscaldasia hypsophila* | 12.2 |
| *Laennecia sophiifolia* | 21.2 |
| *Aster pekinensis* | 21.7 |
| *Aster indicus* | 14.1 |
| *Artemisia frigida* | 17.6 |
| *Artemisia fukudo* | 5.0 |
| *Chrysanthemum boreale* | 5.8 |
| *Leucanthemum vulgare* | 59.4 |
| *Tanacetum coccineum* | 29.5 |
| *Sinosenecio oldhamianus* | 28.6 |
| *Cynara cornigera* | 61.3 |
| *Carduus tenuiflorus* | 8.8 |
| *Atractylodes carlinoides* | 95.6 |
| *Saussurea obvallata* | 9.1 |
| *Saussurea salwinensis* | 365.4 |
| *Saussurea pseudoleucoma* | 5.0 |
| *Saussurea leontodontoides* | 64.8 |
| *Burmeistera rubrosepala* | 39.2 |
| *Burmeistera loejtnantii* | 10.4 |
| *Lonicera maackii* | 53.4 |
| *Linnaea borealis* | 2.7 |
| *Angelica sylvestris* | 5.0 |
| *Anethum graveolens* | 0.8 |
| *Apium graveolens* | 3.5 |
| *Bupleurum yunnanense* | 1.0 |
| *Aralia continentalis* | 4.7 |
| *Pittosporum brevicalyx* | 11.5 |
| *Salvia digitaloides* | 0.6 |
| *Salvia merjamie* | 4.0 |
| *Clinopodium chinense* | 0.8 |
| *Plectranthus scutellarioides* | 1.4 |
| *Perilla frutescens* | 2.9 |
| *Pogostemon stellatus* | 0.6 |
| *Stachys byzantina* | 1.9 |
| *Scutellaria amoena* | 6.6 |
| *Pedicularis resupinata* | 3.8 |
| *Paraboea peltifolia* | 6.8 |
| *Genlisea aurea* | 11.9 |
| *Adenocalymma pedunculatum* | 3.6 |
| *Picconia excelsa* | 19.8 |
| *Osmanthus cooperi* | 79.2 |
| *Olea woodiana* | 7.0 |
| *Priogymnanthus hasslerianus* | 2.0 |
| *Verbascum phoeniceum* | 2.4 |
| *Silvianthus bracteatus* | 2.5 |
| *Swertia multicaulis* | 2.0 |
| *Swertia leducii* | 3.9 |
| *Swertia nervosa* | 2.7 |
| *Halenia elliptica* | 1.9 |
| *Asclepias nivea* | 5.2 |
| *Pergularia tomentosa* | 6.3 |
| *Solanum incanum* | 8.0 |
| *Arnebia tibetana* | 9.2 |
| *Primula woodwardii* | 7.1 |
| *Primula asarifolia* | 10.8 |
| *Actinidia setosa* | 5.2 |
| *Actinidia zhejiangensis* | 4.6 |
| *Caiophora lateritia* | 1.2 |
| *Dysphania pumilio* | 5.3 |
| *Rumex hypogaeus* | 26.5 |
| *Carnegiea gigantea* | 3.6 |
| *Semiliquidambar cathayensis* | 28.6 |
| *Tiarella polyphylla* | 15.7 |
| *Astilboides tabularis* | 3.0 |
| *Taxillus theifer* | 2.6 |
| *Dendrobium pseudotenellum* | 0.1 |
| *Calanthe delavayi* | 0.5 |
| *Habenaria chejuensis* | 1.8 |
| *Allium polyrhizum* | 7.0 |
| *Iris laevigata* | 3.2 |
| *Leptagrostis schimperiana* | 0.9 |
| *Gelidocalamus tessellatus* | 4.0 |
| *Catapodium rigidum* | 11.7 |
| *Cautleya gracilis* | 3.5 |
| *Pontederia crassipes* | 2.1 |
| *Dioscorea quartiniana* | 1.2 |
| *Dioscorea dumetorum* | 1.1 |
| *Scoliopus bigelovii* | 1.0 |
| *Disporum sessile* | 1.9 |
| *Arisaema ringens* | 15.9 |
| *Wolffia australiana* | 2.7 |
| *Caldesia grandis* | 2.0 |
| *Acorus tatarinowii* | 2.4 |
| *Isopyrum manshuricum* | 1.7 |
| *Corydalis conspersa* | 5.0 |
| *Nandina domestica* | 0.8 |
| *Arcangelisia gusanlung* | 2.3 |
| *Litsea glutinosa* | 1.0 |
| *Cryptocarya chinensis* | 0.9 |
| *Piper cenocladum* | 3.0 |
| *Drimys granadensis* | 1.6 |
| *Chloranthus japonicus* | 3.8 |
| *Dalbergia vietnamensis* | 18.3 |
| *Mallotus japonicus* | 2.2 |
| *Ostrya japonica* | 4.7 |
| *Casuarina glauca* | 11.5 |
| *Euonymus schensianus* | 0.4 |
| *Eucalyptus diversifolia* | 193.4 |
| *Solms-laubachia himalayensis* | 3.0 |
| *Solms-laubachia xerophyta* | 2.2 |
| *Commiphora wightii* | 3.0 |
| *Callianthe picta* | 1.4 |
| *Pelargonium incrassatum* | 2.7 |
| *Vitis aestivalis* | 2.3 |
| *Youngia japonica* | 35.4 |
| *Bidens asymmetrica* | 21.3 |
| *Helianthus debilis* | 38.7 |
| *Aldama goyazii* | 24.0 |
| *Aldama canescens* | 9.2 |
| *Iostephane heterophylla* | 15.1 |
| *Marshallia mohrii* | 2.8 |
| *Diplostephium hartwegii* | 44.0 |
| *Blakiella bartsiifolia* | 9.2 |
| *Linochilus antioquensis* | 3.1 |
| *Aster hersileoides* | 8.8 |
| *Atractylodes koreana* | 49.3 |
| *Saussurea salicifolia* | 23.9 |
| *Nymphoides hydrophylla* | 34.6 |
| *Aralia undulata* | 6.8 |
| *Noronhia clarinerva* | 4.3 |
| *Scopolia parviflora* | 0.7 |
| *Bougainvillea praecox* | 6.0 |
| *Loranthus pseudo-odoratus* | 7.3 |
| *Bulbophyllum regnellii* | 1.2 |
| *Habenaria flagellifera* | 0.7 |
| *Clivia miniata* | 0.4 |
| *Iris tectorum* | 1.0 |
| *Zeugites pittieri* | 1.5 |
| *Triodia stipoides* | 2.4 |
| *Styppeiochloa gynoglossa* | 2.7 |
| *Smilax microphylla* | 4.1 |
| *Sinopodophyllum hexandrum* | 0.9 |
| *Menispermum dauricum* | 2.7 |
| *Aristolochia manshuriensis* | 0.6 |
| *Helicia nilagirica* | 3.4 |
| *Tibetia himalaica* | 5.8 |
| *Trichosanthes kirilowii* | 4.6 |
| *Eucalyptus albens* | 316.4 |
| *Octoceras lehmannianum* | 2.1 |
| *Eutrema yungshunense* | 1.6 |
| *Arabidopsis pedemontana* | 1.7 |
| *Gossypium stocksii* | 8.3 |
| *Gossypium nelsonii* | 1.7 |
| *Galinsoga quadriradiata* | 52.2 |
| *Linochilus rhomboidalis* | 4.3 |
| *Chrysanthemum x morifolium* | 9.0 |
| *Ligularia virgaurea* | 25.8 |
| *Atractylodes lancea* | 45.3 |
| *Saussurea delavayi* | 16.6 |
| *Saussurea inversa* | 31.0 |
| *Nymphoides simulans* | 7.9 |
| *Dipelta elegans* | 1.2 |
| *Adenocalymma acutissimum* | 6.0 |
| *Capsicum lycianthoides* | 2.0 |
| *Primula poissonii* | 6.8 |
| *Oxychloris scariosa* | 3.6 |
| *Chloris truncata* | 1.0 |
| *Astrebla lappacea* | 4.4 |
| *Enteropogon ramosus* | 0.7 |
| *Melanocenchris abyssinica* | 3.6 |
| *Tripogonella loliiformis* | 1.5 |
| *Oropetium aristatum* | 0.6 |
| *Triodia tomentosa* | 1.8 |
| *Dactyloctenium radulans* | 1.5 |
| *Trichoneura grandiglumis* | 0.6 |
| *Vaseyochloa multinervosa* | 0.9 |
| *Eragrostis setifolia* | 4.2 |
| *Sporobolus maritimus* | 9.9 |
| *Lecomtella madagascariensis* | 0.9 |
| *Oplismenus hirtellus* | 4.6 |
| *Paraneurachne muelleri* | 1.4 |
| *Chasmanthium laxum* | 0.9 |
| *Eriachne tenuiculmis* | 1.5 |
| *Limnopoa meeboldii* | 1.7 |
| *Pratochloa walteri* | 0.4 |
| *Lamarckia aurea* | 0.7 |
| *Nardus stricta* | 0.3 |
| *Oryza eichingeri* | 5.0 |
| *Oberonia japonica* | 1.2 |
| *Viola ulleungdoensis* | 2.4 |
| *Eucalyptus odorata* | 255.6 |
| *Taraxacum mongolicum* | 10.4 |
| *Tetrataenium yunnanense* | 0.5 |
| *Anemopaegma oligoneuron* | 1.3 |
| *Ipomoea aquatica* | 1.7 |
| *Styrax ramirezii* | 1.0 |
| *Rhododendron datiandingense* | 11.4 |
| *Paeonia veitchii* | 2.1 |
| *Dendrobium xichouense* | 14.5 |
| *Astelia australiana* | 1.3 |
| *Aponogeton desertorum* | 0.7 |
| *Ensete livingstonianum* | 0.3 |
| *Stenospermation multiovulatum* | 1.0 |
| *Eucalyptus populnea* | 109.1 |
| *Eucalyptus smithii* | 55.2 |
| *Eucalyptus porosa* | 154.1 |
| *Eucalyptus delegatensis* | 44.6 |
| *Angophora floribunda* | 2234.5 |
| *Lagerstroemia indica* | 17.2 |
| *Lagerstroemia venusta* | 130.1 |
| *Lagerstroemia floribunda* | 1.4 |
| *Lagerstroemia intermedia* | 11.3 |
| *Lagerstroemia excelsa* | 46.2 |
| *Heterotis rotundifolia* | 4.1 |
| *Oenothera curtiflora* | 11.0 |
| *Oenothera biennis* | 22.8 |
| *Epilobium ulleungensis* | 9.4 |
| *Solms-laubachia baiogoinensis* | 1.6 |
| *Solms-laubachia lanata* | 1.2 |
| *Solms-laubachia platycarpa* | 0.8 |
| *Solms-laubachia angustifolia* | 1.2 |
| *Solms-laubachia tianbaoshanensis* | 1.0 |
| *Neotorularia rossica* | 0.7 |
| *Tetracme quadricornis* | 2.8 |
| *Cymatocarpus pilosissimus* | 2.4 |
| *Cymatocarpus grossheimii* | 4.5 |
| *Streptoloma desertorum* | 2.1 |
| *Arabis josiae* | 1.8 |
| *Cardamine quinquefolia* | 3.8 |
| *Alyssum desertorum* | 2.4 |
| *Lepidium apetalum* | 3.2 |
| *Lepidium meyenii* | 0.2 |
| *Lepidium virginicum* | 0.8 |
| *Arabidopsis lyrata* | 2.7 |
| *Cochlearia borzaeana* | 5.8 |
| *Descurainia erodiifolia* | 0.4 |
| *Noccaea vesicaria* | 0.9 |
| *Boechera davidsonii* | 1.7 |
| *Hesperis tristis* | 1.9 |
| *Alliaria petiolata* | 1.2 |
| *Pachycladon enysii* | 1.9 |
| *Pachycladon cheesemanii* | 0.4 |
| *Hilliella paradoxa* | 1.9 |
| *Zanthoxylum piperitum* | 3.0 |
| *Zanthoxylum stenophyllum* | 2.3 |
| *Zanthoxylum acanthopodium* | 1.6 |
| *Zanthoxylum stipitatum* | 0.4 |
| *Acer pauciflorum* | 10.2 |
| *Acer stachyophyllum* | 2.2 |
| *Acer grandidentatum* | 3.9 |
| *Acer sutchuenense* | 1.4 |
| *Acer argutum* | 1.4 |
| *Litchi chinensis* | 1.3 |
| *Spondias mombin* | 1.4 |
| *Pistacia weinmaniifolia* | 14.6 |
| *Khaya madagascariensis* | 6.3 |
| *Xylocarpus moluccensis* | 10.4 |
| *Gossypium incanum* | 6.0 |
| *Hibiscus cannabinus* | 0.8 |
| *Abutilon theophrasti* | 1.0 |
| *Excentrodendron hsienmu* | 2.9 |
| *Edgeworthia albiflora* | 2.2 |
| *Pelargonium cucullatum* | 5.0 |
| *Pelargonium quercifolium* | 1.5 |
| *Pelargonium exhibens* | 0.3 |
| *Turpinia montana* | 2.2 |
| *Stachyurus yunnanensis* | 11.6 |
| *Malus prunifolia* | 42.5 |
| *Rubus incanus* | 113.0 |
| *Ficus pumila* | 3.3 |
| *Ulmus laciniata* | 25.9 |
| *Medicago archiducis-nicolai* | 10.6 |
| *Trifolium strictum* | 2.7 |
| *Astragalus gummifer* | 12.6 |
| *Astragalus laxmannii* | 0.2 |
| *Sophora macrocarpa* | 3.4 |
| *Dalbergia oliveri* | 4.8 |
| *Dalbergia obovata* | 3.3 |
| *Dalbergia martinii* | 2.4 |
| *Passiflora costaricensis* | 1.5 |
| *Passiflora laurifolia* | 1.7 |
| *Passiflora edmundoi* | 1.8 |
| *Populus mexicana* | 2.0 |
| *Bennettiodendron brevipes* | 3.9 |
| *Parinari campestris* | 4.9 |
| *Atuna racemosa* | 1.0 |
| *Quercus tungmaiensis* | 3.0 |
| *Quercus fleuryi* | 6.6 |
| *Carpinus fargesiana* | 40.2 |
| *Ostryopsis davidiana* | 5.5 |
| *Ostrya trichocarpa* | 2.8 |
| *Morella rubra* | 4.1 |
| *Gynostemma caulopterum* | 7.8 |
| *Gynostemma compressum* | 7.3 |
| *Citrullus ecirrhosus* | 7.3 |
| *Elaeocarpus braceanus* | 4.6 |
| *Vitis bryoniifolia* | 11.7 |
| *Taraxacum officinale* | 81.6 |
| *Bidens sandvicensis* | 69.8 |
| *Diplostephium spinulosum* | 7.7 |
| *Leucanthemum virgatum* | 14.9 |
| *Saussurea talungensis* | 15.9 |
| *Burmeistera auriculata* | 11.8 |
| *Lonicera fragrantissima* | 0.6 |
| *Diabelia sanguinea* | 1.4 |
| *Angelica tsinlingensis* | 1.6 |
| *Melanosciadium pimpinelloideum* | 1.0 |
| *Semenovia thomsonii* | 3.5 |
| *Bupleurum boissieuanum* | 2.5 |
| *Bupleurum shanianum* | 1.1 |
| *Bupleurum sikangense* | 0.2 |
| *Metapanax delavayi* | 3.2 |
| *Pittosporum tobira* | 3.4 |
| *Ilex intermedia* | 52.5 |
| *Ilex rotunda* | 3.3 |
| *Ilex paraguariensis* | 2.9 |
| *Salvia nanchuanensis* | 2.6 |
| *Nepeta stewartiana* | 0.7 |
| *Caryopteris forrestii* | 3.5 |
| *Leonurus cardiaca* | 2.0 |
| *Scutellaria baicalensis* | 2.1 |
| *Vitex negundo* | 5.7 |
| *Genlisea tuberosa* | 2.9 |
| *Adenocalymma divaricatum* | 1.1 |
| *Amphilophium chocoense* | 5.7 |
| *Paraboea swinhoei* | 1.0 |
| *Paraboea martinii* | 0.9 |
| *Achimenes cettoana* | 1.1 |
| *Chionanthus pubescens* | 1.2 |
| *Chionanthus mala-elengi* | 3.1 |
| *Fraxinus pennsylvanica* | 3.9 |
| *Noronhia peglerae* | 9.1 |
| *Buddleja sessilifolia* | 3.5 |
| *Lancea hirsuta* | 1.5 |
| *Gynochthodes cochinchinensis* | 9.1 |
| *Gentiana delavayi* | 2.5 |
| *Gentiana triflora* | 10.5 |
| *Halenia corniculata* | 2.6 |
| *Vincetoxicum hainanense* | 1.1 |
| *Ipomoea asarifolia* | 2.0 |
| *Solanum dulcamara* | 2.1 |
| *Solanum glabratum* | 1.8 |
| *Solanum rostratum* | 1.3 |
| *Primula effusa* | 4.8 |
| *Halesia diptera* | 1.4 |
| *Impatiens mengtszeana* | 10.6 |
| *Actinidia kolomikta* | 2.3 |
| *Stewartia micrantha* | 1.5 |
| *Vitellaria paradoxa* | 2.4 |
| *Euryodendron excelsum* | 3.1 |
| *Cornus sessilis* | 2.3 |
| *Cornus macrophylla* | 17.0 |
| *Cornus chinensis* | 5.1 |
| *Muehlenbeckia axillaris* | 2.0 |
| *Muehlenbeckia gracillima* | 2.1 |
| *Beta patula* | 11.9 |
| *Dianthus caryophyllus* | 1.8 |
| *Colobanthus lycopodioides* | 4.3 |
| *Mirabilis himalaica* | 1.5 |
| *Mitella formosana* | 4.4 |
| *Loranthus odoratus* | 10.3 |
| *Loranthus europaeus* | 3.2 |
| *Loranthus tanakae* | 2.5 |
| *Viscum coloratum* | 5.1 |
| *Viscum ovalifolium* | 3.7 |
| *Dendrobium wardianum* | 2.8 |
| *Dendrobium spatella* | 0.4 |
| *Dendrobium parishii* | 2.5 |
| *Dendrobium brymerianum* | 0.9 |
| *Dendrobium chrysocrepis* | 1.4 |
| *Bulbophyllum inconspicuum* | 0.6 |
| *Bulbophyllum granulosum* | 1.7 |
| *Liparis makinoana* | 0.9 |
| *Epipactis mairei* | 0.8 |
| *Epipactis microphylla* | 1.4 |
| *Cymbidium macrorhizon* | 1.6 |
| *Cymbidium ensifolium* | 1.2 |
| *Oncidium hybrid cultivar* | 1.1 |
| *Calanthe rubens* | 2.0 |
| *Goodyera velutina* | 0.7 |
| *Habenaria radiata* | 0.8 |
| *Paphiopedilum dianthum* | 0.5 |
| *Phragmipedium longifolium* | 2.4 |
| *Cypripedium formosanum* | 1.5 |
| *Beschorneria septentrionalis* | 4.2 |
| *Hesperoyucca whipplei* | 3.0 |
| *Allium fetisowii* | 1.3 |
| *Iris sanguinea* | 0.7 |
| *Eremurus robustus* | 2.0 |
| *Hyacinthoides non-scripta* | 1.0 |
| *Bambusa oldhamii* | 1.7 |
| *Hitchcockella baronii* | 0.2 |
| *Froesiochloa boutelouoides* | 0.8 |
| *Thinopyrum elongatum* | 0.9 |
| *Ehrharta erecta* | 0.7 |
| *Ehrharta bulbosa* | 0.2 |
| *Axonopus ramosus* | 1.0 |
| *Danthoniopsis dinteri* | 0.7 |
| *Eustachys glauca* | 0.4 |
| *Uniola paniculata* | 0.9 |
| *Pharus lappulaceus* | 0.2 |
| *Leptaspis zeylanica* | 1.0 |
| *Sparganium eurycarpum* | 1.6 |
| *Typha orientalis* | 1.7 |
| *Alpinia katsumadae* | 0.3 |
| *Musa velutina* | 0.8 |
| *Pontederia cordata* | 0.4 |
| *Elaeis guineensis* | 1.7 |
| *Areca catechu* | 1.5 |
| *Veratrum japonicum* | 5.4 |
| *Paris thibetica* | 1.4 |
| *Paris luquanensis* | 4.7 |
| *Ypsilandra thibetica* | 0.4 |
| *Lilium souliei* | 1.4 |
| *Fritillaria persica* | 0.6 |
| *Fritillaria karelinii* | 0.5 |
| *Clintonia udensis* | 0.9 |
| *Dioscorea alata* | 0.2 |
| *Amorphophallus konjac* | 0.9 |
| *Dieffenbachia seguine* | 0.6 |
| *Aponogeton abyssinicus* | 3.1 |
| *Acorus americanus* | 1.3 |
| *Anemone reflexa* | 4.7 |
| *Hepatica maxima* | 2.4 |
| *Actaea asiatica* | 0.7 |
| *Thalictrum minus* | 7.2 |
| *Paraquilegia anemonoides* | 4.3 |
| *Caulophyllum robustum* | 0.9 |
| *Stephania dielsiana* | 1.0 |
| *Beilschmiedia brunnea* | 1.1 |
| *Lindera rubronervia* | 1.8 |
| *Lindera angustifolia* | 0.2 |
| *Nothaphoebe cavaleriei* | 11.7 |
| *Syndiclis fooningensis* | 1.9 |
| *Chimonanthus nitens* | 1.2 |
| *Magnolia cathcartii* | 1.0 |
| *Eucalyptus castrensis* | 20.0 |
| *Eucalyptus microcarpa* | 40.1 |
| *Eucalyptus sieberi* | 24.0 |
| *Melastoma candidum* | 2.8 |
| *Spryginia gracilis* | 10.8 |
| *Rhammatophyllum pachyrhizum* | 0.3 |
| *Rhammatophyllum erysimoides* | 0.3 |
| *Shangrilaia nana* | 0.6 |
| *Draba oligosperma* | 1.6 |
| *Aubrieta parviflora* | 0.1 |
| *Brassica rapa* | 17.3 |
| *Camelina sativa* | 0.9 |
| *Arabidella trisecta* | 0.2 |
| *Eudema nubigena* | 0.7 |
| *Merrillia caloxylon* | 0.1 |
| *Acer oblongum* | 5.0 |
| *Aesculus assamica* | 2.9 |
| *Sapindus mukorossi* | 1.8 |
| *Malva wigandii* | 1.2 |
| *Tilia mongolica* | 1.5 |
| *Vatica mangachapoi* | 7.1 |
| *Cytinus hypocistis* | 0.5 |
| *Pelargonium fulgidum* | 1.8 |
| *Artocarpus altilis* | 3.4 |
| *Ulmus elongata* | 1.3 |
| *Zelkova schneideriana* | 14.6 |
| *Oxytropis glabra* | 1.1 |
| *Vigna mungo* | 1.6 |
| *Dolichos falciformis* | 0.8 |
| *Grona heterocarpos* | 2.2 |
| *Passiflora oerstedii* | 0.6 |
| *Passiflora miniata* | 1.8 |
| *Passiflora capsularis* | 0.5 |
| *Alnus cordata* | 3.0 |
| *Cucurbita maxima* | 5.9 |
| *Marshallia graminifolia* | 7.4 |
| *Westoniella kohkemperi* | 5.0 |
| *Linochilus alveolatus* | 142.1 |
| *Linochilus violaceus* | 27.2 |
| *Linochilus rhododendroides* | 11.1 |
| *Aster tataricus* | 16.4 |
| *Ajania pacifica* | 6.6 |
| *Dipelta floribunda* | 0.5 |
| *Sinadoxa corydalifolia* | 1.0 |
| *Angelica cartilaginomarginata* | 1.7 |
| *Ligusticum capillaceum* | 4.9 |
| *Panax stipuleanatus* | 0.5 |
| *Dendropanax morbifer* | 5.5 |
| *Salvia chanryoenica* | 0.5 |
| *Salvia tiliifolia* | 1.5 |
| *Craniotome furcata* | 0.6 |
| *Stachys coccinea* | 2.7 |
| *Stenogyne haliakalae* | 1.1 |
| *Colquhounia vestita* | 2.7 |
| *Chionanthus pedunculatus* | 1.5 |
| *Chionanthus thorelii* | 2.4 |
| *Chionanthus virginicus* | 0.2 |
| *Ligustrum gracile* | 1.6 |
| *Fraxinus quadrangulata* | 0.8 |
| *Olea paniculata* | 1.4 |
| *Buddleja colvilei* | 3.2 |
| *Gentiana crassicaulis* | 7.0 |
| *Cynanchum wilfordii* | 1.3 |
| *Trompettia cardenasiana* | 0.6 |
| *Primula violaris* | 2.8 |
| *Primula chrysochlora* | 8.3 |
| *Lysimachia congestiflora* | 1.0 |
| *Halesia carolina* | 2.1 |
| *Changiostyrax dolichocarpus* | 1.8 |
| *Lucuma campechiana* | 3.6 |
| *Diospyros cathayensis* | 1.2 |
| *Calligonum caput-medusae* | 1.2 |
| *Dianthus chinensis* | 4.4 |
| *Colobanthus apetalus* | 0.9 |
| *Colobanthus subulatus* | 2.1 |
| *Phedimus kamtschaticus* | 0.9 |
| *Parrotia subaequalis* | 0.6 |
| *Loranthus lambertianus* | 0.4 |
| *Loranthus guizhouensis* | 1.0 |
| *Scurrula chingii* | 1.5 |
| *Viscum album* | 2.9 |
| *Dendrobium loddigesii* | 0.3 |
| *Dendrobium lohohense* | 1.0 |
| *Bulbophyllum mentosum* | 0.3 |
| *Anathallis obovata* | 1.3 |
| *Cymbidium tracyanum* | 0.8 |
| *Tainia dunnii* | 0.4 |
| *Gastrochilus japonicus* | 0.5 |
| *Thunia alba* | 0.3 |
| *Goodyera procera* | 0.8 |
| *Aspidogyne longicornu* | 0.2 |
| *Sauroglossum elatum* | 0.3 |
| *Cypripedium subtropicum* | 0.8 |
| *Allium macranthum* | 1.0 |
| *Allium fasciculatum* | 0.8 |
| *Allium monanthum* | 0.3 |
| *Allium pskemense* | 0.2 |
| *Yucca schidigera* | 5.6 |
| *Dracaena cambodiana* | 0.5 |
| *Iris ensata* | 1.3 |
| *Iris pseudacorus* | 0.2 |
| *Chusquea culeou* | 0.2 |
| *Alopecurus arundinaceus* | 0.2 |
| *Bromus catharticus* | 2.0 |
| *Panicum lycopodioides* | 0.1 |
| *Streptochaeta spicata* | 1.0 |
| *Cyperus aromaticus* | 0.9 |
| *Eleocharis cellulosa* | 0.9 |
| *Ensete ventricosum* | 1.2 |
| *Ensete glaucum* | 0.2 |
| *Lilium washingtonianum* | 0.6 |
| *Amana kuocangshanica* | 0.8 |
| *Dioscorea sagittifolia* | 2.8 |
| *Dioscorea collettii* | 0.7 |
| *Arisaema erubescens* | 0.7 |
| *Clematis loureiroana* | 0.5 |
| *Clematis taeguensis* | 0.3 |
| *Actaea vaginata* | 0.4 |
| *Aconitum brachypodum* | 4.9 |
| *Aquilegia barnebyi* | 1.8 |
| *Leontice incerta* | 1.5 |
| *Syndiclis anlungensis* | 1.1 |
| *Actinodaphne lancifolia* | 1.9 |
| *Calycanthus floridus* | 0.2 |
| *Aristolochia debilis* | 0.7 |
| *Aristolochia tubiflora* | 0.1 |
| *Piper kadsura* | 0.6 |
| *Magnolia odora* | 1.9 |
| *Magnolia macrophylla* | 0.4 |
| *Barclaya longifolia* | 0.5 |
| *Eucalyptus elata* | 26.0 |
| *Pleroma urvilleanum* | 3.5 |
| *Biscutella laevigata* | 0.4 |
| *Megacarpaea polyandra* | 0.4 |
| *Zanthoxylum piasezkii* | 1.7 |
| *Acer yangbiense* | 1.1 |
| *Acer pentaphyllum* | 2.0 |
| *Gossypium populifolium* | 0.9 |
| *Wikstroemia alternifolia* | 0.8 |
| *Rubus phoenicolasius* | 0.4 |
| *Fragaria daltoniana* | 1.0 |
| *Morus indica* | 2.6 |
| *Christia vespertilionis* | 0.8 |
| *Cyamopsis tetragonoloba* | 7.3 |
| *Dalbergia bariensis* | 0.8 |
| *Passiflora misera* | 1.7 |
| *Couepia subcordata* | 0.6 |
| *Castanea mollissima* | 3.0 |
| *Gynostemma burmanicum (nom. inval.)* | 1.2 |
| *Elaeocarpus decipiens* | 2.3 |
| *Vitis pseudoreticulata* | 3.2 |
| *Vitis yeshanensis* | 5.0 |
| *Aldama tukumanensis* | 7.8 |
| *Linochilus romeroi* | 10.0 |
| *Leucomeris decora* | 11.7 |
| *Burmeistera domingensis* | 2.1 |
| *Angelica keiskei* | 0.5 |
| *Petroselinum crispum* | 1.2 |
| *Bupleurum latissimum* | 0.5 |
| *Hansenia oviformis* | 1.9 |
| *Panax notoginseng* | 1.4 |
| *Ilex purpurea* | 1.3 |
| *Callicarpa bodinieri* | 1.4 |
| *Salvia meiliensis* | 2.3 |
| *Catalpa ovata* | 3.0 |
| *Chionanthus brassii* | 0.7 |
| *Fraxinus lanuginosa* | 0.4 |
| *Coffea arabica* | 1.6 |
| *Solanum richardii* | 0.4 |
| *Solanum macrocarpon* | 4.5 |
| *Withania coagulans* | 1.3 |
| *Physalis angulata* | 0.6 |
| *Arnebia guttata* | 5.0 |
| *Tapeinosperma netor* | 5.3 |
| *Camellia micrantha* | 37.6 |
| *Hydrangea obtusifolia* | 1.4 |
| *Dianthus longicalyx* | 1.1 |
| *Kalanchoe fedtschenkoi* | 1.0 |
| *Thrixspermum japonicum* | 0.2 |
| *Agave attenuata* | 2.2 |
| *Speirantha gardenii* | 0.5 |
| *Asparagus schoberioides* | 0.2 |
| *Iris setosa* | 0.3 |
| *Indocalamus wilsonii* | 0.1 |
| *Melica scabrosa* | 0.2 |
| *Zizania latifolia* | 0.2 |
| *Sacciolepis indica* | 0.2 |
| *Zingiber zerumbet* | 0.2 |
| *Kaempferia galanga* | 2.0 |
| *Typhonium blumei* | 0.2 |
| *Carlephyton glaucophyllum* | 0.1 |
| *Anemone raddeana* | 0.8 |
| *Coptis quinquesecta* | 0.5 |
| *Meconopsis punicea* | 0.7 |
| *Gymnospermium kiangnanense* | 0.2 |
| *Eucalyptus polybractea* | 77.5 |
| *Lagerstroemia siamica* | 1.8 |
| *Lagerstroemia limii* | 1.9 |
| *Oenothera glazioviana* | 13.6 |
| *Cardamine pentaphyllos* | 2.1 |
| *Morettia canescens* | 0.3 |
| *Koelreuteria bipinnata* | 2.1 |
| *Toona ciliata* | 0.6 |
| *Gossypium armourianum* | 4.3 |
| *Reevesia pycnantha* | 0.8 |
| *Ficus curtipes* | 1.5 |
| *Glycyrrhiza glabra* | 5.1 |
| *Glycine syndetika* | 1.4 |
| *Couepia grandiflora* | 1.6 |
| *Linochilus glutinosus* | 5.4 |
| *Arracacia xanthorrhiza* | 0.9 |
| *Schefflera actinophylla* | 4.7 |
| *Ilex vomitoria* | 1.2 |
| *Salvia nilotica* | 0.6 |
| *Agastache rugosa* | 1.1 |
| *Utricularia macrorhiza* | 9.2 |
| *Amphilophium cuneifolium* | 0.7 |
| *Amphilophium lactiflorum* | 1.6 |
| *Anemopaegma foetidum* | 0.4 |
| *Chionanthus polygamus* | 0.9 |
| *Ipomoea x leucantha* | 1.1 |
| *Solanum usambarense* | 1.1 |
| *Capsicum chacoense* | 1.0 |
| *Iochroma cyaneum* | 5.5 |
| *Fagopyrum luojishanense* | 2.4 |
| *Corylopsis coreana* | 3.8 |
| *Cercidiphyllum magnificum* | 3.8 |
| *Bulbophyllum epiphytum* | 0.5 |
| *Cymbidium goeringii* | 0.8 |
| *Cymbidium dayanum* | 0.2 |
| *Paphiopedilum violascens* | 0.3 |
| *Vanilla planifolia* | 0.4 |
| *Anthericum ramosum* | 0.2 |
| *Nolina atopocarpa* | 1.2 |
| *Asparagus setaceus* | 0.3 |
| *Allium changduense* | 0.3 |
| *Iris missouriensis* | 0.4 |
| *Aloe vera* | 0.5 |
| *Imperata cylindrica* | 0.7 |
| *Musa tonkinensis* | 1.1 |
| *Lilium henryi* | 1.7 |
| *Smilax glabra* | 0.3 |
| *Burmannia oblonga* | 0.2 |
| *Monstera adansonii* | 0.5 |
| *Stephania kwangsiensis* | 0.5 |
| *Beilschmiedia pierreana* | 0.3 |
| *Lindera reflexa* | 1.4 |
| *Magnolia officinalis* | 0.6 |
| *Eucalyptus wimmerensis* | 103.7 |
| *Eucalyptus silvestris* | 21.1 |
| *Pleroma semidecandrum* | 1.4 |
| *Leiospora eriocalyx* | 0.9 |
| *Strigosella scorpioides* | 0.6 |
| *Rhammatophyllum gaudanense* | 0.2 |
| *Boechera pulchra* | 0.5 |
| *Goldbachia laevigata* | 0.2 |
| *Conringia orientalis* | 0.1 |
| *Acer granatense* | 1.4 |
| *Gossypium klotzschianum* | 1.8 |
| *Rubus lambertianus* | 1.8 |
| *Ficus virens* | 0.5 |
| *Ficus benjamina* | 0.7 |
| *Ormosia xylocarpa* | 0.4 |
| *Dalbergia tonkinensis* | 2.7 |
| *Passiflora retipetala* | 0.5 |
| *Populus euphratica* | 0.6 |
| *Flacourtia rukam* | 0.4 |
| *Licania macrophylla* | 0.5 |
| *Hirtella suffulta* | 0.8 |
| *Viola raddeana* | 0.5 |
| *Quercus bawanglingensis* | 2.0 |
| *Cucumis x hytivus* | 0.8 |
| *Vitis rufotomentosa* | 8.7 |
| *Helianthus microcephalus* | 154.1 |
| *Marshallia caespitosa* | 14.3 |
| *Diplostephium glandulosum* | 8.6 |
| *Linochilus colombianus* | 3.9 |
| *Aster sampsonii* | 11.2 |
| *Artemisia capillaris* | 26.0 |
| *Ligularia hodgsonii* | 48.1 |
| *Cynara baetica* | 23.9 |
| *Burmeistera cylindrocarpa* | 0.9 |
| *Burmeistera resupinata* | 0.5 |
| *Hansenia weberbaueriana* | 1.0 |
| *Callicarpa arborea* | 0.2 |
| *Salvia przewalskii* | 0.3 |
| *Salvia miltiorrhiza* | 0.4 |
| *Amphilophium gnaphalanthum* | 0.2 |
| *Chionanthus parkinsonii* | 1.1 |
| *Ligustrum vulgare* | 1.4 |
| *Ligustrum ovalifolium* | 0.6 |
| *Mazus pumilus* | 0.5 |
| *Gynochthodes officinalis* | 28.0 |
| *Cynanchum auriculatum* | 1.0 |
| *Ipomoea maurandioides* | 0.2 |
| *Solanum campylacanthum* | 0.6 |
| *Nicotiana undulata* | 0.4 |
| *Petunia x hybrida* | 2.8 |
| *Primula ambita* | 0.5 |
| *Styrax obassis* | 0.5 |
| *Perkinsiodendron macgregorii* | 1.7 |
| *Rhododendron delavayi* | 1.2 |
| *Camellia kissii* | 2.9 |
| *Hydrangea luteovenosa* | 0.4 |
| *Hydrangea serrata* | 0.3 |
| *Paeonia lactiflora* | 0.5 |
| *Apostasia wallichii* | 10.0 |
| *Vanilla pompona* | 0.9 |
| *Allium przewalskianum* | 0.8 |
| *Hesperocallis undulata* | 0.3 |
| *Streptopus obtusatus* | 0.2 |
| *Asparagus officinalis* | 0.2 |
| *Aloidendron pillansii* | 0.2 |
| *Triticum monococcum* | 0.2 |
| *Oryza australiensis* | 0.1 |
| *Humbertochloa bambusiuscula* | 0.2 |
| *Stenotaphrum secundatum* | 0.2 |
| *Stenotaphrum subulatum* | 0.1 |
| *Panicum incomtum* | 0.7 |
| *Enneapogon caerulescens* | 0.3 |
| *Triraphis mollis* | 0.1 |
| *Sparganium stoloniferum* | 1.4 |
| *Lilium candidum* | 0.2 |
| *Fritillaria eduardii* | 0.2 |
| *Aletris fauriei* | 0.2 |
| *Xanthosoma helleborifolium* | 0.2 |
| *Actaea heracleifolia* | 0.2 |
| *Adonis sutchuenensis* | 0.8 |
| *Thalictrum viscosum* | 0.4 |
| *Coptis omeiensis* | 0.3 |
| *Dysosma delavayi* | 0.2 |
| *Alseodaphnopsis andersonii* | 1.1 |
| *Magnolia dixonii* | 1.2 |
| *Magnolia fraseri* | 0.1 |
| *Schisandra chinensis* | 0.3 |
| *Salvia yangii* | 0.8 |
| *Glandularia tenera* | 0.2 |
| *Cynosurus cristatus* | 1.9 |
| *Puccinellia distans* | 0.3 |
| *Isatis tinctoria* | 0.1 |
| *Bidens wiebkei* | 16.0 |
| *Haloxylon persicum* | 5.5 |
| *Colquhounia coccinea* | 4.7 |
| *Beta vulgaris* | 3.7 |
| *Salix arbutifolia* | 1.0 |
| *Citrullus rehmii* | 3.9 |
| *Acer sterculiaceum* | 1.2 |
| *Aldama gardneri* | 25.4 |
| *Ligularia mongolica* | 18.2 |
| *Abelia chinensis* | 3.1 |
| *Cheirodendron bastardianum* | 1.0 |
| *Pittosporum kerrii* | 5.1 |
| *Sonchus acaulis* | 48.6 |
| *Dendroseris marginata* | 4.1 |
| *Aldama bakeriana* | 11.0 |
| *Aldama grandiflora* | 3.2 |
| *Aldama arenaria* | 4.4 |
| *Aldama tuberosa* | 12.1 |
| *Aldama squalida* | 35.2 |
| *Aldama excelsa* | 1.9 |
| *Helianthus verticillatus* | 23.3 |
| *Helianthus argophyllus* | 1.5 |
| *Helianthus silphioides* | 6.0 |
| *Helianthus annuus* | 2.1 |
| *Eclipta alba* | 2.5 |
| *Sphagneticola calendulacea* | 0.9 |
| *Bidens campylotheca* | 4.3 |
| *Bidens forbesii* | 34.1 |
| *Bidens macrocarpa* | 5.5 |
| *Bidens amplectens* | 1.3 |
| *Bidens cervicata* | 4.2 |
| *Bidens menziesii* | 1.2 |
| *Galinsoga parviflora* | 21.5 |
| *Marshallia legrandii* | 21.9 |
| *Diplostephium espinosae* | 2.6 |
| *Diplostephium jelskii* | 5.2 |
| *Diplostephium sagasteguii* | 2.2 |
| *Diplostephium cinereum* | 4.2 |
| *Diplostephium juniperinum* | 4.3 |
| *Diplostephium serratifolium* | 1.4 |
| *Diplostephium oblanceolatum* | 2.5 |
| *Aztecaster matudae* | 1.9 |
| *Linochilus cinerascens* | 8.5 |
| *Linochilus rupestris* | 3.3 |
| *Linochilus jaramilloi* | 2.5 |
| *Linochilus juajibioyi* | 1.5 |
| *Linochilus ochraceus* | 1.9 |
| *Linochilus frontinensis* | 3.7 |
| *Erigeron breviscapus* | 13.1 |
| *Erigeron multiradiatus* | 6.6 |
| *Aster altaicus* | 6.9 |
| *Aster flaccidus* | 4.2 |
| *Artemisia ordosica* | 12.4 |
| *Artemisia scoparia* | 3.3 |
| *Opisthopappus taihangensis* | 4.4 |
| *Dendrosenecio cheranganiensis* | 6.5 |
| *Cynara humilis* | 9.8 |
| *Atractylodes macrocephala* | 7.3 |
| *Saussurea chabyoungsanica* | 6.8 |
| *Saussurea involucrata* | 4.0 |
| *Saussurea lhozhagensis* | 4.3 |
| *Burmeistera pirrensis* | 0.8 |
| *Burmeistera lutosa* | 0.2 |
| *Burmeistera smaragdi* | 0.8 |
| *Lonicera x heckrottii* | 1.5 |
| *Viburnum farreri* | 1.1 |
| *Angelica acutiloba* | 1.3 |
| *Angelica porphyrocaulis* | 1.5 |
| *Semenovia transiliensis* | 0.3 |
| *Bupleurum euphorbioides* | 0.2 |
| *Ligusticum hispidum* | 1.7 |
| *Ostericum palustre* | 0.2 |
| *Ostericum citriodorum* | 0.4 |
| *Panax zingiberensis* | 2.2 |
| *Eleutherococcus senticosus* | 1.2 |
| *Eleutherococcus brachypus* | 1.4 |
| *Fatsia japonica* | 0.4 |
| *Ilex pubescens* | 19.5 |
| *Ilex asprella* | 0.4 |
| *Adenocalymma biternatum* | 0.6 |
| *Anemopaegma prostratum* | 0.4 |
| *Salvia honania* | 1.8 |
| *Nepeta hemsleyana* | 0.3 |
| *Ocimum gratissimum* | 0.8 |
| *Phyllostegia velutina* | 0.9 |
| *Lagotis brevituba* | 0.6 |
| *Utricularia tenuicaulis* | 4.7 |
| *Buddleja alternifolia* | 0.3 |
| *Chionanthus filiformis* | 0.3 |
| *Noronhia lowryi* | 0.5 |
| *Noronhia intermedia* | 0.5 |
| *Comoranthus minor* | 1.4 |
| *Forestiera isabelae* | 0.6 |
| *Abeliophyllum distichum* | 0.3 |
| *Forsythia suspensa* | 0.7 |
| *Hoya pottsii* | 0.3 |
| *Gentiana stipitata* | 1.3 |
| *Solanum trilobatum* | 0.2 |
| *Nicotiana attenuata* | 0.6 |
| *Primula sikkimensis* | 1.1 |
| *Embelia vestita* | 1.9 |
| *Styrax dasyanthus* | 8.7 |
| *Alniphyllum pterospermum* | 0.6 |
| *Actinidia callosa* | 0.3 |
| *Actinidia polygama* | 0.6 |
| *Camellia nitidissima* | 1.0 |
| *Stewartia sinii* | 0.5 |
| *Hydrangea moellendorffii* | 0.6 |
| *Hydrangea barbara* | 0.5 |
| *Hydrangea ampla* | 0.5 |
| *Deutzia compacta* | 0.3 |
| *Cornus walteri* | 3.4 |
| *Cornus alternifolia* | 1.5 |
| *Cornus volkensii* | 0.4 |
| *Photinia prionophylla* | 0.8 |
| *Populus balsamifera* | 0.2 |
| *Salix integra* | 0.9 |
| *Byrsonima coccolobifolia* | 0.5 |
| *Juglans major* | 2.2 |
| *Alnus nepalensis* | 3.2 |
| *Trichosanthes nervifolia* | 1.5 |
| *Strigosella malacotricha* | 0.5 |
| *Ricotia cretica* | 0.2 |
| *Bunias erucago* | 0.2 |
| *Acer saccharum* | 1.6 |
| *Achyranthes aspera* | 0.9 |
| *Loranthus grewingkii* | 0.2 |
| *Phacellaria compressa* | 2.2 |
| *Dendrobium parciflorum* | 0.1 |
| *Liparis bootanensis* | 0.2 |
| *Cephalanthera rubra* | 0.2 |
| *Galearis cyclochila* | 0.3 |
| *Convallaria keiskei* | 0.3 |
| *Panicum capillare* | 0.2 |
| *Triodia schinzii* | 0.2 |
| *Aristida rufescens* | 0.3 |
| *Dioscorea sansibarensis* | 0.6 |
| *Aletris spicata* | 0.1 |
| *Tulipa buhseana* | 0.4 |
| *Clematis acerifolia* | 0.2 |
| *Beesia calthifolia* | 0.3 |
| *Dysosma difformis* | 1.2 |
| *Syndiclis kwangsiensis* | 0.3 |
| *Chengiodendron marginatum* | 0.7 |
| *Patellifolia patellaris* | 1.0 |
| *Aldama macrorhiza* | 2.3 |
| *Ligusticum thomsonii* | 0.3 |
| *Pisum abyssinicum* | 0.7 |
| *Silene conica* | 0.1 |
| *Sporobolus helvolus* | 19.8 |
| *Urochondra setulosa* | 13.0 |
| *Dactyloctenium aegyptium* | 0.9 |
| *Triodia wiseana* | 3.3 |
| *Orinus kokonoricus* | 0.9 |
| *Loudetiopsis kerstingii* | 1.5 |
| *Dichaetaria wightii* | 0.9 |
| *Aristida stipitata* | 1.6 |
| *Sartidia perrieri* | 1.2 |
| *Dendrocalamus barbatus* | 11.7 |
| *Dendrocalamus latiflorus* | 1.3 |
| *Panicum sumatrense* | 1.7 |
| *Gynerium sagittatum* | 1.3 |
| *Stipagrostis hirtigluma* | 0.3 |
| *Rehia nervata* | 0.2 |
| *Leymus komarovii* | 0.7 |
| *Hygroryza aristata* | 0.1 |
| *Dendroseris pruinata* | 12.7 |
| *Bidens torta* | 1.8 |
| *Diplostephium haenkei* | 1.9 |
| *Artemisia argyi* | 87.2 |
| *Dipelta yunnanensis* | 0.4 |
| *Chionanthus compactus* | 0.6 |
| *Paulownia fargesii* | 0.7 |
| *Ardisia mamillata* | 1.1 |
| *Camellia achrysantha* | 0.8 |
| *Vachellia flava* | 1.2 |
| *Salix babylonica* | 0.5 |
| *Krameria lanceolata* | 0.9 |
| *Alyssopsis mollis* | 0.3 |
| *Acer circinatum* | 0.7 |
| *Salicornia brachiata* | 17.5 |
| *Salicornia europaea* | 11.3 |
| *Suaeda japonica* | 4.7 |
| *Suaeda malacosperma* | 2.1 |
| *Pseudostellaria heterophylla* | 0.9 |
| *Cymbidium tracyanum x Cymbidium faberi* | 3.4 |
| *Gymnadenia conopsea* | 0.2 |
| *Hosta clausa* | 4.1 |
| *Polygonatum inflatum* | 1.5 |
| *Maianthemum henryi* | 0.8 |
| *Ampelocalamus naibunensis* | 0.3 |
| *Neolitsea pallens* | 0.2 |
| *Malus x atrosanguinea* | 5.9 |
| *Amelanchier arborea* | 2.7 |
| *Rhamnus heterophylla* | 1.8 |
| *Citrullus lanatus* | 5.2 |
| *Aldama kunthiana* | 2.1 |
| *Saussurea hookeri* | 2.6 |
| *Viburnum odoratissimum* | 0.3 |
| *Nicotiana debneyi* | 1.0 |
| *Camellia grandibracteata* | 3.4 |
| *Dendrobium exile* | 0.6 |
| *Ophiopogon japonicus* | 0.2 |
| *Phyllorachis sagittata* | 0.1 |
| *Cymbopogon citratus* | 0.3 |
| *Asteropyrum cavaleriei* | 0.1 |
| *Papaver rhoeas* | 0.4 |
| *Verbena officinalis* | 0.4 |
| *Oreorchis patens* | 0.3 |
| *Phragmipedium kovachii* | 0.2 |
| *Beilschmiedia glauca* | 0.3 |
| *Lobularia libyca* | 0.2 |
| *Aldama nudibasilaris* | 17.6 |
| *Hesperomeles pernettyoides* | 3.1 |
